# Thermodynamic limits in far-from-equilibrium molecular templating networks

**DOI:** 10.1016/j.newton.2025.100302

**Published:** 2026-01-05

**Authors:** Benjamin Qureshi, Jenny M. Poulton, Thomas E. Ouldridge

**Affiliations:** 1 Department of Bioengineering, Imperial College London, London SW7 2AZ, UK; 2 Centre for Engineering Biology, Imperial College London, London SW7 2AZ, UK; 3 Department of Physics and Astronomy, University of Sheffield, Sheffield S3 7RH, UK

**Keywords:** stochastic thermodynamics, molecular networks, molecular templating, thermodynamics of information, non-equilibrium biophysics

## Abstract

Cells maintain a highly specific, far-from-equilibrium population of RNA and protein molecules. They do so via complex reaction networks in which templates catalyze the assembly of desired products. We show that information transmission from templates to products in such networks is bounded by functions of the maximal difference in free-energy changes between assembly pathways. Surprisingly, putative systems operating at the bounds do not have a high net flux around the network, as is typical in far-from-equilibrium systems and observed in biology. Instead, the upper bound on accuracy for a given network structure is achieved in “pseudo-equilibrium.” Here, each product is produced and degraded by time-reversed trajectories along a single (product-specific) pathway with negligible entropy production; product yields are determined by the free-energy changes along those pathways. The limit imposed by these free-energy changes induces a thermodynamic constraint on accuracy, even if a single templating process is arbitrarily kinetically selective.

## Highlights

• Derivation of fundamental bounds on the accuracy of molecular templating networks
• Identification of unexpected pseudo-equilibrium behavior at these bounds
• Resolution of the paradox surrounding the necessary costs of accurate templating

## Accessible overview

Cells assemble complex molecules such as proteins using template polymers with information-carrying sequences. These sequences are used to direct the ordered assembly of a set of molecular building blocks to form exactly the right product, such as a specific protein, on demand. A crucial feature of this process is that the templates act catalytically, since they accelerate the assembly of a specific product without being consumed in the process. A single template can then be used to direct the assembly of many copies of a product. It has long been suspected that this transfer of information from catalytic templates to products must be associated with a minimal energy cost, in the same way that computation has lower bounds on energy input. To explore this hypothesis further, this work considers arbitrarily complex templating networks involving many assembly and disassembly processes. We show that the accuracy with which a specific set of products can be maintained is, indeed, constrained by energetic properties of the underlying network. Surprisingly, the operation of the most efficient templating networks is completely different from the behavior observed in nature, where molecules such as proteins are overwhelmingly assembled by templates and degraded in a distinct, template-free fashion. Instead, the most efficient approach, which both achieves the highest accuracy and has a negligible energetic cost, is to disassemble products via a reversal of the pathway by which they were formed. This observation raises interesting questions about why nature does not operate in this way and whether synthetic templating systems can be built that exploit this approach.

## Graphical abstract

**Figure:**
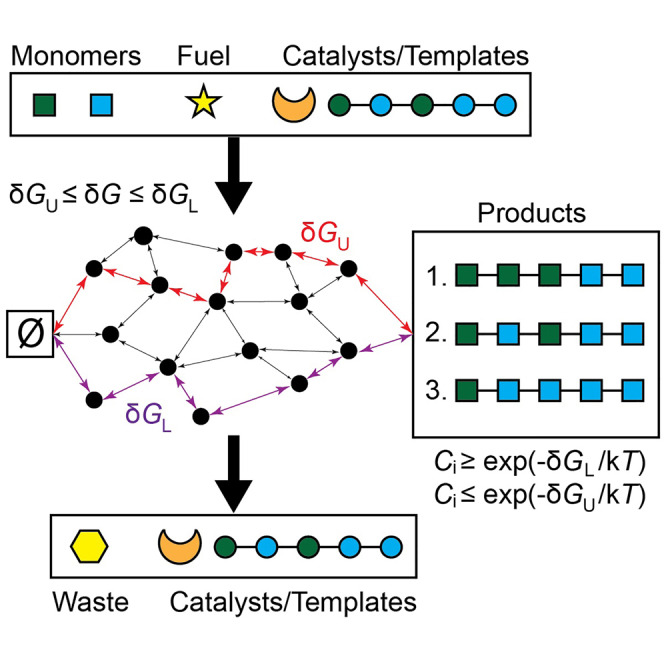

## Teaser

In cellular systems, nucleic acid templates catalyze the formation of protein and RNA molecules. Qureshi et al. analyze the behavior of catalytic molecular templating networks and bound the accuracy of such templating networks via functions of the maximal difference in free-energy changes between possible assembly pathways. Surprisingly, systems that operate at the bounds exist in a pseudo-equilibrium, balancing forward and backward transitions, unlike the behavior observed in biology.

## Introduction

Cellular mRNA and DNA molecules selectively catalyze the formation of many distinct protein and mRNA sequences from a small set of monomer building blocks.^1^^,^^2^ Both proteins and RNA are also actively degraded.^3^ The underlying processes are complex, with many pathways to assembly and disassembly and non-trivial motifs—such as kinetic proofreading^4^^,^^5^—within those pathways. The net result is a distribution of protein and RNA sequences in the cell, biased toward template-specified targets; sharply peaked, or *accurate*, distributions are essential for function.

The theory of such systems has generally focused on isolated templating events^6^^,^^7^^,^^8^^,^^9^^,^^10^^,^^11^^,^^12^^,^^13^^,^^14^^,^^15^^,^^16^^,^^17^^,^^18^^,^^19^ rather than full networks. Previous works^2^^,^^20^ have hypothesized that such networks would require a minimal entropy production per output polymer, determined by the sequence information transmitted from template to products. Recently, Genthon et al.^21^ modeled a network with competing templating and spontaneous degradation pathways, treating both as a one-step process with an imposed kinetic selectivity. They observed a phase transition to a high-accuracy regime with a large cyclic flux of templated production and spontaneous degradation, with a minimal fuel turnover per product made commensurate with the accuracy of the product ensemble, apparently confirming the predictions of Ouldridge and ten Wolde^2^ and Bennett.^20^ Relatedly, others have shown that the responsivity of steady states of complex networks to perturbations depends on the thermodynamic driving within the system,^22^^,^^23^ without relating the results to the concentrations of products in templating systems.

Given the simplicity of the system in Genthon et al.,^21^ the thermodynamics of arbitrarily complex molecular templating networks is underexplored. Moreover, previous works^2^^,^^20^^,^^21^ suggest a paradox. Entropy production is related to the relative rates of time-reversed trajectories^24^ rather than the relative rates of distinct processes. Although biochemistry may practically limit relative templating rates for matching and non-matching sequences, thermodynamics places no limit on this kinetic discrimination *in principle*. Why then, is accuracy associated with a minimal thermodynamic cost?^25^

Here, we first show that functions of a simple quantity—$\Delta \tilde{G}$, the *k*_B_*T*-normalized difference between the maximal and minimal free-energy changes along assembly pathways—bounds the accuracy of arbitrarily complex molecular templating networks. We then explore these bounds for a system in which *M* possible products can be formed. For *M*→*∞*, it is possible to maintain a steady-state ensemble with only a single product type, provided $\frac{\Delta \tilde{G}}{\ln\;M}>\;1$. By contrast, a single product necessarily has zero weight within the ensemble for $\frac{\Delta \tilde{G}}{\ln\;M}<\;1$. These results appear to confirm previous hypotheses^2^^,^^20^ and generalize the results from a recently published work.^21^ However, the bounds are more restrictive at finite *M*, and for *M*→*∞*, one can surprisingly maintain an ensemble dominated by a vanishingly small fraction of the possible products for any $\Delta \tilde{G}\gg 1$, even if $\Delta \tilde{G}\ll \ln\;M$, suggesting that a set of templates can be copied with high accuracy when a single one cannot.

Most significantly, however, systems that approach the $\Delta \tilde{G}$-dependent bounds do not have large, entropy-producing cyclic fluxes, as is common in far-from-equilibrium systems, anticipated in the case of templating networks,^2^^,^^20^^,^^21^ and observed in biology. Instead, each product overwhelmingly couples to a single pathway, balancing forward and backward transitions and exhibiting a yield determined by that pathway’s free-energy change. These bound-saturating “pseudo-equilibria” have negligible entropy production per assembly event and resolve the apparent paradox of the minimal cost of templating.

## Results

### A modeling framework for arbitrarily complex molecular templating networks

**Figure 1:**
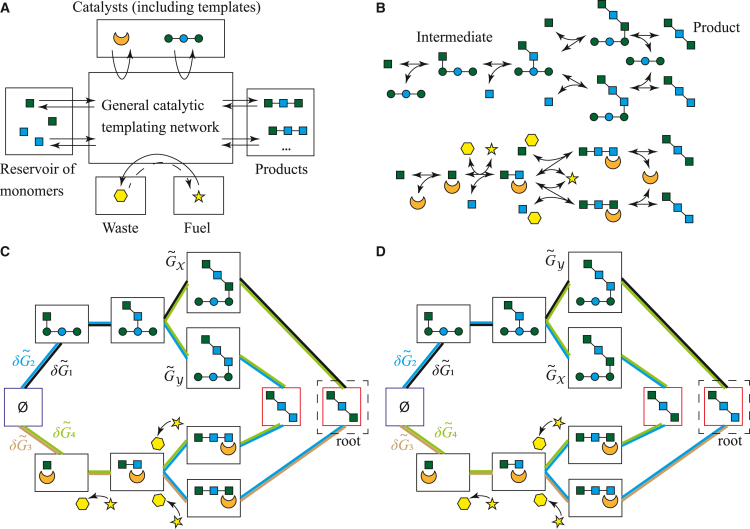
Modeling framework (A) Polymers are produced via catalyzed addition of monomers in an arbitrarily complex reaction network, driven by fuel consumption. (B) A subset of reactions in an example system, showing two branched pathways for production/degradation of two products, one involving a template catalyst and one involving another catalyst and fuel turnover. Equivalent sets of reactions, using the same fuels and catalysts (but different monomers), can produce both products shown. (C) Representation of the reactions in (B) as a graph, with nodes (rectangles), including a null complex Ø; intermediate species (black squares); and products (red squares). Each edge is a reversible transition, and each distinct pathway from Ø to the product has an associated normalized free-energy change $\delta {\tilde{G}}_{i}$ (illustrated here for the “root” product). (D) We take an alternative product as the root of the graph; the network connecting it to the null state is topologically identical to (C). The equivalent pathways for each product have the same overall $\delta {\tilde{G}}_{i}$, but intermediate free energies (and transition rates) can vary: for example, ${\tilde{G}}_{X}\neq {\tilde{G}}_{Y}$ in general.

We consider a broad class of networks, including that of Genthon et al.^21^ as a special case. As shown in Figure 1A, we consider the assembly of a set of monomer species (e.g., amino acids) into products. The “alphabet size” of this set of monomer species (2 in Figure 1) is arbitrary. These monomers can form *M* possible products. For clarity, we take these products to be linear polymers, as in transcription and translation, although the underlying theory does not require this assumption. The products may also be disassembled into monomers. These assembly and disassembly pathways are coupled to catalysts (e.g., ribosomes and proteases), some of which are sequence-bearing templates (e.g., mRNA), and can also involve turnover of fuel molecules into waste (e.g., ATP into ADP). In any given instance, we expect only a small fraction of the possible template species to be present.

To simplify analysis, we assume a symmetric model in which all products are polymers of a fixed length *L* ∼ ln *M*. Monomers are equivalent: they have the same free energy, and any sequence of monomers of the right length constitutes a product. As is common,^2^^,^^14^^,^^21^^,^^25^ we further assume the equivalence of the products, in the sense that they all contain the same number of monomers and have the same normalized standard free energy of formation, ${\tilde{G}}_{0}$, which would be expected to scale linearly with *L* ∼ ln *M* regardless of the templates present. This choice is the simplest one consistent with the fundamental fact that catalytic templates cannot change ${\tilde{G}}_{0}$.^2^^,^^14^ We will revisit these symmetry assumptions in the discussion.

We allow for an arbitrarily complex, thermodynamically self-consistent chemical reaction network (CRN) between the monomers and the products. A subset of reactions in a plausible network, forming two branched assembly/disassembly pathways through intermediates (any molecular complex excluding isolated monomers, products, catalyst, waste, or fuel), is given in Figure 1B. This example includes a trimer template that catalyzes the formation of a product with a matching sequence—although it is also possible to form the wrong sequence with a mismatched monomer—and a non-specific enzyme that consumes fuel to systematically degrade these products.

Aside from the existence of a reverse reaction for each forward reaction, which is required for thermodynamic self-consistency,^24^^,^^26^ we impose only two restrictions on the reaction network. First, no reactions occur between more than one product and/or intermediate. Second, due to our symmetry assumptions, if a set of chemical reactions whose net effect is to assemble a given product exists, a corresponding set of reactions involving the same fuels and catalysts exists for all products. For example, in Figure 1B, both the product that matches the template and one with a mismatch can form via the same template. Note that although these alternate reactions exist, they may be much slower due to selective catalysis by the templates.

We further assume that monomers are held at the same constant concentration (chemostatted) by the environment. Catalysts and fuels are also assumed to be chemostatted but at arbitrary concentrations. This approximation reflects the fact that in cells, monomers and fuel are generally abundant and their concentration maintained. However, the abundance of free mRNA, polymerase, or ribosome catalysts is more likely to be reduced by sequestration; we will therefore revisit the assumption that catalysts are chemostatted in the discussion.

### Representation of the templating network as a linear graph

We analyze these systems using deterministic CRNs^27^^,^^28^ obeying mass-action kinetics. CRNs consist of a set of species, a set of complexes (collections of species), and a set of reactions in which a complex X is converted into a complex Y at a rate given by a rate constant $k_{X\to Y}$ multiplied by the product of concentrations of the species in X.

Under our assumptions, only the products and intermediates are species in the CRN, with monomer, fuel, and free-catalyst concentrations acting as parameters that are absorbed into rate constants. Each complex then either contains a single species or is null (Ø); transitions from Ø correspond to the first association between monomers and catalysts. The CRNs are therefore linear and can be drawn as a graph; Figure 1C shows the graph formed by the possible subset of reactions in Figure 1B, with nodes as complexes and edges as reactions. The graph for the full network is connected since all complexes can be reached from Ø. This graph defines a set of self-avoiding walks (SAWs) from Ø to each product; each SAW and its inverse define a “pathway.” Pathways are illustrated by colored edges in Figure 1C. Our symmetry assumptions mean that all products are connected to ∅ by topologically equivalent graphs—this concept is illustrated in Figure 1D and formally defined in Note S1.

### Imposing self-consistent thermodynamics on the model

For thermodynamic self-consistency, we require that forward (X→Y) and reverse (Y→X) transitions obey local detailed balance^24^: $\delta {\tilde{G}}_{X\to Y}=-\ln\left(k_{X\to Y}/k_{Y\to X}\right)$, where $\delta {\tilde{G}}_{X\to Y}$ is the *k*_B_*T*-normalized standard free-energy change of the reaction.^26^ Here, $\delta {\tilde{G}}_{X\to Y}={\tilde{G}}_{Y}-{\tilde{G}}_{X}+\sum_{i}\tilde{\mu_{i}}\delta N_{i}^{X\to Y}$, with ${\tilde{G}}_{X}-{\tilde{G}}_{Y}$ representing the free-energy change of the non-chemostatted species and $\tilde{\mu_{i}}\delta N_{i}^{X\to Y}$ representing the free-energy change due to the production of $\delta N_{i}^{X\to Y}$ molecules of type *i*, chemostatted at a *k*_B_*T*-normalized chemical potential ${\tilde{\mu }}_{i}$. Each edge corresponds to a fixed $\delta N_{i}^{X\to Y}$, so $\delta {\tilde{G}}_{X\to Y}$ is well defined. However, two SAWs, *S* and *S*′, connecting Ø and a given product, such as the pink and brown SAWs in Figure 1C, can consume different amounts of fuel, allowing $\delta {\tilde{G}}_{S}\neq \delta {\tilde{G}}_{S^{\prime }}$, where $\delta {\tilde{G}}_{S}=\sum_{e\in S}\delta {\tilde{G}}_{e}$. The framework thus allows for arbitrarily complex non-equilibrium networks, incorporating separate production and degradation pathways and kinetic proofreading cycles.

Recalling that $\tilde{G_{0}}$ is the standard free energy of product formation, the total free-energy change along a SAW is then $\delta {\tilde{G}}_{S}=\tilde{G_{0}}+\sum_{e\in S}\sum_{i}\tilde{\mu_{i}}\delta N_{i}^{e}$. Since, based on our symmetry assumptions, all products are connected to Ø by topologically equivalent pathways involving the same fuel consumption, the set of free-energy changes associated with the formation of each product is also equivalent (this equivalence is illustrated in Figures 1C and 1D). By contrast, we allow for arbitrary intermediate free energies; for example, we expect ${\tilde{G}}_{X}\neq {\tilde{G}}_{Y}$ in Figures 1C and 1D due to the specificity of interactions with the template, and transition rates are unconstrained except by $\delta {\tilde{G}}_{X\to Y}=-\ln\left(k_{X\to Y}/k_{Y\to X}\right)$. Certain products can then form faster via kinetically selective templating.

We first show that, under these assumptions, properties of the steady-state distribution of products are bounded by functions of $\Delta \tilde{G}$, the difference between the maximum and minimum $\delta {\tilde{G}}_{S}$, for any self-consistent choice of rates. We will then explore the nature of these bounds.

### Bounds on the product ensemble

We consider the steady-state concentration of products (the “product ensemble”); our results also apply to the expected steady-state concentrations of a stochastic realization of the CRN.^29^ For linear, connected CRNs with a null complex, the steady-state concentration of any species is bound by the free-energy changes along the SAWs connecting that product state to Ø.^22^^,^^30^^,^^31^^,^^32^^,^^33^ Defining $\delta {\tilde{G}}_{L}^{Z_{i}}=\max_{S}\delta {\tilde{G}}_{S}^{Z_{i}}$ and $\delta {\tilde{G}}_{U}^{Z_{i}}=\min_{S}\delta {\tilde{G}}_{S}^{Z_{i}}$, where the optimization is performed over all SAWs that lead to *Z*_*i*_ from θ, then all concentrations obey the fundamental constraint $e^{-\delta {\tilde{G}}_{L}^{Z_{i}}}\leq c_{Z_{i}}\leq e^{-\delta {\tilde{G}}_{U}^{Z_{i}}}$ (see Note S2). For our networks, all products are connected to Ø by pathways with the same set of free-energy changes. All products therefore have the same template-independent upper and lower bounds ($e^{-\delta {\tilde{G}}_{U}},e^{-\delta {\tilde{G}}_{L}}$).

To see how these constraints manifest, we define the product distribution $P\left(Z_{i}\right)$ for products *Z*_*i*_, *i* = 1,⋯*M*:

(Equation 1) $$P\left(Z_{i}\right)=p_{i}=\frac{c_{Z_{i}}}{\sum_{j=1}^{M}c_{Z_{j}}}=\frac{c_{Z_{i}}}{c_{T}}\text{.}$$

Here, $c_{Z_{i}}$ is the concentration of product *Z*_*i*_ and $c_{T}=\sum_{j=1}^{M}c_{Z_{j}}$ is the total concentration. We consider two metrics for the deviation from a uniform equilibrium ensemble. First, the single-product specificity $p_{\max}=\max_{i}p_{i}$, which is a natural metric for the accuracy with which a single isolated template can influence the product ensemble. Second, we consider the (Shannon) entropy^34^ of the distribution

(Equation 2) $$H\left[p_{i}\right]=-\sum_{i=1}^{M}p_{i}\;\ln\;p_{i}=\ln\;c_{T}-\frac{1}{c_{T}}\sum_{i=1}^{M}c_{Z_{i}}\;\ln\;c_{Z_{i}}\text{.}$$

We consider this metric because $\ln\;M\;-\;H[p_{i}]$ is the channel capacity of the network if it is treated as an information channel from templates to products (see Note S4). Furthermore, this metric is better suited to systems (such as cells) in which a set of templates catalyzes the assembly of a number of distinct products in parallel. We describe optimizing for single-product specificity or entropy as specificity maximization or entropy minimization, respectively.

Given $e^{-\delta {\tilde{G}}_{L}}\leq c_{Z_{i}}\leq e^{-\delta {\tilde{G}}_{U}}$, specificity maximization at fixed $\Delta \tilde{G}=\delta {\tilde{G}}_{L}-\delta {\tilde{G}}_{U}$ is saturated by an ensemble with one species at *p*_max_ and all others at *p*_low_, with

(Equation 3) $$p_{\max}={\left(1+\left(M-1\right)e^{-\Delta \tilde{G}}\right)}^{-1},p_{\text{low}}=e^{-\Delta \tilde{G}}p_{\max}\text{.}$$

This $p_{\max}\left(\Delta \tilde{G}\right)$ thus defines a bound. Entropy minimization yields *H*[*p*_*i*_] ≥ *H*(*m*), with

(Equation 4) $$H\left(m\right)=\frac{\left(M-m\right)\Delta \tilde{G}e^{-\Delta \tilde{G}}}{m+\left(M-m\right)e^{-\Delta \tilde{G}}}+\ln\left(m+\left(M-m\right)e^{-\Delta \tilde{G}}\right)\text{.}$$

*H*(*m*) corresponds to *m* species at the upper bound, $c_{U}=\exp\;\left(-\delta {\tilde{G}}_{U}\right)$, and *M* − *m* species at the lower bound, $c_{L}=\exp\;\left(-\delta {\tilde{G}}_{L}\right)$. Minimizing Equation 4 with respect to integer *m* yields the lower bound on entropy, $H_{\min}\left(\Delta \tilde{G}\right)$. *H*_min_ and the minimizing *m* = *m*_min_ and a proof of Equation 4 are given in Note S3. For illustrative purposes, in this manuscript, we use the approximation in Equation S17, which treats *m*_min_ as continuous when *m*_min_ > 1 (a slightly weaker bound).

### The bounds in the presence of kinetic proofreading

Surprisingly, internal cycles in the reaction network, a requirement for celebrated kinetic proofreading motifs,^4^^,^^5^^,^^10^ do not directly feature in the bounds derived, since SAWs cannot contain cycles by definition. Adding a proofreading loop to a process may affect $\Delta \tilde{G}$ by providing a new pathway with a maximal or minimal free-energy change, but the SAWs identified would always be loop free and correspond to a fixed free-energy change for product formation. The possibility of repeatedly undergoing a single dissipative cycle in an actual dynamic trajectory, consuming an arbitrary amount of molecular fuel, does not translate into an arbitrary $\delta \tilde{G}$ along a pathway. However, the existence of loops within the network may still help the system to achieve a better product distribution than would otherwise occur if practical constraints on transition rates prevent the system from reaching the $\delta \tilde{G}$-dependent limits.

### Specificity maximization and entropy minimization

**Figure 2:**
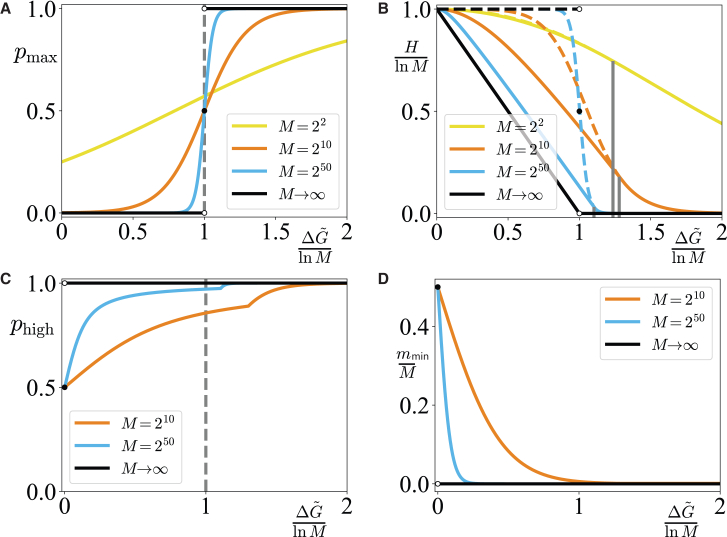
Behavior of single-product specificity and entropy bounds (A) The bound on single-product specificity *p*_max_ as a function of $\Delta \tilde{G}/\ln\;M$, for various *M*. (B) The bound on entropy *H*_min_ (solid lines) and the entropy of the distribution of maximal single-product specificity (dashed lines) as a function of $\Delta \tilde{G}$ for various *M* (for *M* = 2^2^, the lines nearly overlap). We scale both quantities by ln *M* so that they are defined per unit length. The dashed line is discontinuous at $\Delta \tilde{G}=\ln\;M$ for *M*→*∞*. The vertical (gray) lines show $\Delta \tilde{G}=\ln\;M+\text{ln ln }M$, below which the curves do not overlap. (C) The probability of selecting a product at the high concentration limit in the entropy-minimized ensemble, *p*_high_, as a function of $\Delta \tilde{G}/\ln\;M$, for various *M*. (D) The fraction of products at the high concentration limit in an entropy-minimized ensemble, *m*_min_/*M*, as a function of $\Delta \tilde{G}/\ln\;M$, for various *M*. (C) and (D) together show that a small fraction of products can dominate the ensemble, even below $\Delta \tilde{G}/\ln\;M=1$.

In Figure 2A, we plot the bound on *p*_max_ against $\Delta \tilde{G}/\ln\;M$ for various *M* (ln *M* is proportional to the length of a copolymer, and $\Delta \tilde{G}/\ln\;M$ therefore measures the free-energy difference per unit length). *p*_max_ undergoes a transition from *M*^−1^ to 1 as $\Delta \tilde{G}/\ln\;M$ is increased, centered on $\Delta \tilde{G}/\ln\;M=1$. As *M*→*∞*, the sigmoid becomes a phase transition.^21^ We note that this phase transition is qualitatively distinct from a traditional equilibrium order-disorder transition. Since all products have the same ${\tilde{G}}_{0}$, there is no energetic benefit to the “ordered” state with a single product. The system is necessarily enormously far from equilibrium, and any specificity is fundamentally a kinetic phenomenon.

Surprisingly, specificity maximization and entropy minimization are not equivalent. In Figure 2B, we plot the bound on *H*_min_ and the entropy of the maximum specificity distribution against $\Delta \tilde{G}/\ln\;M$. The two curves overlap for $\Delta \tilde{G}>\ln\;M+\text{ln ln }M+O\left(\frac{\text{ln ln }M}{\ln\;M}\right)$, for which values of $\delta \tilde{G}$ *m* = 1 minimizes Equation 4 and the lowest entropy state has a single product at a concentration $c_{U}=e^{-\delta {\tilde{G}}_{U}}$ and all others at $c_{L}=e^{-\delta {\tilde{G}}_{L}}$. For smaller $\Delta \tilde{G}$, the two differ drastically. *H*_min_ is obtained for distributions with *m*_min_ > 1 products at *c*_*U*_. This unexpected behavior arises because having *m* > 1 products at $e^{-\delta {\tilde{G}}_{U}}$ increases *c*_*T*_ and thus suppresses the probabilities of other species.

Figure 2C shows the total probability *p*_high_ of selecting a high-concentration product from the entropy-minimizing distribution, and Figure 2D shows the fraction *m*_min_/*M* of products at *c*_*U*_ in that ensemble. Although *m*_min_ increases and *p*_high_ decreases as $\Delta \tilde{G}\to 0$, for large *M*, *m*_min_/*M* tends to zero while maintaining *p*_high_ > 0.5 even for $\Delta \tilde{G}\ll \ln\;M$, well inside the region where single-product specificity is impossible. As *M*→*∞* at fixed $\Delta \tilde{G}/\ln\;M$, a vanishingly small proportion of the total number of possible products (*m*_min_/*M*→0) can dominate the ensemble (*p*_high_→1) even when $\Delta \tilde{G}\ll \ln\;M$. This result has consequences for systems in which a set of unrelated templates acts in parallel, as in cells. Although an ensemble of products dominated by copies of a single template is impossible below $\Delta \tilde{G}/\ln\;M=1$, an ensemble in which most products are accurate copies of a relatively small set of unrelated templates is possible.

### Physical meaning of the bounds

The phase transition in *p*_max_ at $\Delta \tilde{G}/\ln\;M=1$ for *M*→*∞* apparently generalizes the observations of Genthon et al.^21^ to arbitrarily complex networks and supports the hypotheses of Ouldridge and ten Wolde^2^ and Bennett,^20^ suggesting a minimal cost of accurately copying a single template. Notably, however, Figures 2C and 2D are hard to interpret in the context of a minimal cost of accuracy, as they suggest that it is possible to reduce the relative abundance of erroneous products at low values of $\Delta \tilde{G}/\ln\;M$ simply by having a larger (but still relatively small) set of unrelated templates. Simultaneously, significantly larger values of $\Delta \tilde{G}/\ln\;M$ are required to give perfect accuracy for finite *M* (see Figure 2A). Consider the implications for the charging of a single tRNA with an amino acid, an example of templated dimerization. There are approximately *M* = 400 combinations of codons and amino acids, having grouped redundant codons. For a single charged tRNA to dominate the ensemble with an error rate of 10^−5^, one would need a $\Delta \tilde{G}$ of around 17 *k*_*B*_*T* or 1 ATP at 37°C, substantially higher than *k_B_T* ln *M* = *k*_*B*_*T*ln400 ≈ 6 *k*_*B*_*T*, the value implied by the large *M* limit.

**Figure 3:**
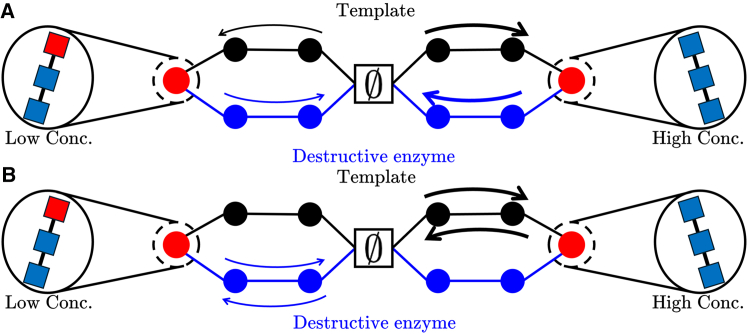
Contrasting a non-equilibrium steady state with a high cyclic flux and a non-equilibrium steady state in pseudo-equilibrium (A) A steady state with a high cycle flux. (B) A steady state in pseudo-equilibrium. In each case, the dominant production and degradation trajetories are shown.

Most importantly, a putative system that approaches the bounds on *p*_max_ and *H*[*p*_*i*_] behaves unexpectedly (Figure 3). $c_{Z_{X}}=\exp\;\left(-\delta {\tilde{G}}_{U}\right)$ can only be reached if *Z*_*X*_ is produced and degraded solely by the pathway with free-energy change $\delta {\tilde{G}}_{U}$; similarly, $c_{Z_{Y}}=\exp\;\left(-\delta {\tilde{G}}_{L}\right)$ requires *Z*_*Y*_ to be produced and degraded solely by the pathway with free-energy change $\delta {\tilde{G}}_{L}$. *Z*_*X*_ and *Z*_*Y*_ are then in pseudo-equilibria, with their yields equal to the equilibrium yield of the relevant pathway.

These pseudo-equilibria, whether or not they are practically achievable, limit all possible non-equilibrium templating systems for a given $\Delta \tilde{G}$. Indeed, any non-zero net flux necessarily *reduces* accuracy relative to this limit. Consider two products *X* and *Y* and assume *c*_*X*_ > *c*_*Y*_. Let $\Gamma_{X}\left(\delta \tilde{G}\right)$ be the observed ratio of production to degradation trajectories of *X* along a pathway with free-energy change $\delta \tilde{G}$; deviations of Γ from unity imply a net flux. At a steady state, thermodynamic self-consistency implies $\Gamma_{X}\left(\delta \tilde{G}\right)=\exp\;\left(-\delta \tilde{G}\right)/c_{X}$. Thus,

(Equation 5) $$\ln\left(c_{X}/c_{Y}\right)=\Delta \tilde{G}+\ln\left(\frac{\Gamma_{Y}\left(\delta {\tilde{G}}_{L}\right)}{\Gamma_{X}\left(\delta {\tilde{G}}_{U}\right)}\right)\text{.}$$

Since $\delta {\tilde{G}}_{U}$ corresponds to the most favorable production pathway, $\Gamma_{X}\left(\delta {\tilde{G}}_{U}\right)\geq 1$. Conversely, $\Gamma_{Y}\left(\delta {\tilde{G}}_{L}\right)\leq 1$. We see that a systematic flux to produce *X* along the most favorable production pathway or to degrade *Y* along the least favorable production pathway necessarily reduces the concentration ratio beneath that required to reach the bounds ($c_{X}=c_{Y}\;\exp\;\left(\Delta \tilde{G}\right)$ if *c*_*X*_ > *c*_*Y*_). Moreover, Equation 5 shows that the degree to which a system deviates from pseudo-equilibrium behavior, as measured by the Γ ratios, quantitatively dictates the relative concentrations of species and therefore the accuracy. Both the bound itself and the nature of systems that reach the bound are, unusually, directly relevant to the accuracy of systems operating far from the bound, such as those in nature.

Equally surprisingly, while operating at this pseudo-equilibrium limit requires a certain $\Delta \tilde{G}$ for accuracy, this “cost” is not an entropy production per product made, as it would be for a system with a high cyclic flux. If typical production and degradation events are truly time-reversed processes, the system will have zero net fuel consumption; all fuel consumed in production would be regenerated during destruction, without the need for a costly external recycling process. Entropy generated per production or degradation event is thus zero in a steady state—at variance with the expectations of Ouldridge and ten Wolde,^2^ Bennett,^20^ and Genthon et al.^21^

Fundamentally, these observations all follow because templating’s limiting “cost” is not needed to ensure the kinetic selectivity of a template in isolation—the ratio of catalytic rates is unconstrained by the second law. Rather, $\Delta \tilde{G}$-dependent bounds arise because steady-state yields cannot vary by more than the pseudo-equilibria arising from maximally different pathways. This observation explains why accurate templating systems have a minimal cost despite apparently only needing to distinguish between processes that are not time-reversed trajectories. It also explains why multiple templates can apparently be copied with more accuracy than a single one. If accuracy were constrained by a limit on template selectivity, adding more (unrelated) templates to the system would increase the total concentration of both desired and undesired products. In fact, however, at maximal accuracy, the undesired products equilibrate with the pathway of maximal $\delta \tilde{G}$, and so adding new pathways with low $\delta \tilde{G}$ does not increase their minimal concentration.

Pseudo-equilibrium limits only apply in the steady state, and no lower bound on $\Delta \tilde{G}$ is required for accuracy outside of the steady state. Consider a system with two products: $Ø\overset{k_{X}}{\underset{k_{X}}{⇌}}X,Ø\overset{k_{Y}}{\underset{k_{Y}}{⇌}}Y\text{.}$ The steady-state ensemble is unbiased, *p*_*X*_ = *p*_*Y*_ = 1/2, since $\Delta \tilde{G}=0$. However, *c*_*X*_ and *c*_*Y*_ can be very different at short times. Starting with initial conditions *c*_*X*_(0) = *c*_*Y*_(0) = 0, $\lim_{t\to 0}p_{X}\left(t\right)=\frac{k_{X}}{k_{X}+k_{Y}}\text{,}$ which is only bounded by 0 and 1. A catalyst that prefers *X* can temporarily achieve arbitrary specificity in the product ensemble even though $\Delta \tilde{G}=0$, as demonstrated experimentally in Cabello-Garcia et al.^35^

## Discussion

We have derived thermodynamic bounds on the accuracy of arbitrarily complex catalytic molecular templating networks and shown that systems at these bounds operate in pseudo-equilibrium with negligible cyclic flux. The fundamental $\Delta \tilde{G}$-dependent bounds on accuracy provide a basis on which to build our understanding of molecular information transmission, even if they are hard to achieve in practice. Indeed, Equation 5 shows how our fundamental bounds directly influence the accuracy of templating systems operating far from pseudo-equilibrium, as in biology.

Why, then, do cells not operate in pseudo-equilibrium if it is an efficient and accurate limit? First, we have derived bounds, not constructed realistic systems that approach them. In Note S5 and Figures S1 and S2, we give an explicit kinetic model of a templating network that saturates the bounds as rate constants are tuned. However, the network is contrived, and it is hard to imagine a system in which, for example, production and degradation by an RNA polymerase are balanced. Moreover, not all networks can reach the bounding accuracy, even if the rate constants in a network can be tuned arbitrarily. We provide an example in Note S6 and Figures S3–S7.

When considering the plausibility of pseudo-equilibria, it is worth noting the generality of the principle. The role of pseudo-equilibria in other contexts, such as the responsive systems of Arunachalam and Lin^22^ and Floyd et al.,^23^ is currently underexplored. As we illustrate in Note S7, similar results apply to networks in which substrates are activated and deactivated by catalysts, like kinase push-pull networks.^36^ In these settings, balancing activation and deactivation by a single (much less complicated) pathway is more plausible. Nonetheless, there are also disadvantages to operating in the pseudo-equilibrium limit, which may explain why natural kinase cascades do not operate in this way. First, outputs are binary: the steady-state concentration does not depend on the precise template concentration. Biological systems likely benefit from analog control. Second, switching between high and low pseudo-equilibria likely requires the removal of one catalyst and the introduction of another rather than just changing the levels of a single catalyst. Synthetic information-processing systems, however, may have different requirements from nature: a binary output is often preferable, products may be relatively simple, and fast switching may not be important. Synthetic pseudo-equilibrium networks may, therefore, hold promise; we illustrate a plausible nanotechnological system that could operate in pseudo-equilibrium, based on 4-way DNA strand exchange,^37^^,^^38^ in Figure S8.

We have noted that the behavior of catalytic molecular templating systems is qualitatively distinct from an equilibrium competition between a disordered, high-entropy state and a low-energy state. We now also compare our findings with previous results on discrimination during non-equilibrium polymerization. Aside from Genthon et al.,^21^ which we have discussed in detail, prior work has focused on the chemical work done and/or the entropy production when creating a single polymer; these models describe a single branched pathway within our framework. For example, Sartori and Pigolotti^15^ showed that an excess chemical work per monomer is required to continuously grow an infinite-length product with a non-equilibrium distribution of single-monomer copy errors. As we have shown elsewhere,^25^ this apparent minimal work instead contributes to a kinetic barrier when the separation of products from templates is explicitly considered, with the minimal work then determined by the properties of the monomer and product ensembles. In either case, these lower bounds are the minimal (reversible) work to change the state of the system, and they do not imply a need for separate pathways with distinct free-energy changes. By contrast, the constraints on $\Delta \tilde{G}$ that we have derived are requirements on the diversity of assembly pathways in a reaction network to maintain a steady-state ensemble with certain properties—a steady state that cannot even be described in a formalism that focuses on a single growth event.

We also consider how our results fit within the thermodynamic and kinetic discrimination paradigms in previous work.^39^ In a catalytic system, all discrimination is kinetic, even in the stationary state, since catalysts do not shift equilibria. Our claim that catalytic specificity is not limited by thermodynamics is simply that differences in barrier heights (*δ* in Sartori and Pigolotti^39^) can be arbitrarily large in principle. When *δ* is large, high discrimination is possible with low-entropy production.

Although we consider a broad class of networks, we have made a number of assumptions in our analysis. Firstly, we have assumed a symmetric model in which all products are equivalent. In a more general setting, reaction pathways, $\delta {\tilde{G}}_{U}^{Z_{i}}$ and $\delta {\tilde{G}}_{L}^{Z_{i}}$ would be product specific, and it would be easier to create distributions that favor certain stable products and harder in other cases (although given that cells need functional, rather than stable, products, it is unclear whether this asymmetry would be beneficial). Nonetheless, differences in $\delta \tilde{G}$ would still bound accuracy, and the limiting accuracy would still be obtained when products are in pseudo-equilibrium at either their maximum ($e^{-\delta {\tilde{G}}_{U}^{Z_{i}}}$) or minimum ($e^{-\delta {\tilde{G}}_{L}^{Z_{i}}}$) concentrations. This reasoning applies to ensembles with products of different lengths or folding free energies, non-product-symmetric network topologies, and even ensembles with prematurely released products.

We have also assumed that the networks are linear. The most likely violation of linearity is that sequestration of templates and catalysts influences their concentration rather than those concentrations being held constant by chemostatting. This non-linearity would make solving for steady-state concentrations of products much harder, but it has a surprisingly small effect on our conclusions.^40^ Firstly, the free energy of a formation pathway *S* remains well defined and is unaffected by sequestration of catalysts by intermediates. Secondly, were we to construct a model with fixed total concentration of each catalyst *j*, $d_{j}^{T}$, we would find a steady state with a reduced concentration of free catalysts $d_{j}^{*}\leq d_{j}^{T}$. However, from the structure of CRNs, that solution would necessarily be identical to one in which all catalysts were chemostatted at their respective free concentrations, $d_{j}^{*}$. As a consequence, any pathway free-energy-dependent constraint on product abundances that holds in all chemostatted systems also applies for fixed total catalyst concentrations. For catalytic templating networks, $\Delta \tilde{G}$ sets the same bounds on accuracy, and these bounds are reached in pseudo-equilibrium.

## Methods

Details regarding the methods are included in the results section, and further details of derivations can be found in the supplemental information.

## Resource availability

### Lead contact

Requests for further information and resources should be directed to and will be fulfilled by the lead contact, Thomas E. Ouldridge (t.ouldridge@imperial.ac.uk).

### Materials availability

This study did not generate new materials.

### Data and code availability

Code supporting the findings of this study is openly available at https://zenodo.org/records/15554491.

## Acknowledgments

We thank Pieter Rein ten Wolde for his comments on the manuscript. This work is part of a project that has received funding from the 10.13039/501100000781European Research Council (ERC) under the European Union’s Horizon 2020 research and innovation program (grant agreement no. 851910). T.E.O. was supported by a The Royal Society is the funder Research Fellowship (grant no. UF150067) and Fellowship Renewal (grant no. URF\R\211020).

## Author contributions

Conceptualization, B.Q., J.M.P., and T.E.O.; methodology, B.Q. and T.E.O.; investigation, B.Q.; writing – original draft, B.Q.; writing – review & editing, J.M.P. and T.E.O.; funding acquisition, T.E.O.; supervision, J.M.P. and T.E.O.

## Declaration of interests

The authors declare no competing interests.

## Supplemental information

Document S1. Figures S1–S8 and Notes S1–S7

Document S2. Article plus supplemental information
