# Supplementary material for: Thermodynamic limits in far-from-equilibrium molecular templating networks
Source: Newton. 2026 Jan 5;2(1):None. doi: 10.1016/j.newton.2025.100302 (PMC12769091; doi:10.1016/j.newton.2025.100302)
Supplement: Document S1. Figures S1–S8 and Notes S1–S7 [file mmc1.pdf]

NEWTON, Volume *2*

Supplemental information

Thermodynamic limits in far-from-equilibrium molecular templating networks

Benjamin Qureshi, Jenny M. Poulton, and Thomas E. Ouldridge

Supplemental Figures

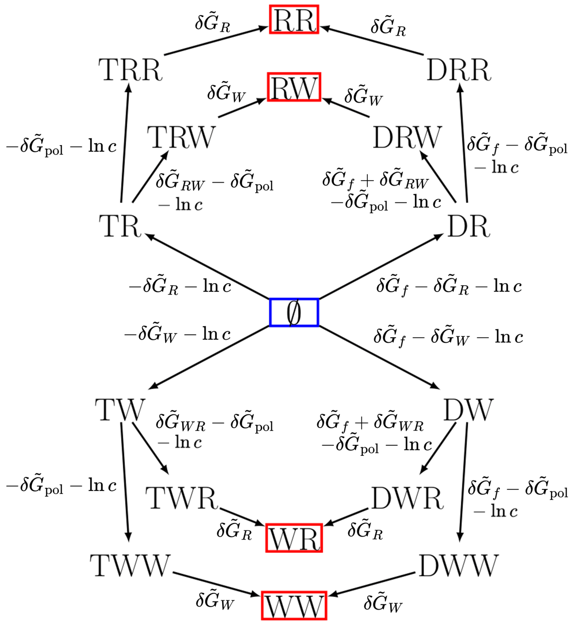

Figure S1: **Linearised CRN for a system in which dimers are grown/destroyed via a template “T” and by a destructive template “D”.** The system starts in the null state (blue) and a right or wrong (“R” and “W”) monomer attach to either the template “T” or destructive enzyme “D”. A second monomer can attach and polymerize with the first, yielding to a dimer that then detaches, giving the four red output states. Each arrow represents a reversible reaction, with the free-energy change in direction of the arrow indicated. For brevity, $\delta\tilde{G}_{RW} = -\delta\tilde{G}_{WR} = \delta\tilde{G}_R - \delta\tilde{G}_W$. To reach each red product node, there are four self-avoiding walks from the blue null state.

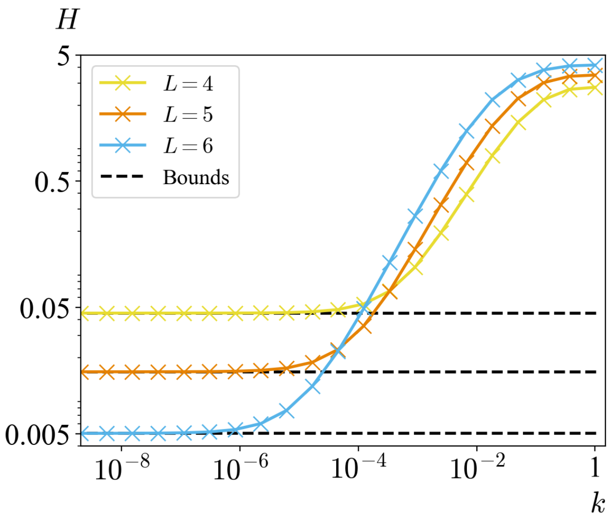

Figure S2: **The entropy bound may be saturated by model systems in the limit that some reaction rates are much smaller than others.** We plot the entropy, H, of the product distribution as a function of the slow reaction rates, k, for different template lengths, L, for the simple production and destruction model introduced in Note S5. The data is obtained for a fixed $\delta\tilde{G}_f = 2$, $\delta\tilde{G}_{\mathrm{pol}} = 0$, $\delta\tilde{G}_R = 2$, $\delta\tilde{G}_W = -2$ and $c = 1$. Note that longer templates reach a lower entropy bound for a fixed fuel turnover per unit length, $\delta\tilde{G}_f$.

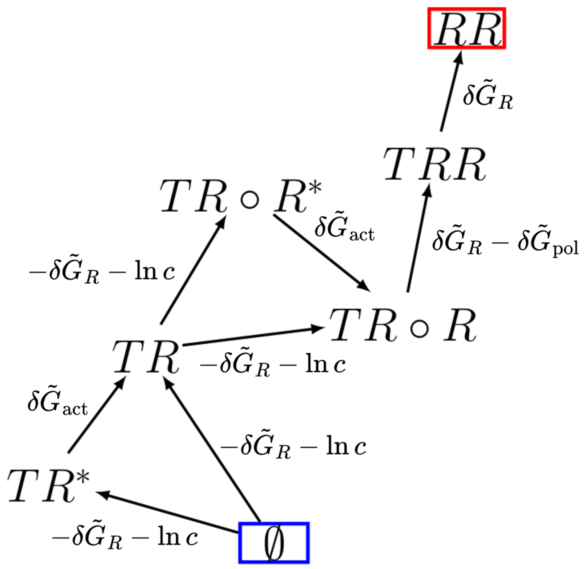

Figure S3: **A templating model incorporating kinetic proofreading.** We modify the template-based reactions of the model in Note S5. We show here the template-based reactions leading directly to the dimer RR, which should replace the equivalent template-based reactions in S1. As before, arrows represent reversible reactions with free-energy change in the direction of the arrow indicated. On the template, from state TR, either a non-activated (R^*) or activated (R) monomer may bind to the template. If that monomer has bound, but has not yet been polymerised into the growing polymer, it is represented by $TR \circ R^*$ or $TR \circ R$. When bound to the template, non-activated monomers may be activated, as shown by the transitions in which R^* is converted to R. When there is an activated monomer at the end of the growing polymer ($TR \circ R$), that monomer may be polymerised into the growing polymer to reach a polymerised state (TRR). After a full length polymer has grown on the template, it may detach to a product (RR for a dimer template).

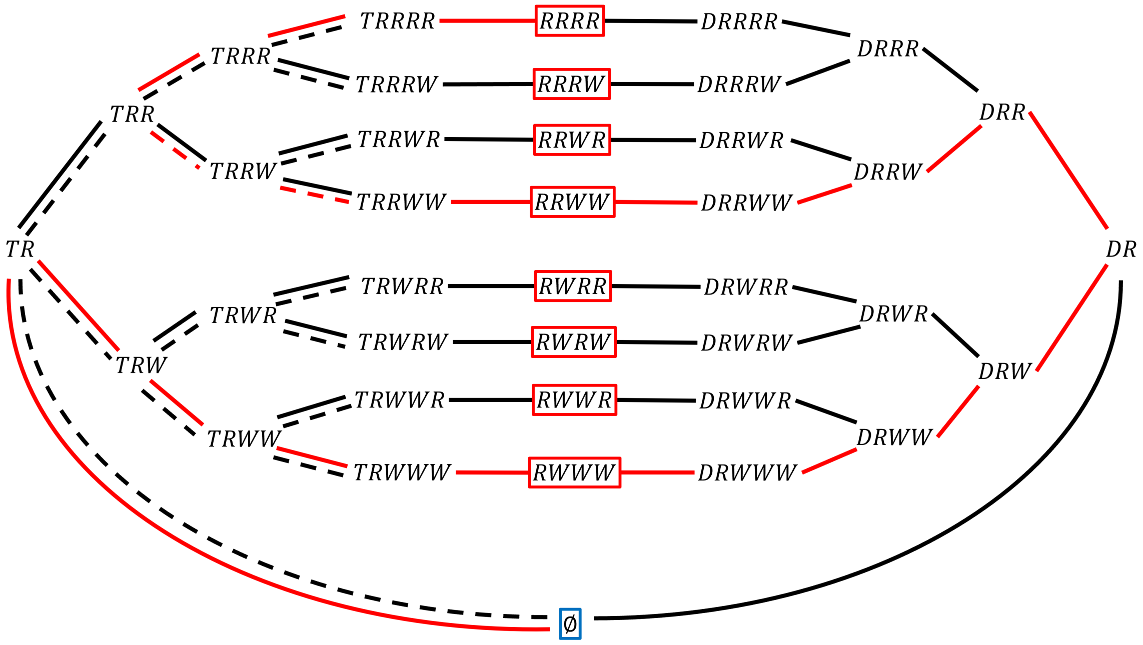

(a)

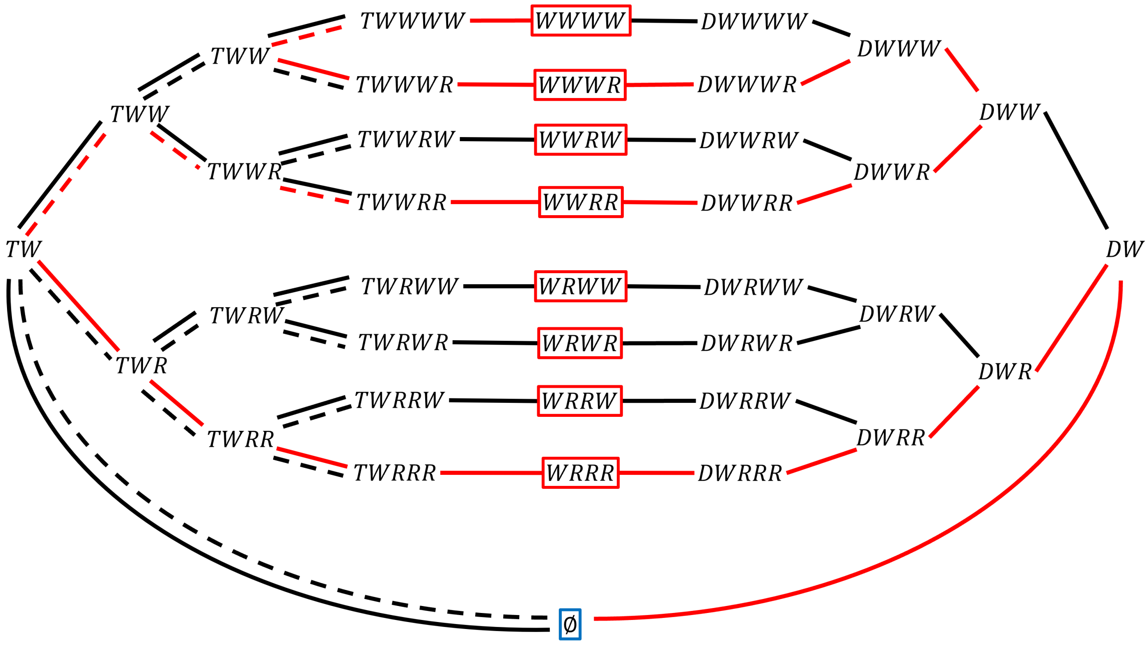

(b)

Figure S4: **Pathways with most positive and most negative free energy changes that we have identified in a model with kinetic proofreading.** (a) The pathway to $RRRR$ with free energy change $\delta\tilde{G}^*_U$ given by eq. S23 is shown in red. (b) The pathway to $WWWW$ with free energy change $\delta\tilde{G}^*_L$ given by eq. S24 is shown in red. For each of these diagrams, only the relevant half the reaction network is shown for simplicity. In these networks, there are two pathways between different template-bound states such as TRR and TRRR; one proceeding via R^* and one bia directly binding to R (figure S3). We represent these pathways via the solid and dashed lines, respectively.

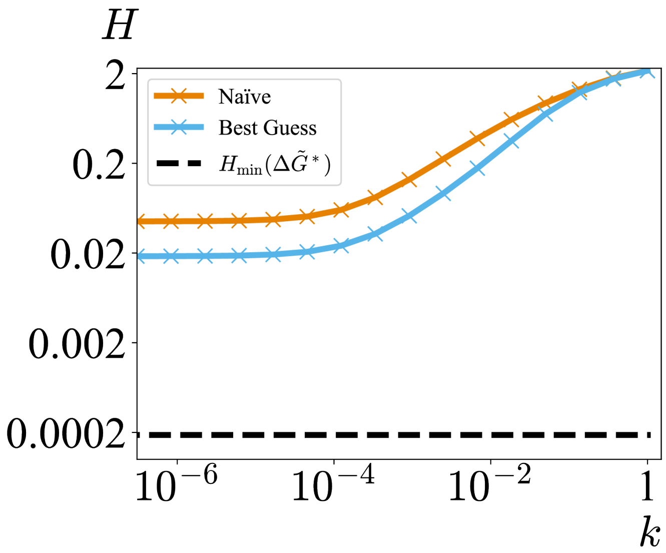

Figure S5: **Minimising product ensemble entropy in a model with kinetic proofreading.** Entropy H for two attempts to optimise the entropy, plotted alongside the lower bound $H_{\min}(\Delta\tilde{G}^*)$ for a system with $\Delta\tilde{G} = \Delta\tilde{G}^*$ implied by eqs. S23 and S24, as a function of the parameter k that sets the overall scale of slow reactions relative to fast ones. In the "naïve" approach, rates favour pathways to the correct product on the template and the incorrect ones on the destroyer. The "best guess" favours the snaking pathways described in text. The data is obtained for $\delta\tilde{G}_f = 1$, $\delta\tilde{G}_{\rm act} = 1$, $\delta\tilde{G}_{\rm pol} = 0$, $\delta\tilde{G}_R = 2$, $\delta\tilde{G}_W = -2$ and $c = 1$.

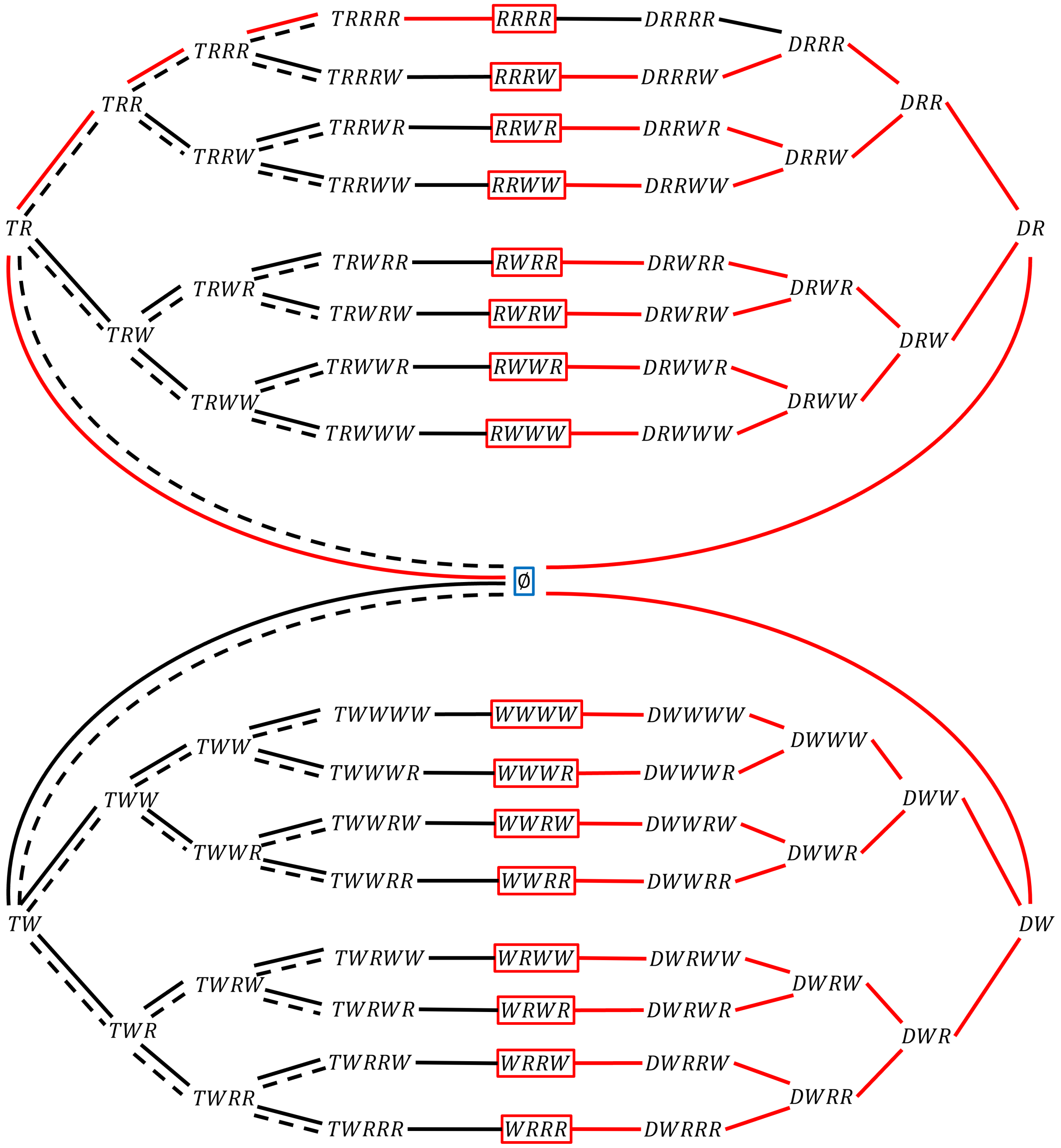

Figure S6: **Fast pathways in a naïve attempt to minimize the entropy of the product distribution for a model with kinetic proofreading.** Fast reactions with rate ~ 1 are shown in red. In these networks, there are two pathways between different template-bound states such as TRR and TRRR; one proceeding via R^* and one bia directly binding to R (figure S3). We represent these pathways via the solid and dashed lines, respectively.

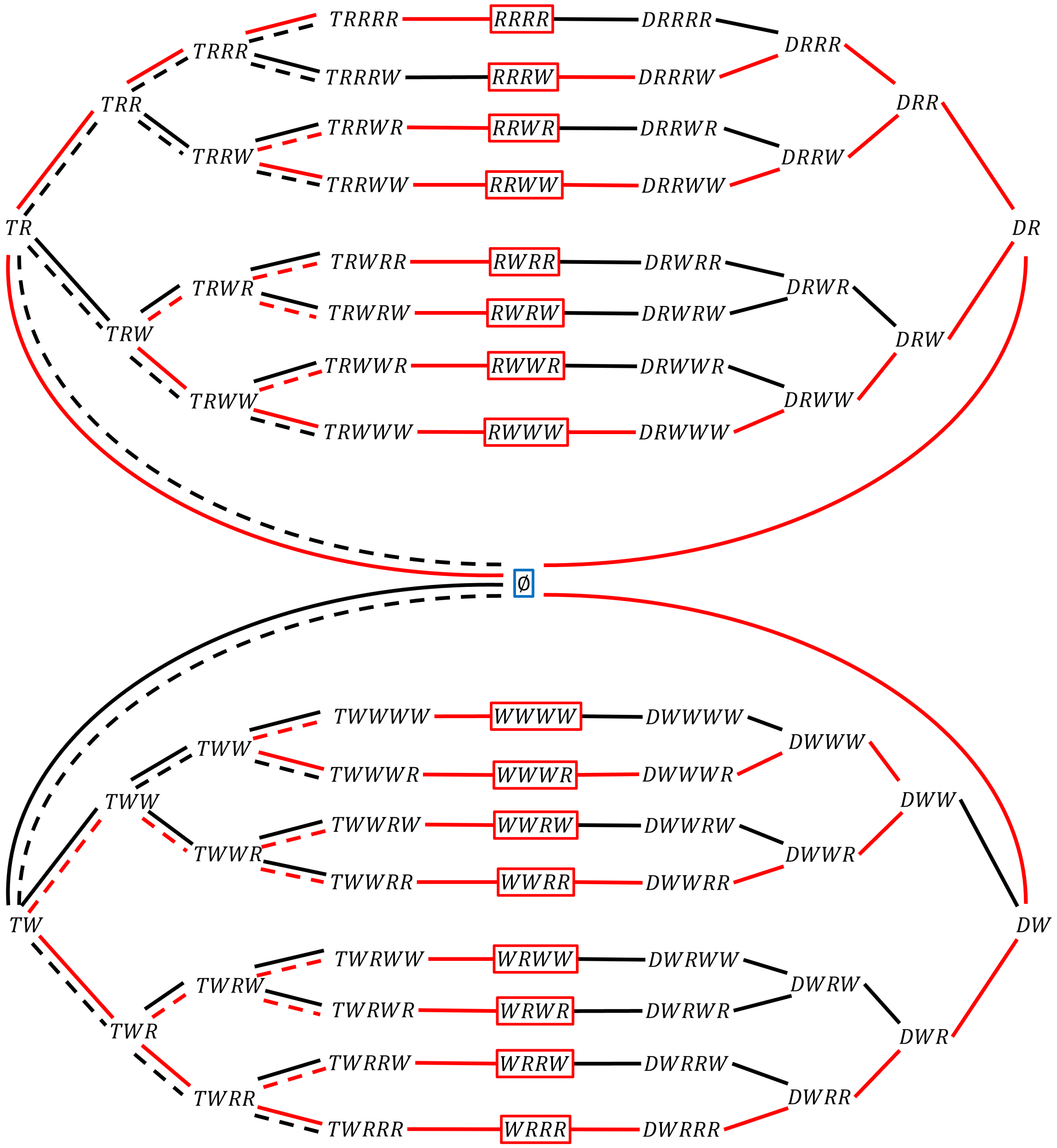

Figure S7: **Fast pathways in our best guess attempt to minimize the entropy of the product ensemble for a model with kinetic proofreading.** Fast reactions with rate ~ 1 are shown in red. In these networks, there are two pathways between different template-bound states such as TRR and TRRR; one proceeding via R^* and one bia directly binding to R (figure S3). We represent these pathways via the solid and dashed lines, respectively.

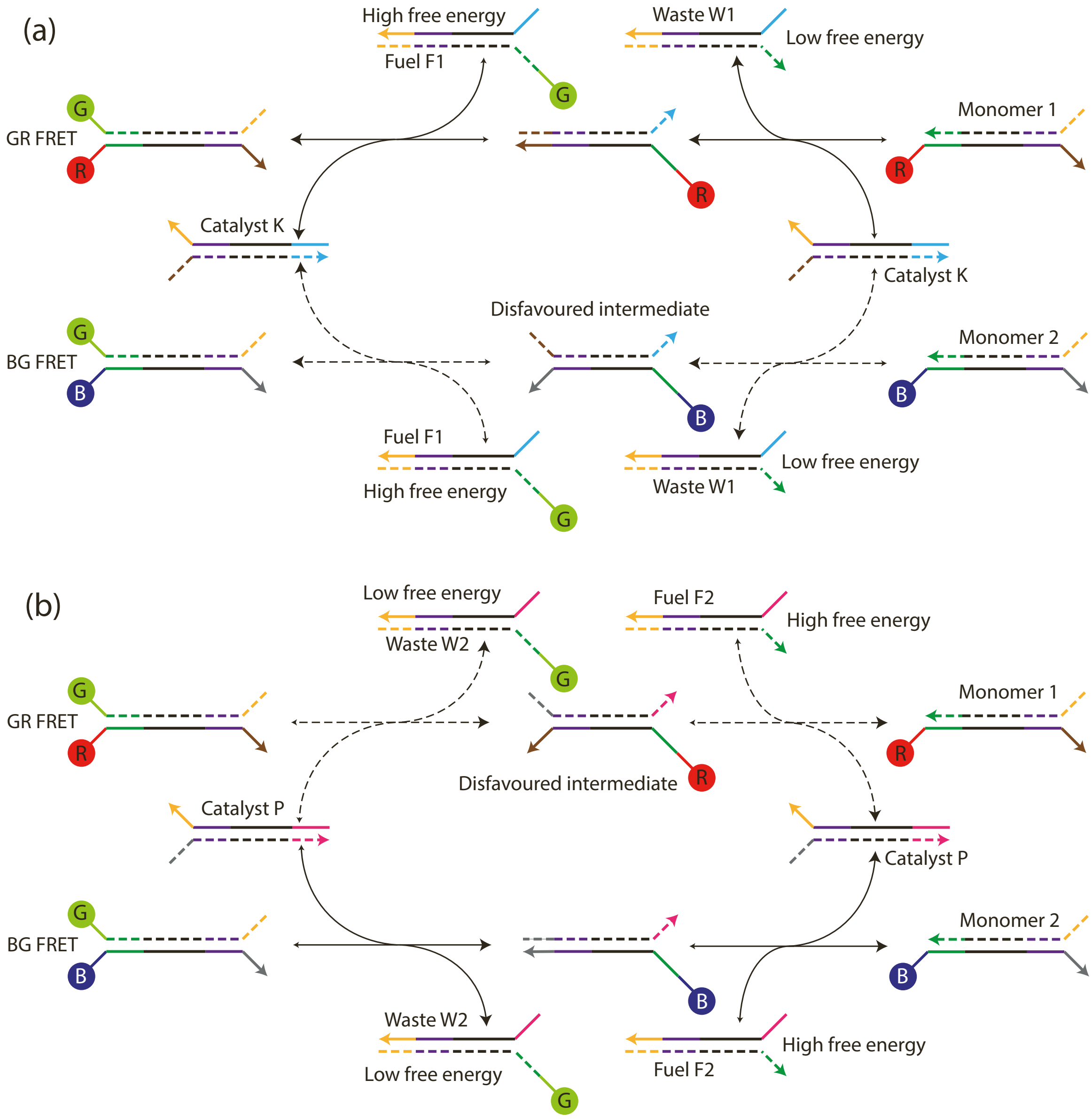

Figure S8: **A DNA strand displacement network, based on the ACDC design of Ref.[S1,S2], that is capable of demonstrating high specificity in pseudo-equilibrium.** Two monomer species (Monomer 1 and Monomer 2) can be activated and deactivated by fuel-coupled, catalytically controlled pathways. Activated species have colocalised fluorophores that can be identified by FRET signals. Lines represents DNA strands, and single-coloured sections of strands are domains of bases that act as a coherent unit, with solid sections of one colour being complementary to dashed sections of the same colour. Fluorophores are additionally labelled with R/B/G for clarity. Arrowheads on the DNA strands indicate $5' \to 3'$ directionality. Each reaction occurs via 4-way strand exchange[S1,S2] initiated by the binding of available and complementary toeholds (the shorter domains, of length ~ 5 bases). The size of the black reaction arrows indicate whether a reaction is driven forwards or backwards by the free energy difference between the fuel and waste. (a) Activation of Monomer 1 and Monomer 2 by a DNA duplex catalyst K coupled to the consumption of fuel F_1 and the production of waste W_1. The reaction is driven to the left (activation is thermodynamically favoured) by an excess of F_1 over W_1 and/or mismatches in the initial F_1 duplex that are eliminated in W_1[S1,S2]. Activation of Monomer 2 along this pathway has the same free-energy change as Monomer 1, but is kinetically very slow because the intermediate for Monomer 2 has a mismatched toehold domain. (b) A second pathway by which monomers can be interconverted between activated states, coupled to the catalyst P fuel F_2 and waste W_2. Unlike the reaction pathway in (a), the pathway in (b) is driven to the right and the catalyst P preferentially couples to Monomer 2 due to its toehold sequence.

Supplemental Notes

Note S1 Definition of topological equivalence

Let $\mathcal{G}$ be the vertex-labelled graph describing the linear CRN containing a vertex corresponding to the null complex $\emptyset$ and a set of vertices corresponding to products, ignoring the edge-weights. Then for any pair of products, X and Y, consider a relabelling of the nodes such that the nodes for X and Y swap labels, $\emptyset$'s label is not changed, but other node labels may be permuted. Suppose there exists such a relabelling, transforming the graph to a new vertex-labelled graph $\mathcal{G}'$ such that $\mathcal{G}'$ is isomorphic as a labelled graph to $\mathcal{G}$. Then the set of SAWs from $\emptyset$ to X will be topologically equivalent to those SAWs from $\emptyset$ to Y. Further, a given SAW from $\emptyset$ to X will be topologically equivalent to a specific SAW from $\emptyset$ to Y. If these equivalent SAWs also incur the same free-energy change, then the model falls into the framework of models we discuss in the paper.

Note S2 Proof of boundedness of steady-state concentrations

Note S2.A The steady state concentration written as sum over spanning trees

We first give a useful result for the concentration of a species in terms of the graph of the linear CRN. We note that a similar result can be seen in *e.g.*[S3]. However, we prove this result here with particular reference to the effect of a null species.

Consider a linear connected CRN under mass action kinetics. Let X_i be the chemical species with concentration c_i. Assume the CRN contains some reactions of the form $\emptyset \rightleftharpoons X$. Without loss of generality, we assume that there only exists at most one reaction of the form $X_i \to X_j$ (for the cases where there are multiple such reactions, replace their reaction rate with the sum over all reaction rates of reactions of that form). We may cast the steady state equation for the vector of steady state concentrations of the chemical species, $\mathbf{c}$, as the linear equation:

$$A\mathbf{c} + \mathbf{b} = \mathbf{0}, \tag{S1}$$

where $\mathbf{b}$ is a vector such that entry b_i is the rate constant of the reaction $\emptyset \to X_i$, and A is a matrix with off diagonal entries A_{ij} equal to the rate constant of the reaction $X_j \to X_i$ and diagonal elements A_{ii} equal to minus the sum of reaction rates of reactions $X_i \to X_j$ over all chemical species X_j $j \neq i$ as well as $X_i \to \emptyset$. Note that the sum of column i of matrix A is equal to minus the rate constant of reaction $X_i \to \emptyset$. Thus, we may create the new matrix:

$$K = \left(\begin{array}{c|c} A & \mathbf{b} \\ \hline \mathbf{d}^T & -\sum_i b_i \end{array}\right), \tag{S2}$$

where $\mathbf{d}$ is a vector such that entry d_i is equal to the rate constant of reaction $X_i \to \emptyset$. The columns of the matrix, K, now sum to zero and can be recognised as the Laplacian matrix of a certain graph. Represent K as a graph with nodes corresponding to chemical species X_i and an additional node corresponding to $\emptyset$, and edges e corresponding to reactions between species with weights equal to their rate constant $k(e)$. Then, by the matrix tree theorem**?** , the determinant (up to a sign) of the sub matrix formed by deleting row and column i from matrix K is given by the sum over the set of spanning trees rooted at node i, $\mathcal{T}(X_i)$,

$$\det(K/i) = \sum_{T \in \mathcal{T}(X_i)} \prod_{e \in T} k(e), \tag{S3}$$

where (K/i) represents matrix K with row and column i deleted. In particular,

$$\det(A) = \sum_{T \in \mathcal{T}(\emptyset)} \prod_{e \in T} k(e). \tag{S4}$$

Thus, by Cramers rule[S4], and the sign of the determinant under swapping of rows,

$$c_X = \frac{\sum_{T \in \mathcal{T}(X)} \prod_{e \in T} k(e)}{\sum_{T \in \mathcal{T}(\emptyset)} \prod_{e \in T} k(e)}, \tag{S5}$$

gives the solution to eq. S1.

Note S2.B Bounding steady-state concentrations

We note that similar proofs exist in the literature[S3,S5–S8], but we have included the proof here for completeness and to match our specific conventions. Further, in the cases considered in this paper, the fact that all products are connected to the null state means that our result bounds the absolute, rather than a relative, concentrations.

Consider a linear connected CRN with chemical species X_i and some reactions of the form $\emptyset \rightleftharpoons X_i$. The concentration of species X_i may be written as in eq S5. The numerator and denominator in this fraction are sums over spanning trees rooted at a given node. A sum over spanning trees rooted at node X may be factored into a sum over self avoiding walks (SAWs) from some arbitrary other node to node X. Concretely, letting Y be the other, arbitrary node, and $S(Y \to X)$ be the set of SAWs from Y to X,

$$\sum_{T \in \mathcal{T}(X)} \prod_{e \in T} k(e) = \sum_{S \in S(Y \to X)} A(S) \prod_{e \in S} k(e), \tag{S6}$$

where $A(S)$ is a factor that, crucially, is the same for the equivalent (reversed) SAW in $S(X \to Y)$, in which all edges are reversed compared to $S(Y \to X)$. That is to say, if we now wish to find the sum over spanning trees rooted at Y, we may choose X as the arbitrary other state and find:

$$\sum_{T \in \mathcal{T}(Y)} \prod_{e \in T} k(e) = \sum_{S \in S(Y \to X)} A(S) \prod_{e \in S} k(\bar{e}), \tag{S7}$$

where $\bar{e}$ is the reverse of edge e. For the linear CRNs, utilising

$$\delta \tilde{G}_S = -\ln\left(\prod_{e \in S} \frac{k(e)}{k(\bar{e})}\right), \tag{S8}$$

which follows from applying local detailed balance to each step of the SAW[S9], we may write:

$$\begin{aligned} c_X &= \frac{\sum\limits_{S \in S(\emptyset \to X)} A(S) \prod\limits_{e \in S} k(e)}{\sum\limits_{S \in S(\emptyset \to X)} A(S) \prod\limits_{e \in S} k(\bar{e})} \\ &= \frac{\sum\limits_{S \in S(\emptyset \to X)} A(S) \left[\prod\limits_{e \in S} k(\bar{e})\right] e^{-\delta \tilde{G}_S}}{\sum\limits_{S \in S(\emptyset \to X)} A(S) \prod\limits_{e \in S} k(\bar{e})}. \end{aligned} \tag{S9}$$

Hence,

$$c_X \in \left[e^{-\max\limits_{S \in S(\emptyset \to X)} (\delta G_S)}, e^{-\min\limits_{S \in S(\emptyset \to X)} (-\delta G_S)}\right], \tag{S10}$$

as required.

Note S3 Proof of the boundedness of steady-state distribution entropy

We have a set of M concentrations $\{c_1, \ldots c_M\}$. Denote the total concentration $c_T = \sum\limits_{i=1}^{M} c_i$. Suppose that each concentration is bounded by the same upper and lower bounds, $c_i \in [c_L, c_U]$. We now propose that the distribution of concentrations that minimises the Shannon entropy, $H([c_i])$, is that with $m_{\min}$ of the species at concentration c_U and $M - m_{\min}$ at concentration c_L, where $m_{\min}$ is either

$$\left\lfloor \frac{\frac{c_L}{c_U}\left[-\ln\left(\frac{c_L}{c_U}\right) - \left(1 - \frac{c_L}{c_U}\right)\right]}{\left(1 - \frac{c_L}{c_U}\right)^2} M \right\rfloor \text{ or }$$
$$\left\lceil \frac{\frac{c_L}{c_U}\left[-\ln\left(\frac{c_L}{c_U}\right) - \left(1 - \frac{c_L}{c_U}\right)\right]}{\left(1 - \frac{c_L}{c_U}\right)^2} M \right\rceil - 1. \tag{S11}$$

To prove this claim, let us calculate the derivative of $H = H([p_i])$ with respect to a concentration c_α, holding all other concentrations fixed and remembering that c_T is linear in c_α,

$$\frac{\partial H}{\partial c_\alpha} = \frac{1}{c_T}\left(-\ln\left(\frac{c_\alpha}{c_T}\right) - H\right). \tag{S12}$$

This H has a local maximum or minimum at $-\ln\left(\frac{c_\alpha}{c_T}\right) = H$. To proceed, we require the second derivative,

$$\frac{\partial^2 H}{\partial c_\alpha^2} = -\frac{1}{c_T^2}\left(-\ln\left(\frac{c_\alpha}{c_T}\right) - H\right) - \frac{1}{c_T}\frac{\partial H}{\partial c_\alpha} - \frac{1}{c_T}\frac{1}{c_\alpha}\left(1 - \frac{c_\alpha}{c_T}\right). \tag{S13}$$

Evaluating the second derivative at $-\ln\left(\frac{c_\alpha}{c_T}\right) = H$, the first two terms are zero and the third is necessarily negative for non-zero c_T. Thus

$$\left.\frac{\partial^2 H}{\partial c_\alpha^2}\right|_{-\ln\left(\frac{c_\alpha}{c_T}\right)=H} < 0. \tag{S14}$$

And so, for any distribution, we can decrease H by increasing the concentrations of any species i for which $-\ln\left(\frac{c_i}{c_T}\right) < H$ and decreasing the concentrations for any species whose concentration has $-\ln\left(\frac{c_i}{c_T}\right) > H$. For species for which $-\ln\left(\frac{c_i}{c_T}\right) = H$, changing the concentration in either direction will decrease H. Consequently, minimising the entropy of the distribution necessarily requires all species to be at one bound or the other: we need m species at concentration c_U and $M - m$ at concentration c_L. Hence, we have transformed the problem into a one dimensional one of minimising H as a function of m. We can write this entropy as

$$H(m) = -\frac{(M-m)\frac{c_L}{c_U}\ln\left(\frac{c_L}{c_U}\right)}{(M-m)\frac{c_L}{c_U} + m} + \ln\left((M-m)\frac{c_L}{c_U} + m\right). \tag{S15}$$

Taking the derivative with respect to m and setting it to zero tells us that

$$m_{\min} = \frac{\frac{c_L}{c_U}\left[-\ln\left(\frac{c_L}{c_U}\right) - \left(1 - \frac{c_L}{c_U}\right)\right]}{\left(1 - \frac{c_L}{c_U}\right)^2} M \tag{S16}$$

gives a turning point for $H(m)$, which is clearly a minimum since $H(0) = H(M) = \ln M$ is maximal entropy. Since $H(m)$ has only a single turning point as a function of m, the integer that minimises $H(m)$ will be either the floor or ceiling of the above expression, and $H_{\min}$ is given by eq. 4 of the main text and eq. S11 (remembering that $c_L/c_U = \exp(-\Delta\tilde{G})$).

A good approximation to the optimal value of m is given by

$$m'_{\min} = \max\left(\frac{e^{-\Delta\tilde{G}}\left[\Delta\tilde{G} - \left(1 - e^{-\Delta\tilde{G}}\right)\right]}{\left(1 - e^{-\Delta\tilde{G}}\right)^2} M, 1\right), \tag{S17}$$

since for $m_{\min} > 1$, the difference between using the integer value of $m_{\min}$ (eq. S11) and the continuous value $m'_{\min}$ (eq. S17) is small. In practice, using eq. S17 produces a very slightly looser bound on $H[p_i]$. Further, using eq. S17, for $m_{\min} > 1$, we may simplify:

$$H_{\min} = \ln M - \Delta\tilde{G}\left(1 + \frac{e^{-\Delta\tilde{G}}}{1 - e^{-\Delta\tilde{G}}}\right) + \ln\left(\frac{\Delta\tilde{G}}{1 + e^{-\Delta\tilde{G}}}\right) + 1. \tag{S18}$$

Note S4 Minimizing entropy maximizes channel capacity

We can consider the deterministic CRNs in this paper to be information channels. Let us assume the underlying chemistry, which determines the rate constants appearing in the un-linearized model, and the concentration of monomers, are fixed. The effective rates of the linearized network would then vary with the concentrations of catalysts only. As a specific (simple) example of how the entropy bound defines the channel capacity, let us assume that all variability is due to M sequence-specific template catalysts, one for each product, and that of these templates exactly one is present at any given time at a fixed concentration.

We can define the input to the information channel as the template that is present at high concentration; the output would then be a product sampled from the steady state product distribution for that input state. We can calculate the mutual information between the template input and output products. If the templates are symmetric, acting equivalently relative to their ideal sequence, then each output distribution would merely be a permutation of the set of product probabilities.

A system of this kind would define a symmetric channel. The channel capacity of such a symmetric channel[S10] is given by $C = \ln M - H([p_i])$ in our notation, where M is the number of products/sequence-specific templates, and $H([p_i])$ the Shannon entropy of the output distribution for any single template. This entropy will be the same for any input state in our symmetrized description. Hence, minimizing the entropy maximizes the channel capacity.

Note S5 Example chemical reaction network that can saturate the bounds on accuracy

Although calculations of the bounds on accuracy is often straightforward, evaluation of the actual performance of a system realisation can be more challenging. For the examples in Note S5 and Note S6, steady state distributions are found by numerical solution of the underlying ordinary differential equations (ODEs). The CRNs are linear, connected and contain only one stoichiometric compatibility class. Hence, there exists a single positive steady state to the ODEs induced by mass action kinetics[S11]. Initially, all concentrations are set to zero, and the ODEs are simulated for a large time until no change to the distribution is observed. For the results in Note S6, to speed up simulation, we make use a of a result from[S12] whereby we may coarse grain some sets of reactions without changing the steady state.

Refs[S12,S13] studied a system in which a polymer is grown on a template. Monomers of either the “right” or the “wrong” type are added one at a time to a polymer in contact with a template, while the product polymer continually unbinds from the template from behind its leading edge. We now extend the system to include final dissociation from the template for complete polymers, and also a “destructive template”. This destructive template participates in identical reactions to the template, except that polymerisation is driven backwards due to consumption of fuel molecules, meaning products tend to be destroyed rather than grown.

A full CRN for this model is shown below; we illustrate the linearised network (assuming monomers and catalysts are coupled to chemostats) for the case of dimerisation in figure S1.

$$
\begin{array}{rcl}
T + X_1 & \xrightleftharpoons[k^{T}_{X_1} e^{-\delta\tilde{G}_{X_1}}]{k^{T}_{X_1}} & TX_1 \\
TX_1\cdots X_{n-1} + X_n & \xrightleftharpoons[k^{T}_{X_1\cdots X_n} e^{-\delta\tilde{G}_{X_n}-\delta\tilde{G}_{\text{pol}}}]{k^{T}_{X_1\cdots X_n} e^{-\delta\tilde{G}_{X_{n-1}}}} & TX_1\cdots X_n \\
TX_1\cdots X_L & \xrightleftharpoons[k^{T,u}_{X_1\cdots X_L}]{k^{T,u}_{X_1\cdots X_L} e^{-\delta\tilde{G}_{X_L}}} & T + X_1\cdots X_L \\
D + X_1 & \xrightleftharpoons[k^{D}_{X_1} e^{\delta\tilde{G}_f-\delta\tilde{G}_{X_1}}]{k^{D}_{X_1}} & DX_1 \\
DX_1\cdots X_{n-1} + X_n & \xrightleftharpoons[k^{D}_{X_1\cdots X_n} e^{\delta\tilde{G}_f-\delta\tilde{G}_{X_n}-\delta\tilde{G}_{\text{pol}}}]{k^{D}_{X_1\cdots X_n} e^{-\delta\tilde{G}_{X_{n-1}}}} & DX_1\cdots X_n \\
DX_1\cdots X_L & \xrightleftharpoons[k^{D,u}_{X_1\cdots X_L}]{k^{D,u}_{X_1\cdots X_L} e^{-\delta\tilde{G}_{X_L}}} & D + X_1\cdots X_L,
\end{array}
\qquad \text{(S19)}
$$

for $n \leq L$, $X_i \in \{R, W\}$. Dynamics is assumed to follow mass action kinetics, with rate constants given above and below the harpoons. T represents the template, D the destructive catalyst and R, W the “right” and “wrong” monomers. The products are $X_1 \cdots X_L$, representing the polymers of length L. Here, there are $M = 2^L$ different products. $TX_1 \cdots X_n$ and $DX_1 \cdots X_n$ represent partial polymers, $X_1 \cdots X_n$, bound to the template or destructive catalyst. Monomer X binds to the template or destructive catalyst with standard free-energy $-\delta\tilde{G}_X$ and the standard free-energy of polymerisation is $-\delta\tilde{G}_{\text{pol}}$ in the absence of fuel. The destructive catalyst has an additional free energy $\delta\tilde{G}_f$ per length driving the disassembly of polymers.

The thermodynamics of the model are characterised by the standard polymerisation free energy of the monomers ($-\delta\tilde{G}_{\text{pol}}$); the standard free-energy change of binding to the template for right and wrong monomers ($-\delta\tilde{G}_R$ and $-\delta\tilde{G}_W$), and the free energy of fuel turnover $-\delta\tilde{G}_f$. We assume that both right and wrong monomers are held at concentration c.

For the model depicted in figure S1, we can calculate the free-energy change for different paths to each product state. For example, the paths from the null state to RR are:

$$
\begin{array}{lllllll}
1) & \emptyset & \to TR & \to TRR & \to RR, & \\
2) & \emptyset & \to TR & \to TRW & \to RW & \to DRW \\
 & & & \to DR & \to DRR & \to RR, \\
3) & \emptyset & \to DR & \to DRW & \to RW & \to TRW \\
 & & & \to TR & \to TRR & \to RR, \\
4) & \emptyset & \to DR & \to DRR & \to RR. &
\end{array}
\qquad \text{(S20)}
$$

These incur free-energy changes:

$$\begin{aligned}
&1) \quad -(\delta\tilde{G}_{\text{pol}} \quad +2\ln c),\\
&2) \quad -(\delta\tilde{G}_{\text{pol}} \quad +2\ln c),\\
&3) \quad -(\delta\tilde{G}_{\text{pol}} \quad +2\ln c) + \delta\tilde{G}_f,\\
&4) \quad -(\delta\tilde{G}_{\text{pol}} \quad +2\ln c) + 2\delta\tilde{G}_f.
\end{aligned} \tag{S21}$$

Each pathway contains the terms $-(\delta\tilde{G}_{\text{pol}} + 2\ln c)$, corresponding to the standard free-energy change of product formation without any fuel turnover. Since our bounds only rely on the differences in free energies between pathways, we may drop these contributions, and the fuel free energy $\delta\tilde{G}_f$ alone determines the bounds. For polymers of length L, the equivalent standard free energy of product formation is $(L-1)\delta\tilde{G}_{\text{pol}} + L\ln c$, and it too may be dropped for consideration of the bounds.

Our bounds are achieved when rates of the path with the most negative free-energy change are maximised for the desired product(s), and rates of the path with the most positive free-energy change are maximised for all other products. For the $L = 2$ case, for example, we could therefore maximise the rate of path 1 for RR, and the equivalents of path 4 for other products.

In fact, for this system, the entropy bound is formally achievable for arbitrary L. We can split the edges of the graph into two sets; one in which the rates are ~ 1, and one in which the rates are $\sim k$. In figure S2, we show $H[p_i]$ as $k \to 0$ for a particular choice of these sets, in which all the reactions leading directly to the fully correct sequence being created/destroyed on the template are set to be fast (not proportional to k), as are all the reactions leading directly to the creation/destruction of other sequences on the destroyer. Explicitly, set the rates of $\emptyset \to TR$, $TR \to TRR$, ..., $TR^{L-1} \to TR^L$, $TR^L \to R^L$ equal to 1 (here, R^L corresponds to L copies of R). We also set all the rates of $\emptyset \to DX_1$, $DX_1 \to DX_1X_2$, ..., $DX_1...X_{L-1} \to DX1...X_L$, $DX_1...X_L \to X_1..X_L$, where $X_i = R$ or W but excluding $X_1 \cdots X_L$ all being R, equal to 1. Conversely, we set the rates of $\emptyset \to DR$, $DR \to DRR$, ..., $DR^{L-1} \to DR^L$, $DR^L \to R^L$ and $\emptyset \to TX_1$, $TX_1 \to TX_1X_2$, ..., $TX_1...X_{L-1} \to TX_1...X_L$, $TX_1...X_L \to X_1..X_L$, where $X_i = R$ or W but excluding $X_1 \cdots X_L$ all being R, equal to $k_I = k$. The reverse reactions of those listed above have a rate determined by the free-energy change of reaction. For $k \to 0$, this set of reaction rates saturates the bound. The system saturates the entropy bound $H_{\min}$ as $k \to 0$. Note that for the value of $\delta\tilde{G}_f$ used, the minimal entropy and maximal specificity distributions are the same.

There are many possible ways to choose sets of edges that can saturate the bound in the limit $k \to 0$. Here, we have chosen a set of rates that specifically highlights a full pathway to each product for illustrative purposes. One might also wish to choose a minimum set of reactions to have rate constant k while still saturating the bound in the limit $k \to 0$. For example, letting the slow reactions be $DR^L \to R^L$, where R^L means L copies of R, and $TX_1...X_L \to X_1..X_L$, excluding $X_1 \cdots X_L$ all being R, will still saturate the bound in the limit $k \to 0$. Further, we note that it is possible to saturate the bound with a non-specific destructive catalyst, where the reaction rates are independent of the polymer sequence. In the examples we have identified, such a network requires at least three rate scales $\sim 1, k, k^2$.

We stress that although the bound is formally attainable in this system, doing so relies on the ability to manipulate rate constants arbitrarily, subject to thermodynamic constraints. In a more realistic model of a templating system, constraints on relative rates may also be relevant; these constraints may stop the system reaching the bounds on accuracy or product entropy.

Note S6 Example chemical reaction network with kinetic proofreading that cannot saturate the bounds on accuracy

To illustrate the application of the bound to a more complex network, and to demonstrate the possibility of non-trivial pathways defining the bound, we consider an extension to the previous model, wherein the template also performs kinetic proofreading. First suggested by Hopfield[S14] and Ninio[S15] and widely studied[S16,S17], kinetic proofreading is a mechanism by which a system can increase the specificity of a process by expending extra free energy through fuel consuming cycles. These cycles give an extra opportunity to reject the "wrong" monomers due to their shorter binding lifetime.

The full chemical reaction network for a proofreading template of arbitrary length L is:

$$
\begin{array}{rcl}
T + X_1^* & \xrightleftharpoons[k^{T,I}_{X_1} e^{-\delta\tilde{G}_{X_1}}]{k^{T,I}_{X_1}} & TX_1^* \\
TX_1^* & \xrightleftharpoons[k^{T,\text{act}}_{X_1}]{k^{T,\text{act}}_{X_1} e^{\delta\tilde{G}_{\text{act}}}} & TX_1 \\
T + X_1 & \xrightleftharpoons[k^{T}_{X_1} e^{-\delta\tilde{G}_{X_1}}]{k^{T}_{X_1}} & TX_1 \\
TX_1\cdots X_{n-1} + X_n^* & \xrightleftharpoons[k^{T,I}_{X_1\cdots X_n} e^{-\delta\tilde{G}_{X_n}}]{k^{T,I}_{X_1\cdots X_n}} & TX_1\cdots X_{n-1}\circ X_n^* \\
TX_1\cdots X_{n-1}\circ X_n^* & \xrightleftharpoons[k^{T,\text{act}}_{X_1\cdots X_n}]{k^{T,\text{act}}_{X_1\cdots X_n} e^{\delta\tilde{G}_{\text{act}}}} & TX_1\cdots X_{n-1}\circ X_n \\
TX_1\cdots X_{n-1} + X_n & \xrightleftharpoons[k^{T}_{X_1\cdots X_n} e^{-\delta\tilde{G}_{X_n}}]{k^{T}_{X_1\cdots X_n}} & TX_1\cdots X_{n-1}\circ X_n \\
TX_1\cdots X_{n-1}\circ X_n & \xrightleftharpoons[k^{T,\text{pol}}_{X_1\cdots X_n} e^{-\delta\tilde{G}_{\text{pol}}}]{k^{T,\text{pol}}_{X_1\cdots X_n} e^{-\delta\tilde{G}_{X_{n-1}}}} & TX_1\cdots X_n \\
TX_1\cdots X_L & \xrightleftharpoons[k^{T,u}_{X_1\cdots X_L}]{k^{T,u}_{X_1\cdots X_L} e^{-\delta\tilde{G}_{X_L}}} & T + X_1\cdots X_L \\
D + X_1 & \xrightleftharpoons[k^{D}_{X_1} e^{\delta\tilde{G}_f - \delta\tilde{G}_{X_1}}]{k^{D}_{X_1}} & DX_1 \\
DX_1\cdots X_{n-1} + X_n & \xrightleftharpoons[k^{D}_{X_1\cdots X_n} e^{\delta\tilde{G}_f - \delta\tilde{G}_{X_n} - \delta\tilde{G}_{\text{pol}}}]{k^{D}_{X_1\cdots X_n} e^{-\delta\tilde{G}_{X_{n-1}}}} & DX_1\cdots X_n \\
DX_1\cdots X_L & \xrightleftharpoons[k^{D,u}_{X_1\cdots X_L}]{k^{D,u}_{X_1\cdots X_L} e^{-\delta\tilde{G}_{X_L}}} & D + X_1\cdots X_L,
\end{array}
\tag{S22}
$$

where $n \leq L,\ X_i \in \{R, W\},\ X_i^* \in \{R^*, W^*\}$. As in the previous CRN, T represents the template, D the destructive catalyst, R the "right" monomers and W the "wrong" monomers. R^* and W^* are non-activated monomers. Dynamics is assumed to follow mass action kinetics, with rate constants given above and below the harpoons. The species, $TX_1\cdots X_{n-1}\circ X_n$ ($TX_1\cdots X_{n-1}\circ X_n^*$) represent a complex of polymer of length $n-1$ bound to the template as well as a (non-)activated monomer X_n (X_n^*) bound, but not yet polymerised into a single polymer of length n. $X_1\cdots X_L$ are the products. Both non-activated (X^*) and activated monomers (X) bind to the template or destructive catalyst with standard free energy $-\delta\tilde{G}_X$. If a non-activated monomer is bound to the template, it may be activated, with a free-energy change $\delta\tilde{G}_{\text{act}}$. If an activated monomer is bound to the template, it may be polymerised into the growing copolymer; the standard free-energy change of polymerisation is $-\delta\tilde{G}_{\text{pol}}$. The destructive catalyst has an additional free energy $\delta\tilde{G}_f$ per length driving the disassembly of polymers.

Once again, we linearise the system by assuming that monomers and catalysts are coupled to chemostats. In figure S3, we show part of the linearised CRN graph; this fragment should be inserted into figure S1 in place of the pathway ($\emptyset \to TR \to TRR \to RR$), with similar modifications to all other template-based pathways to RW, WR, and WW. Proofreading adds complexity to the graph in the form of additional loops, and we now explicitly consider a polymerization step independently from the binding to the template.

Monomers are now present in inactive (starred) and active forms, with $-\delta\tilde{G}_{\text{act}}$, representing the free-energy change of activation. We assume that each non-activated monomer $R^*,\ W^*$ is chemostatted at the same concentration as each activated monomer $R,\ W,\ c$. Dropping this assumption would only cause a shift to $\delta\tilde{G}_{\text{act}}$. As a result, $\delta\tilde{G}_f$ and $\delta\tilde{G}_{\text{act}}$ control the concentration bounds. We assume $\delta\tilde{G}_f,\ \delta\tilde{G}_{\text{act}} \geq 0$.

The most extreme paths we have been able to identify for this system have free-energy changes

$$
\delta\tilde{G}_U^* = \left\{ \begin{array}{c} \frac{L^2}{4} + \frac{L}{2} \\ \frac{(L+1)^2}{4} \end{array} \right\} \delta\tilde{G}_{\text{act}},
\tag{S23}
$$

and

$$\delta\tilde{G}_L^* = -\left\{ \begin{array}{c} \frac{L^2}{4} \\ \frac{L^2-1}{4} \end{array} \right\} \delta\tilde{G}_{\text{act}} - L\delta\tilde{G}_f, \tag{S24}$$

where the top value in each brace is for even L and the bottom value for odd L. Unlike the simple system in Note S5, the SAWs that exhibit $\delta\tilde{G}_U^*$ and $\delta\tilde{G}_L^*$ are not the intuitively simple pathways that go via the template and the destroyer, respectively. Instead, the pathways correspond to snaking through the CRN, alternately using both the template and destructive catalyst to first create a sequence, and then convert it into another sequence. We show these pathways for $L = 4$ in figure S4.

Since we have not formally proved that $\delta\tilde{G}_L^* = \delta\tilde{G}_L$ and $\delta\tilde{G}_U^* = \delta\tilde{G}_U$, $\Delta G \geq \Delta G^* = \delta\tilde{G}_L^* - \delta\tilde{G}_U^*$ and the "bounds" implied by $\Delta\tilde{G}^*$ are not strict bounds on system performance. Rather, they are bounds on the bounds; if we could identify $\Delta G > \Delta G^*$, then our theory would allow for even better information propagation. Nonetheless, we have not identified any sets of parameters that allow even the level of accuracy implied by ΔG^* to be achieved. The SAWs yielding $\delta\tilde{G}_U^*$ and $\delta\tilde{G}_L^*$ would not only be absurd as the dominant pathways in a real system, they actually cannot dominate production and degradation, even in principle. Since the pathways exhibiting $\delta\tilde{G}_U^*$ and $\delta\tilde{G}_L^*$ pass through other products as intermediates, scaling these pathways to be fast would necessarily result in sub-pathways to other products being fast.

Despite the above impossibility, the existence of these snaking pathways can provide some advantage, at least in principle. In figure S5, we show two attempts to find parameters that minimize entropy for a system with $L = 4$. In the first "naïve" scheme, plotted in blue, reactions contributing to assembly of $RRRR$ via the template or assembly of any other sequence via the destructive catalyst are assigned rates of 1 and all other rates are taken as $\sim k$. We show this scheme in figure S6. In the second "best guess" scheme, plotted in red, we make use of these snaking pathways. The reactions contributing to assembly of $RRRR$ via the template are still assigned rates of 1. However, for all other products, the longest snaking pathway which does not intersect with the pathway leading to $RRRR$ has rates assigned 1. We show this scheme in figure S7.

As $k \to 0$, the snaking pathways outperform the non-snaking pathways significantly, resulting in approximately half the entropy of the product distribution (figure S5). Notably, however, neither the best guess nor the naïve system converges on the minimal entropy of a system with a free-energy difference of $\Delta\tilde{G}^*$ between pathways as $k \to 0$; the best guess approaches $H = 1.8 \times 10^{-2}$ nats as compared to the bound at $H_{\min} = 1.9 \times 10^{-4}$ nats. Although the best guess system can outperform the naïve system in principle, when absolute rates can be chosen freely, rates may in practice be mechanistically constrained. As a result, this outperformance may not be achievable in a specific model. Indeed, at moderate k, the entropies of the naive system and the best guess converge.

Note S7 Example DNA strand displacement system that could maximise specificity in pseudo-equilibrium

Consider a molecular system in which two monomer species M_1 and M_2 can be catalytically activated into M_1^* and M_2^*. Such a setup could describe two enzymes that are activated by selective kinase-based phosphorylation, and deactivated by phosphatases. The system exhibits a maximally specific product ensemble in pseudo-equilibrium, analogous to the result obtained for catalytic assembly of polymers.

To see this behaviour, assume K and P catalyse activation with free-energy changes $\delta\tilde{G}_K$ and $\delta\tilde{G}_P$ along the respective pathways for both Monomer 1 and Monomer 2. Without loss of generality, assume $-\delta\tilde{G}_K > -\delta\tilde{G}_P$; informally, K tends to activate while P tends to deactivate. Then the steady state ratio of activated to deactivated Monomer 1 is given by

$$\frac{[M_1^*]}{[M_1]} = \frac{k_1^K + k_1^P}{k_{-1}^K + k_{-1}^P} = \exp(-\delta\tilde{G}_p)\frac{\frac{k_1^K}{k_1^P} + 1}{\frac{k_1^K}{k_1^P}\exp(\delta\tilde{G}_K - \delta\tilde{G}_p) + 1}, \tag{S25}$$

where k_1^K (k_{-1}^K) is the rate (absorbing the catalyst concentration) at which catalyst K activates (deactivates), and Monomer 1, k_1^P (k_{-1}^P) is the rate at which catalyst P activates (deactivates) Monomer 1, and we have used the detailed balance constraints $\frac{k_1^K}{k_{-1}^K} = \exp(-\delta\tilde{G}_K)$ and $\frac{k_1^P}{k_{-1}^P} = \exp(-\delta\tilde{G}_P)$. Equivalently,

$$\frac{[M_2^*]}{[M_2]} = \frac{k_2^K + k_2^P}{k_{-2}^K + k_{-2}^P} = \exp(-\delta\tilde{G}_p)\frac{\frac{k_2^K}{k_2^P} + 1}{\frac{k_2^K}{k_2^P}\exp(\delta\tilde{G}_K - \delta\tilde{G}_p) + 1}. \tag{S26}$$

Here, subscript "2" indicates that the rate is for Monomer 2's interconversion, and we have applied the same thermodynamic constraints as for Monomer 1.

Assume (without loss of generality) that we wish to maximise $[M_1^*]/[M_2^*]$ – the presence of the catalysts K and P is intended to activate M_1 and not M_2. Since $\exp(\delta\tilde{G}_K - \delta\tilde{G}_p) < 1$, we can immediately see that eq. S25 is maximised by allowing $k_1^K/k_1^P \to \infty$, in which case $\frac{[M_1^*]}{[M_1]} \to \exp(-\delta\tilde{G}_K)$. Similarly, eq. S26 is minimised by allowing $k_2^K/k_2^P \to 0$, in which case $\frac{[M_2^*]}{[M_2]} \to \exp(-\delta\tilde{G}_P)$. This optimal specificity for activation of Monomer 1 corresponds to Monomer 1 in a pseudo-equilibrium determined by the path coupled to K, and Monomer 2 in a pseudo-equilibrium determined by the path coupled to P. If we further assume, as in the main text, that inactive monomer concentrations are chemostatted at the same value, we obtain an optimal $\frac{[M_1^*]}{[M_2^*]} = \exp(-(\delta\tilde{G}_K - \tilde{G}_P)) = \exp(\Delta\tilde{G})$ as before.

A system directly analogous to the one described above can be designed using existing motifs from DNA nanotechnology[S1,S2], allowing pseudo-equilibrium systems to be studied in a concrete experimental setting. The mechanism, illustrated in figure S8, is based on 4-way strand exchange and allows fine tuning of the thermodynamic drive on each pathway in a way that is hard to achieve with, for example, ATP turnover.

Supplemental References

1. Mullor Ruiz, I. Development of a framework for designing nucleic acid-based, out-of-equilibrium catalytic reaction networks. PhD Thesis Imperial College London London, UK (2021).

2. Lankinen, A., Mullor Ruiz, I., and Ouldridge, T.E. (2020). Implementing Non-Equilibrium Networks with Active Circuits of Duplex Catalysts. In C. Geary, and M.J. Patitz, eds. 26th International Conference on DNA Computing and Molecular Programming (DNA 26). Schloss Dagstuhl – Leibniz-Zentrum für Informatik pp. 7:1–7:25.

3. Nam, K.M., Martinez-Corral, R., and Gunawardena, J. (2022). The linear framework: using graph theory to reveal the algebra and thermodynamics of biomolecular systems. Interface Focus *12*, 20220013.

4. Robinson, S.M. (1970). A short proof of Cramer's rule. Mathematics Magazine *43*, 94–95.

5. Maes, C., and Netočný, K. (2013). Heat bounds and the blowtorch theorem. Ann. Henri Poincaré *14*, 1193–1202.

6. Sáez, M., Feliu, E., and Wiuf, C. (2019). Linear elimination in chemical reaction networks. In J.L. García Guirao, J.A. Murillo Hernández, and F. Periago Esparza, eds. Recent Advances in Differential Equations and Applications pp. 177–193.. Springer International Publishing pp. 177–193.

7. Çetiner, U., and Gunawardena, J. (2022). Reformulating nonequilibrium steady states and generalized hopfield discrimination. Phys. Rev. E *106*, 064128.

8. Arunachalam, E., and Lin, M.M. (2025). Information Gain Limit of Biomolecular Computation. Phys. Rev. Lett. *134*, 148401.

9. Ouldridge, T.E. (2018). The importance of thermodynamics for molecular systems, and the importance of molecular systems for thermodynamics. Nat. Comput. *17*, 3–29.

10. Cover, T.M., and Thomas, J.A. (2006). Elements of information theory. Wiley-Interscience.

11. Polettini, M., and Esposito, M. (2014). Irreversible thermodynamics of open chemical networks. i. emergent cycles and broken conservation laws. J. Chem. Phys. *141*, 024117.

12. Qureshi, B., Juritz, J., Poulton, J.M., Beersing-Vasquez, A., and Ouldridge, T.E. (2023). A universal method for analyzing copolymer growth. J. Chem. Phys. *158*, 104906.

13. Poulton, J.M., Ten Wolde, P.R., and Ouldridge, T.E. (2019). Nonequilibrium correlations in minimal dynamical models of polymer copying. Proc. Natl. Acad. Sci. U.S.A. *116*, 1946–1951.

14. Hopfield, J.J. (1974). Kinetic proofreading: a new mechanism for reducing errors in biosynthetic processes requiring high specificity. Proc. Natl. Acad. Sci. U.S.A. *71*, 4135–4139.

15. Ninio, J. (1975). Kinetic amplification of enzyme discrimination. Biochimie *57*, 587–595.

16. Bennett, C.H. (1979). Dissipation-error tradeoff in proofreading. BioSystems *11*, 85–91.

17. Mallory, J.D., Igoshin, O.A., and Kolomeisky, A.B. (2020). Do we understand the mechanisms used by biological systems to correct their errors? J. Phys. Chem. B *124*, 9289–9296.
