# Supplementary material for: Thermodynamic limits in far-from-equilibrium molecular templating networks
Source: Newton. 2026 Jan 5;2(1):None. doi: 10.1016/j.newton.2025.100302 (PMC12769091; doi:10.1016/j.newton.2025.100302)
Supplement: Document S2. Article plus supplemental information [file mmc2.pdf]

Newton

Article

Thermodynamic limits in far-from-equilibrium molecular templating networks

Graphical abstract

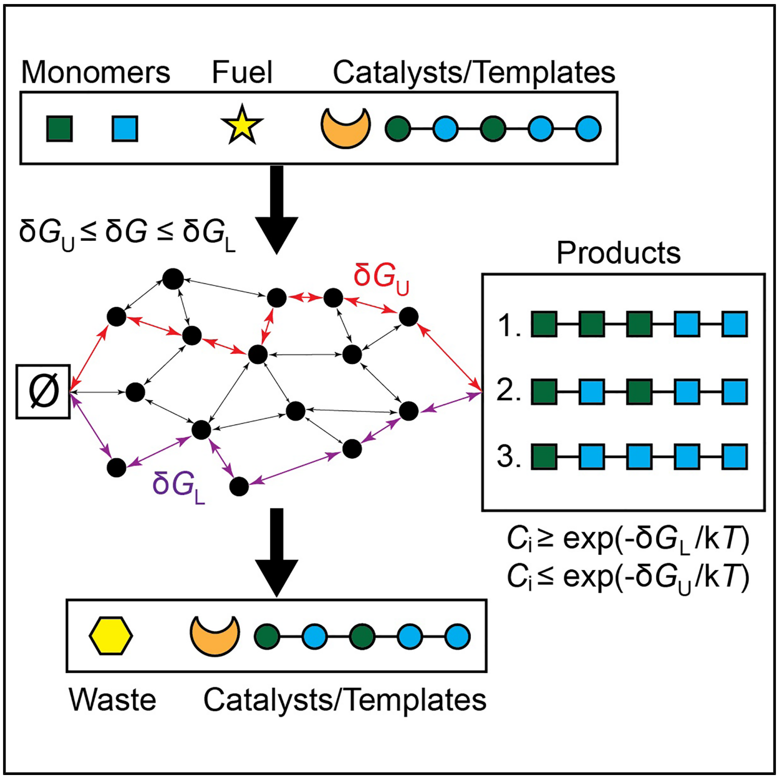

Authors

Benjamin Qureshi, Jenny M. Poulton, Thomas E. Ouldridge

Correspondence

t.ouldridge@imperial.ac.uk

In brief

In cellular systems, nucleic acid templates catalyze the formation of protein and RNA molecules. Qureshi et al. analyze the behavior of catalytic molecular templating networks and bound the accuracy of such templating networks via functions of the maximal difference in free-energy changes between possible assembly pathways. Surprisingly, systems that operate at the bounds exist in a pseudo-equilibrium, balancing forward and backward transitions, unlike the behavior observed in biology.

Highlights

- Derivation of fundamental bounds on the accuracy of molecular templating networks
- Identification of unexpected pseudo-equilibrium behavior at these bounds
- Resolution of the paradox surrounding the necessary costs of accurate templating

Qureshi et al., 2026, Newton 2, 100302
January 5, 2026 © 2025 The Author(s). Published by Elsevier Inc.
https://doi.org/10.1016/j.newton.2025.100302

CellPress

Newton

CellPress
OPEN ACCESS

Article

Thermodynamic limits in far-from-equilibrium molecular templating networks

Benjamin Qureshi,[1,2] Jenny M. Poulton,[3] and Thomas E. Ouldridge[1,2,4,*]
[1]Department of Bioengineering, Imperial College London, London SW7 2AZ, UK
[2]Centre for Engineering Biology, Imperial College London, London SW7 2AZ, UK
[3]Department of Physics and Astronomy, University of Sheffield, Sheffield S3 7RH, UK
[4]Lead contact
*Correspondence: t.ouldridge@imperial.ac.uk
https://doi.org/10.1016/j.newton.2025.100302

ACCESSIBLE OVERVIEW Cells assemble complex molecules such as proteins using template polymers with information-carrying sequences. These sequences are used to direct the ordered assembly of a set of molecular building blocks to form exactly the right product, such as a specific protein, on demand. A crucial feature of this process is that the templates act catalytically, since they accelerate the assembly of a specific product without being consumed in the process. A single template can then be used to direct the assembly of many copies of a product. It has long been suspected that this transfer of information from catalytic templates to products must be associated with a minimal energy cost, in the same way that computation has lower bounds on energy input. To explore this hypothesis further, this work considers arbitrarily complex templating networks involving many assembly and disassembly processes. We show that the accuracy with which a specific set of products can be maintained is, indeed, constrained by energetic properties of the underlying network. Surprisingly, the operation of the most efficient templating networks is completely different from the behavior observed in nature, where molecules such as proteins are overwhelmingly assembled by templates and degraded in a distinct, template-free fashion. Instead, the most efficient approach, which both achieves the highest accuracy and has a negligible energetic cost, is to disassemble products via a reversal of the pathway by which they were formed. This observation raises interesting questions about why nature does not operate in this way and whether synthetic templating systems can be built that exploit this approach.

SUMMARY

Cells maintain a highly specific, far-from-equilibrium population of RNA and protein molecules. They do so via complex reaction networks in which templates catalyze the assembly of desired products. We show that information transmission from templates to products in such networks is bounded by functions of the maximal difference in free-energy changes between assembly pathways. Surprisingly, putative systems operating at the bounds do not have a high net flux around the network, as is typical in far-from-equilibrium systems and observed in biology. Instead, the upper bound on accuracy for a given network structure is achieved in "pseudo-equilibrium." Here, each product is produced and degraded by time-reversed trajectories along a single (product-specific) pathway with negligible entropy production; product yields are determined by the free-energy changes along those pathways. The limit imposed by these free-energy changes induces a thermodynamic constraint on accuracy, even if a single templating process is arbitrarily kinetically selective.

INTRODUCTION

Cellular mRNA and DNA molecules selectively catalyze the formation of many distinct protein and mRNA sequences from a small set of monomer building blocks.[1,2] Both proteins and RNA are also actively degraded.[3] The underlying processes are complex, with many pathways to assembly and disassembly and non-trivial motifs—such as kinetic proofreading[4,5]—within those pathways. The net result is a distribution of protein and RNA sequences in the cell, biased toward template-specified targets; sharply peaked, or *accurate*, distributions are essential for function.

The theory of such systems has generally focused on isolated templating events[6–19] rather than full networks. Previous works[2,20] have hypothesized that such networks would require a minimal

© 2025 The Author(s). Published by Elsevier Inc.
This is an open access article under the CC BY license (http://creativecommons.org/licenses/by/4.0/).

entropy production per output polymer, determined by the sequence information transmitted from template to products. Recently, Genthon et al.[21] modeled a network with competing templating and spontaneous degradation pathways, treating both as a one-step process with an imposed kinetic selectivity. They observed a phase transition to a high-accuracy regime with a large cyclic flux of templated production and spontaneous degradation, with a minimal fuel turnover per product made commensurate with the accuracy of the product ensemble, apparently confirming the predictions of Ouldridge and ten Wolde[2] and Bennett.[20] Relatedly, others have shown that the responsivity of steady states of complex networks to perturbations depends on the thermodynamic driving within the system,[22,23] without relating the results to the concentrations of products in templating systems.

Given the simplicity of the system in Genthon et al.,[21] the thermodynamics of arbitrarily complex molecular templating networks is underexplored. Moreover, previous works[2,20,21] suggest a paradox. Entropy production is related to the relative rates of time-reversed trajectories[24] rather than the relative rates of distinct processes. Although biochemistry may practically limit relative templating rates for matching and non-matching sequences, thermodynamics places no limit on this kinetic discrimination *in principle*. Why then, is accuracy associated with a minimal thermodynamic cost?[25]

Here, we first show that functions of a simple quantity—$\Delta\tilde{G}$, the $k_B T$-normalized difference between the maximal and minimal free-energy changes along assembly pathways—bounds the accuracy of arbitrarily complex molecular templating networks. We then explore these bounds for a system in which M possible products can be formed. For $M \rightarrow \infty$, it is possible to maintain a steady-state ensemble with only a single product type, provided $\frac{\Delta\tilde{G}}{\ln M} > 1$. By contrast, a single product necessarily has zero weight within the ensemble for $\frac{\Delta\tilde{G}}{\ln M} < 1$. These results appear to confirm previous hypotheses[2,20] and generalize the results from a recently published work.[21] However, the bounds are more restrictive at finite M, and for $M \rightarrow \infty$, one can surprisingly maintain an ensemble dominated by a vanishingly small fraction of the possible products for any $\Delta\tilde{G} \gg 1$, even if $\Delta\tilde{G} \ll \ln M$, suggesting that a set of templates can be copied with high accuracy when a single one cannot.

Most significantly, however, systems that approach the $\Delta\tilde{G}$-dependent bounds do not have large, entropy-producing cyclic fluxes, as is common in far-from-equilibrium systems, anticipated in the case of templating networks,[2,20,21] and observed in biology. Instead, each product overwhelmingly couples to a single pathway, balancing forward and backward transitions and exhibiting a yield determined by that pathway's free-energy change. These bound-saturating "pseudo-equilibria" have negligible entropy production per assembly event and resolve the apparent paradox of the minimal cost of templating.

RESULTS

A modeling framework for arbitrarily complex molecular templating networks

We consider a broad class of networks, including that of Genthon et al.[21] as a special case. As shown in Figure 1A, we consider the assembly of a set of monomer species (e.g., amino acids) into products. The "alphabet size" of this set of monomer species (2 in Figure 1) is arbitrary. These monomers can form M possible products. For clarity, we take these products to be linear polymers, as in transcription and translation, although the underlying theory does not require this assumption. The products may also be disassembled into monomers. These assembly and disassembly pathways are coupled to catalysts (e.g., ribosomes and proteases), some of which are sequence-bearing templates (e.g., mRNA), and can also involve turnover of fuel molecules into waste (e.g., ATP into ADP). In any given instance, we expect only a small fraction of the possible template species to be present.

To simplify analysis, we assume a symmetric model in which all products are polymers of a fixed length $L \sim \ln M$. Monomers are equivalent: they have the same free energy, and any sequence of monomers of the right length constitutes a product. As is common,[2,14,21,25] we further assume the equivalence of the products, in the sense that they all contain the same number of monomers and have the same normalized standard free energy of formation, $\tilde{G}_0$, which would be expected to scale linearly with $L \sim \ln M$ regardless of the templates present. This choice is the simplest one consistent with the fundamental fact that catalytic templates cannot change $\tilde{G}_0$.[2,14] We will revisit these symmetry assumptions in the discussion.

We allow for an arbitrarily complex, thermodynamically self-consistent chemical reaction network (CRN) between the monomers and the products. A subset of reactions in a plausible network, forming two branched assembly/disassembly pathways through intermediates (any molecular complex excluding isolated monomers, products, catalyst, waste, or fuel), is given in Figure 1B. This example includes a trimer template that catalyzes the formation of a product with a matching sequence—although it is also possible to form the wrong sequence with a mismatched monomer—and a non-specific enzyme that consumes fuel to systematically degrade these products.

Aside from the existence of a reverse reaction for each forward reaction, which is required for thermodynamic self-consistency,[24,26] we impose only two restrictions on the reaction network. First, no reactions occur between more than one product and/or intermediate. Second, due to our symmetry assumptions, if a set of chemical reactions whose net effect is to assemble a given product exists, a corresponding set of reactions involving the same fuels and catalysts exists for all products. For example, in Figure 1B, both the product that matches the template and one with a mismatch can form via the same template. Note that although these alternate reactions exist, they may be much slower due to selective catalysis by the templates.

We further assume that monomers are held at the same constant concentration (chemostatted) by the environment. Catalysts and fuels are also assumed to be chemostatted but at arbitrary concentrations. This approximation reflects the fact that in cells, monomers and fuel are generally abundant and their concentration maintained. However, the abundance of free mRNA, polymerase, or ribosome catalysts is more likely to be reduced by sequestration; we will therefore revisit the assumption that catalysts are chemostatted in the discussion.

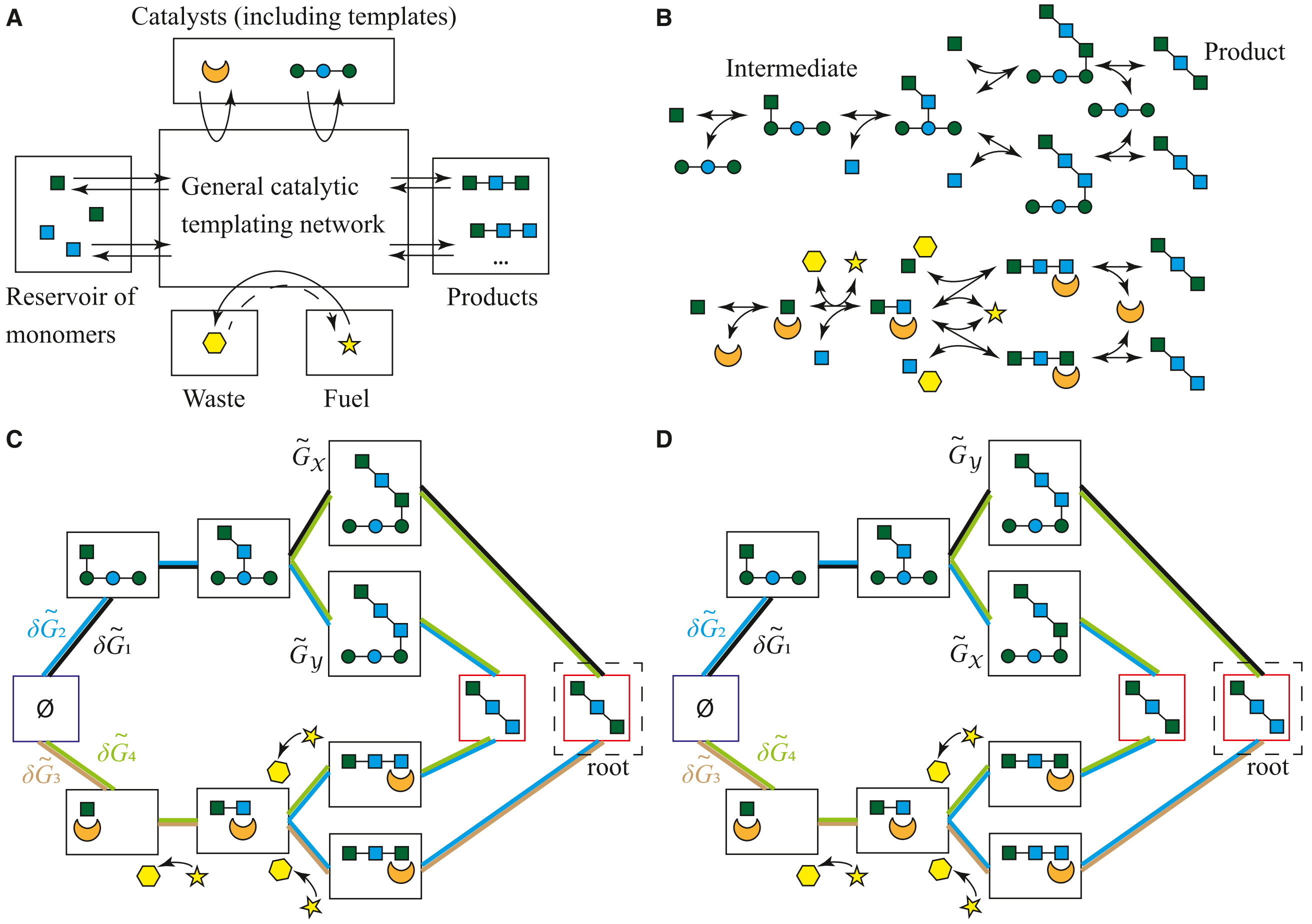

Figure 1. Modeling framework

(A) Polymers are produced via catalyzed addition of monomers in an arbitrarily complex reaction network, driven by fuel consumption.

(B) A subset of reactions in an example system, showing two branched pathways for production/degradation of two products, one involving a template catalyst and one involving another catalyst and fuel turnover. Equivalent sets of reactions, using the same fuels and catalysts (but different monomers), can produce both products shown.

(C) Representation of the reactions in (B) as a graph, with nodes (rectangles), including a null complex Ø; intermediate species (black squares); and products (red squares). Each edge is a reversible transition, and each distinct pathway from Ø to the product has an associated normalized free-energy change $\delta\tilde{G}_i$ (illustrated here for the "root" product).

(D) We take an alternative product as the root of the graph; the network connecting it to the null state is topologically identical to (C). The equivalent pathways for each product have the same overall $\delta\tilde{G}_i$, but intermediate free energies (and transition rates) can vary: for example, $\tilde{G}_{\mathcal{X}} \neq \tilde{G}_{\mathcal{Y}}$ in general.

Representation of the templating network as a linear graph

We analyze these systems using deterministic CRNs[27,28] obeying mass-action kinetics. CRNs consist of a set of species, a set of complexes (collections of species), and a set of reactions in which a complex $\mathcal{X}$ is converted into a complex $\mathcal{Y}$ at a rate given by a rate constant $k_{\mathcal{X}\to\mathcal{Y}}$ multiplied by the product of concentrations of the species in $\mathcal{X}$.

Under our assumptions, only the products and intermediates are species in the CRN, with monomer, fuel, and free-catalyst concentrations acting as parameters that are absorbed into rate constants. Each complex then either contains a single species or is null (Ø); transitions from Ø correspond to the first association between monomers and catalysts. The CRNs are therefore linear and can be drawn as a graph; Figure 1C shows the graph formed by the possible subset of reactions in Figure 1B, with nodes as complexes and edges as reactions. The graph for the full network is connected since all complexes can be reached from Ø. This graph defines a set of self-avoiding walks (SAWs) from Ø to each product; each SAW and its inverse define a "pathway." Pathways are illustrated by colored edges in Figure 1C. Our symmetry assumptions mean that all products are connected to ∅ by topologically equivalent graphs—this concept is illustrated in Figure 1D and formally defined in Note S1.

Imposing self-consistent thermodynamics on the model

For thermodynamic self-consistency, we require that forward ($\mathcal{X}\to\mathcal{Y}$) and reverse ($\mathcal{Y}\to\mathcal{X}$) transitions obey local detailed balance[24]: $\delta\tilde{G}_{\mathcal{X}\to\mathcal{Y}} = -\ln(k_{\mathcal{X}\to\mathcal{Y}}/k_{\mathcal{Y}\to\mathcal{X}})$, where $\delta\tilde{G}_{\mathcal{X}\to\mathcal{Y}}$ is the k_BT-normalized standard free-energy change of the reaction.[26]

Here, $\delta\tilde{G}_{\mathcal{X}\to\mathcal{Y}} = \tilde{G}_{\mathcal{Y}} - \tilde{G}_{\mathcal{X}} + \sum_i \tilde{\mu}_i \delta N_i^{\mathcal{X}\to\mathcal{Y}}$, with $\tilde{G}_{\mathcal{X}} - \tilde{G}_{\mathcal{Y}}$ representing the free-energy change of the non-chemostatted species and $\tilde{\mu}_i \delta N_i^{\mathcal{X}\to\mathcal{Y}}$ representing the free-energy change due to the production of $\delta N_i^{\mathcal{X}\to\mathcal{Y}}$ molecules of type i, chemostatted at a $k_B T$-normalized chemical potential $\tilde{\mu}_i$. Each edge corresponds to a fixed $\delta N_i^{\mathcal{X}\to\mathcal{Y}}$, so $\delta\tilde{G}_{\mathcal{X}\to\mathcal{Y}}$ is well defined. However, two SAWs, S and S', connecting Ø and a given product, such as the pink and brown SAWs in Figure 1C, can consume different amounts of fuel, allowing $\delta\tilde{G}_S \neq \delta\tilde{G}_{S'}$, where $\delta\tilde{G}_S = \sum_{e\in S}\delta\tilde{G}_e$. The framework thus allows for arbitrarily complex non-equilibrium networks, incorporating separate production and degradation pathways and kinetic proofreading cycles.

Recalling that $\tilde{G}_0$ is the standard free energy of product formation, the total free-energy change along a SAW is then $\delta\tilde{G}_S = \tilde{G}_0 + \sum_{e\in S}\sum_i \tilde{\mu}_i \delta N_i^e$. Since, based on our symmetry assumptions, all products are connected to Ø by topologically equivalent pathways involving the same fuel consumption, the set of free-energy changes associated with the formation of each product is also equivalent (this equivalence is illustrated in Figures 1C and 1D). By contrast, we allow for arbitrary intermediate free energies; for example, we expect $\tilde{G}_{\mathcal{X}} \neq \tilde{G}_{\mathcal{Y}}$ in Figures 1C and 1D due to the specificity of interactions with the template, and transition rates are unconstrained except by $\delta\tilde{G}_{\mathcal{X}\to\mathcal{Y}} = -\ln(k_{\mathcal{X}\to\mathcal{Y}}/k_{\mathcal{Y}\to\mathcal{X}})$. Certain products can then form faster via kinetically selective templating.

We first show that, under these assumptions, properties of the steady-state distribution of products are bounded by functions of $\Delta\tilde{G}$, the difference between the maximum and minimum $\delta\tilde{G}_S$, for any self-consistent choice of rates. We will then explore the nature of these bounds.

Bounds on the product ensemble

We consider the steady-state concentration of products (the "product ensemble"); our results also apply to the expected steady-state concentrations of a stochastic realization of the CRN.[29] For linear, connected CRNs with a null complex, the steady-state concentration of any species is bound by the free-energy changes along the SAWs connecting that product state to Ø.[22,30–33] Defining $\delta\tilde{G}_L^{Z_i} = \max_S \delta\tilde{G}_S^{Z_i}$ and $\delta\tilde{G}_U^{Z_i} = \min_S \delta\tilde{G}_S^{Z_i}$, where the optimization is performed over all SAWs that lead to Z_i from θ, then all concentrations obey the fundamental constraint $e^{-\delta\tilde{G}_L^{Z_i}} \leq c_{Z_i} \leq e^{-\delta\tilde{G}_U^{Z_i}}$ (see Note S2). For our networks, all products are connected to Ø by pathways with the same set of free-energy changes. All products therefore have the same template-independent upper and lower bounds $(e^{-\delta\tilde{G}_U}, e^{-\delta\tilde{G}_L})$.

To see how these constraints manifest, we define the product distribution $\mathbb{P}(Z_i)$ for products Z_i, $i = 1, \cdots M$:

$$\mathbb{P}(Z_i) = p_i = \frac{c_{Z_i}}{\sum_{j=1}^{M} c_{Z_j}} = \frac{c_{Z_i}}{c_T}. \quad \text{(Equation 1)}$$

Here, c_{Z_i} is the concentration of product Z_i and $c_T = \sum_{j=1}^{M} c_{Z_j}$ is the total concentration. We consider two metrics for the deviation from a uniform equilibrium ensemble. First, the single-product specificity $p_{\max} = \max_i p_i$, which is a natural metric for the accuracy with which a single isolated template can influence the product ensemble. Second, we consider the (Shannon) entropy[34] of the distribution

$$H[p_i] = -\sum_{i=1}^{M} p_i \ln p_i = \ln c_T - \frac{1}{c_T}\sum_{i=1}^{M} c_{Z_i} \ln c_{Z_i}. \quad \text{(Equation 2)}$$

We consider this metric because $\ln M - H[p_i]$ is the channel capacity of the network if it is treated as an information channel from templates to products (see Note S4). Furthermore, this metric is better suited to systems (such as cells) in which a set of templates catalyzes the assembly of a number of distinct products in parallel. We describe optimizing for single-product specificity or entropy as specificity maximization or entropy minimization, respectively.

Given $e^{-\delta\tilde{G}_L} \leq c_{Z_i} \leq e^{-\delta\tilde{G}_U}$, specificity maximization at fixed $\Delta\tilde{G} = \delta\tilde{G}_L - \delta\tilde{G}_U$ is saturated by an ensemble with one species at $p_{\max}$ and all others at p_{low}, with

$$p_{\max} = \left(1 + (M-1)e^{-\Delta\tilde{G}}\right)^{-1}, p_{\text{low}} = e^{-\Delta\tilde{G}} p_{\max}. \quad \text{(Equation 3)}$$

This $p_{\max}(\Delta\tilde{G})$ thus defines a bound. Entropy minimization yields $H[p_i] \geq H(m)$, with

$$H(m) = \frac{(M-m)\Delta\tilde{G}e^{-\Delta\tilde{G}}}{m + (M-m)e^{-\Delta\tilde{G}}} + \ln\left(m + (M-m)e^{-\Delta\tilde{G}}\right). \quad \text{(Equation 4)}$$

$H(m)$ corresponds to m species at the upper bound, $c_U = \exp(-\delta\tilde{G}_U)$, and $M - m$ species at the lower bound, $c_L = \exp(-\delta\tilde{G}_L)$. Minimizing Equation 4 with respect to integer m yields the lower bound on entropy, $H_{\min}(\Delta\tilde{G})$. $H_{\min}$ and the minimizing $m = m_{\min}$ and a proof of Equation 4 are given in Note S3. For illustrative purposes, in this manuscript, we use the approximation in Equation S17, which treats $m_{\min}$ as continuous when $m_{\min} > 1$ (a slightly weaker bound).

The bounds in the presence of kinetic proofreading

Surprisingly, internal cycles in the reaction network, a requirement for celebrated kinetic proofreading motifs,[4,5,10] do not directly feature in the bounds derived, since SAWs cannot contain cycles by definition. Adding a proofreading loop to a process may affect $\Delta\tilde{G}$ by providing a new pathway with a maximal or minimal free-energy change, but the SAWs identified would always be loop free and correspond to a fixed free-energy change for product formation. The possibility of repeatedly undergoing a single dissipative cycle in an actual dynamic trajectory, consuming an arbitrary amount of molecular fuel, does not translate into an arbitrary $\delta\tilde{G}$ along a pathway. However, the existence of loops within the network may still help the system to achieve a better product distribution than would otherwise occur if practical constraints on transition rates prevent the system from reaching the $\delta\tilde{G}$-dependent limits.

Specificity maximization and entropy minimization

In Figure 2A, we plot the bound on $p_{\max}$ against $\Delta\tilde{G}/\ln M$ for various M ($\ln M$ is proportional to the length of

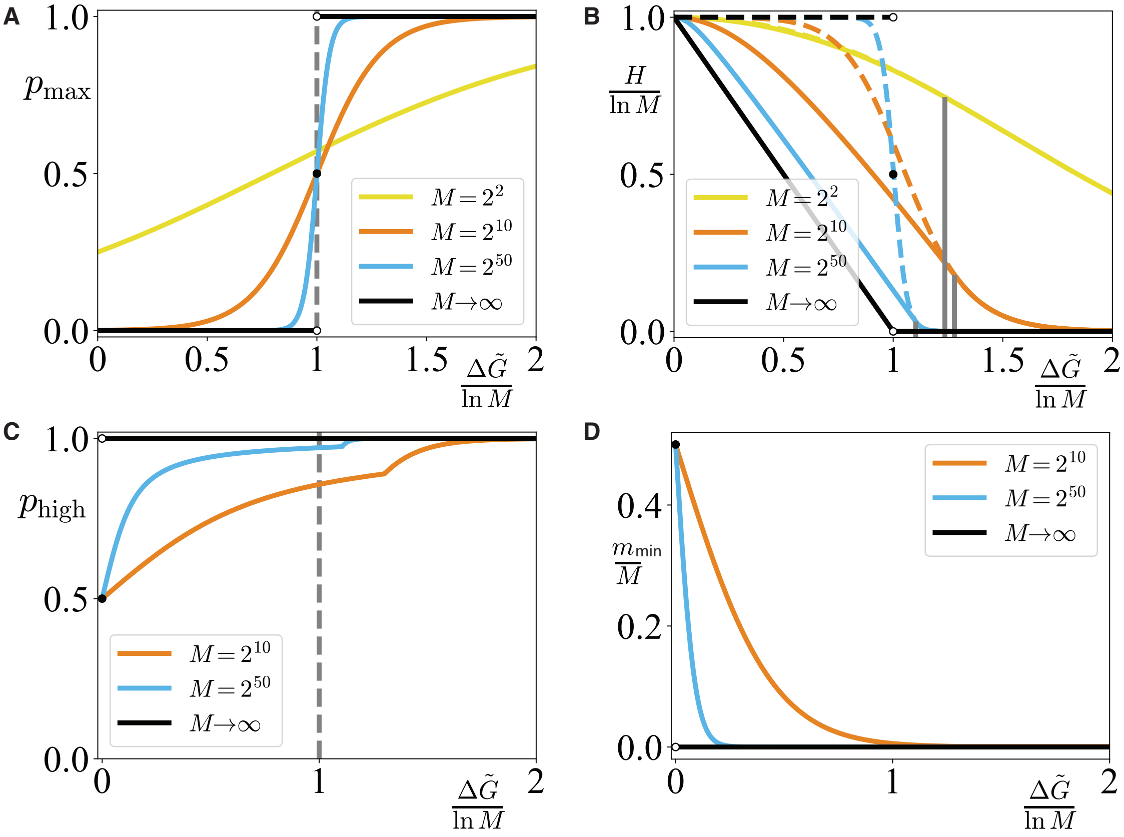

Figure 2. Behavior of single-product specificity and entropy bounds

(A) The bound on single-product specificity p_{max} as a function of $\Delta\tilde{G}/\ln M$, for various M.

(B) The bound on entropy H_{min} (solid lines) and the entropy of the distribution of maximal single-product specificity (dashed lines) as a function of $\Delta\tilde{G}$ for various M (for $M = 2^2$, the lines nearly overlap). We scale both quantities by $\ln M$ so that they are defined per unit length. The dashed line is discontinuous at $\Delta\tilde{G} = \ln M$ for $M\to\infty$. The vertical (gray) lines show $\Delta\tilde{G} = \ln M + \ln\ln M$, below which the curves do not overlap.

(C) The probability of selecting a product at the high concentration limit in the entropy-minimized ensemble, p_{high}, as a function of $\Delta\tilde{G}/\ln M$, for various M.

(D) The fraction of products at the high concentration limit in an entropy-minimized ensemble, m_{min}/M, as a function of $\Delta\tilde{G}/\ln M$, for various M.

(C) and (D) together show that a small fraction of products can dominate the ensemble, even below $\Delta\tilde{G}/\ln M = 1$.

a copolymer, and $\Delta\tilde{G}/\ln M$ therefore measures the free-energy difference per unit length). p_{max} undergoes a transition from M^{-1} to 1 as $\Delta\tilde{G}/\ln M$ is increased, centered on $\Delta\tilde{G}/\ln M = 1$. As $M\to\infty$, the sigmoid becomes a phase transition.[21] We note that this phase transition is qualitatively distinct from a traditional equilibrium order-disorder transition. Since all products have the same $\tilde{G}_0$, there is no energetic benefit to the "ordered" state with a single product. The system is necessarily enormously far from equilibrium, and any specificity is fundamentally a kinetic phenomenon.

Surprisingly, specificity maximization and entropy minimization are not equivalent. In Figure 2B, we plot the bound on H_{min} and the entropy of the maximum specificity distribution against $\Delta\tilde{G}/\ln M$. The two curves overlap for $\Delta\tilde{G} > \ln M + \ln\ln M + \mathcal{O}\left(\frac{\ln\ln M}{\ln M}\right)$, for which values of $\delta\tilde{G}$ $m = 1$ minimizes Equation 4 and the lowest entropy state has a single product at a concentration $c_U = e^{-\delta\tilde{G}_U}$ and all others at $c_L = e^{-\delta\tilde{G}_L}$. For smaller $\Delta\tilde{G}$, the two differ drastically. H_{min} is obtained for distributions with $m_{min} > 1$ products at c_U. This unexpected behavior arises because having $m > 1$ products at $e^{-\delta\tilde{G}_U}$ increases c_T and thus suppresses the probabilities of other species.

Figure 2C shows the total probability p_{high} of selecting a high-concentration product from the entropy-minimizing distribution, and Figure 2D shows the fraction m_{min}/M of products at c_U in that ensemble. Although m_{min} increases and p_{high} decreases as $\Delta\tilde{G}\to 0$, for large M, m_{min}/M tends to zero while maintaining $p_{high} > 0.5$ even for $\Delta\tilde{G} \ll \ln M$, well inside the region where single-product specificity is impossible. As $M\to\infty$ at fixed $\Delta\tilde{G}/\ln M$, a vanishingly small proportion of the total number of possible products ($m_{min}/M\to 0$) can dominate the ensemble ($p_{high}\to 1$) even when $\Delta\tilde{G} \ll \ln M$. This result has consequences for systems in which a set of unrelated templates acts in parallel, as in cells. Although an ensemble of products dominated by copies of a single template is impossible below $\Delta\tilde{G}/\ln M = 1$, an ensemble in which most products are accurate copies of a relatively small set of unrelated templates is possible.

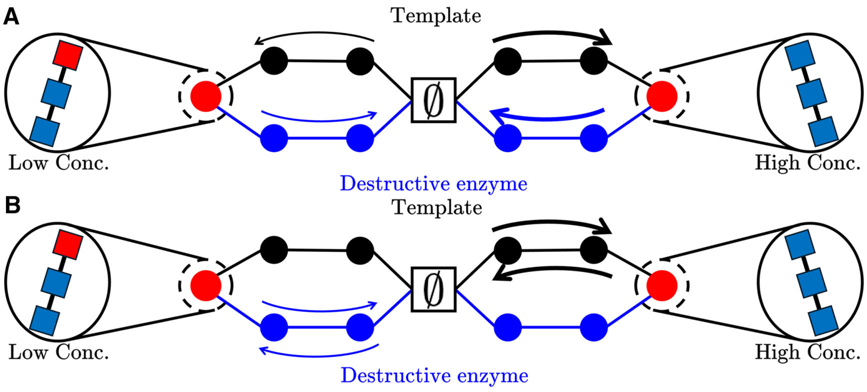

Figure 3. Contrasting a non-equilibrium steady state with a high cyclic flux and a non-equilibrium steady state in pseudo-equilibrium

(A) A steady state with a high cycle flux.

(B) A steady state in pseudo-equilibrium. In each case, the dominant production and degradation trajetories are shown.

Physical meaning of the bounds

The phase transition in $p_{\max}$ at $\Delta\tilde{G}/\ln M = 1$ for $M \to \infty$ apparently generalizes the observations of Genthon et al.[21] to arbitrarily complex networks and supports the hypotheses of Ouldridge and ten Wolde[2] and Bennett,[20] suggesting a minimal cost of accurately copying a single template. Notably, however, Figures 2C and 2D are hard to interpret in the context of a minimal cost of accuracy, as they suggest that it is possible to reduce the relative abundance of erroneous products at low values of $\Delta\tilde{G}/\ln M$ simply by having a larger (but still relatively small) set of unrelated templates. Simultaneously, significantly larger values of $\Delta\tilde{G}/\ln M$ are required to give perfect accuracy for finite M (see Figure 2A). Consider the implications for the charging of a single tRNA with an amino acid, an example of templated dimerization. There are approximately $M = 400$ combinations of codons and amino acids, having grouped redundant codons. For a single charged tRNA to dominate the ensemble with an error rate of 10^{-5}, one would need a $\Delta\tilde{G}$ of around 17 k_BT or 1 ATP at 37°C, substantially higher than $k_BT \ln M = k_BT \ln 400 \approx 6\ k_BT$, the value implied by the large M limit.

Most importantly, a putative system that approaches the bounds on $p_{\max}$ and $H[p_i]$ behaves unexpectedly (Figure 3). $c_{Z_X} = \exp(-\delta\tilde{G}_U)$ can only be reached if Z_X is produced and degraded solely by the pathway with free-energy change $\delta\tilde{G}_U$; similarly, $c_{Z_Y} = \exp(-\delta\tilde{G}_L)$ requires Z_Y to be produced and degraded solely by the pathway with free-energy change $\delta\tilde{G}_L$. Z_X and Z_Y are then in pseudo-equilibria, with their yields equal to the equilibrium yield of the relevant pathway.

These pseudo-equilibria, whether or not they are practically achievable, limit all possible non-equilibrium templating systems for a given $\Delta\tilde{G}$. Indeed, any non-zero net flux necessarily *reduces* accuracy relative to this limit. Consider two products X and Y and assume $c_X > c_Y$. Let $\Gamma_X(\delta\tilde{G})$ be the observed ratio of production to degradation trajectories of X along a pathway with free-energy change $\delta\tilde{G}$; deviations of Γ from unity imply a net flux. At a steady state, thermodynamic self-consistency implies $\Gamma_X(\delta\tilde{G}) = \exp(-\delta\tilde{G})/c_X$. Thus,

$$\ln(c_X / c_Y) = \Delta\tilde{G} + \ln\left(\frac{\Gamma_Y(\delta\tilde{G}_L)}{\Gamma_X(\delta\tilde{G}_U)}\right). \qquad \text{(Equation 5)}$$

Since $\delta\tilde{G}_U$ corresponds to the most favorable production pathway, $\Gamma_X(\delta\tilde{G}_U) \geq 1$. Conversely, $\Gamma_Y(\delta\tilde{G}_L) \leq 1$. We see that a systematic flux to produce X along the most favorable production pathway or to degrade Y along the least favorable production pathway necessarily reduces the concentration ratio beneath that required to reach the bounds ($c_X = c_Y \exp(\Delta\tilde{G})$ if $c_X > c_Y$). Moreover, Equation 5 shows that the degree to which a system deviates from pseudo-equilibrium behavior, as measured by the Γ ratios, quantitatively dictates the relative concentrations of species and therefore the accuracy. Both the bound itself and the nature of systems that reach the bound are, unusually, directly relevant to the accuracy of systems operating far from the bound, such as those in nature.

Equally surprisingly, while operating at this pseudo-equilibrium limit requires a certain $\Delta\tilde{G}$ for accuracy, this "cost" is not an entropy production per product made, as it would be for a system with a high cyclic flux. If typical production and degradation events are truly time-reversed processes, the system will have zero net fuel consumption; all fuel consumed in production would be regenerated during destruction, without the need for a costly external recycling process. Entropy generated per production or degradation event is thus zero in a steady state—at variance with the expectations of Ouldridge and ten Wolde,[2] Bennett,[20] and Genthon et al.[21]

Fundamentally, these observations all follow because templating's limiting "cost" is not needed to ensure the kinetic selectivity of a template in isolation—the ratio of catalytic rates is unconstrained by the second law. Rather, $\Delta\tilde{G}$-dependent bounds arise because steady-state yields cannot vary by more than the pseudo-equilibria arising from maximally different pathways. This observation explains why accurate templating systems have a minimal cost despite apparently only needing to distinguish between processes that are not time-reversed trajectories. It also explains why multiple templates can apparently be copied with more accuracy than a single one. If accuracy were constrained by a limit on template selectivity, adding more (unrelated) templates to the system would increase the total concentration of both desired and undesired products. In fact, however, at maximal accuracy, the undesired products equilibrate with the pathway of maximal $\delta\tilde{G}$, and so adding new pathways with low $\delta\tilde{G}$ does not increase their minimal concentration.

Pseudo-equilibrium limits only apply in the steady state, and no lower bound on $\Delta\tilde{G}$ is required for accuracy outside of the steady state. Consider a system with two products: $\varnothing \underset{k_X}{\overset{k_X}{\rightleftharpoons}} X, \varnothing \underset{k_Y}{\overset{k_Y}{\rightleftharpoons}} Y$. The steady-state ensemble is unbiased, $p_X = p_Y = 1/2$, since $\Delta\tilde{G} = 0$. However, c_X and c_Y can be very different

at short times. Starting with initial conditions $c_X(0) = c_Y(0) = 0$, $\lim_{t\to 0} p_X(t) = \frac{k_X}{k_X+k_Y}$, which is only bounded by 0 and 1. A catalyst that prefers X can temporarily achieve arbitrary specificity in the product ensemble even though $\Delta\tilde{G} = 0$, as demonstrated experimentally in Cabello-Garcia et al.[35]

DISCUSSION

We have derived thermodynamic bounds on the accuracy of arbitrarily complex catalytic molecular templating networks and shown that systems at these bounds operate in pseudo-equilibrium with negligible cyclic flux. The fundamental $\Delta\tilde{G}$-dependent bounds on accuracy provide a basis on which to build our understanding of molecular information transmission, even if they are hard to achieve in practice. Indeed, Equation 5 shows how our fundamental bounds directly influence the accuracy of templating systems operating far from pseudo-equilibrium, as in biology.

Why, then, do cells not operate in pseudo-equilibrium if it is an efficient and accurate limit? First, we have derived bounds, not constructed realistic systems that approach them. In Note S5 and Figures S1 and S2, we give an explicit kinetic model of a templating network that saturates the bounds as rate constants are tuned. However, the network is contrived, and it is hard to imagine a system in which, for example, production and degradation by an RNA polymerase are balanced. Moreover, not all networks can reach the bounding accuracy, even if the rate constants in a network can be tuned arbitrarily. We provide an example in Note S6 and Figures S3–S7.

When considering the plausibility of pseudo-equilibria, it is worth noting the generality of the principle. The role of pseudo-equilibria in other contexts, such as the responsive systems of Arunachalam and Lin[22] and Floyd et al.,[23] is currently underexplored. As we illustrate in Note S7, similar results apply to networks in which substrates are activated and deactivated by catalysts, like kinase push-pull networks.[36] In these settings, balancing activation and deactivation by a single (much less complicated) pathway is more plausible. Nonetheless, there are also disadvantages to operating in the pseudo-equilibrium limit, which may explain why natural kinase cascades do not operate in this way. First, outputs are binary: the steady-state concentration does not depend on the precise template concentration. Biological systems likely benefit from analog control. Second, switching between high and low pseudo-equilibria likely requires the removal of one catalyst and the introduction of another rather than just changing the levels of a single catalyst. Synthetic information-processing systems, however, may have different requirements from nature: a binary output is often preferable, products may be relatively simple, and fast switching may not be important. Synthetic pseudo-equilibrium networks may, therefore, hold promise; we illustrate a plausible nanotechnological system that could operate in pseudo-equilibrium, based on 4-way DNA strand exchange,[37,38] in Figure S8.

We have noted that the behavior of catalytic molecular templating systems is qualitatively distinct from an equilibrium competition between a disordered, high-entropy state and a low-energy state. We now also compare our findings with previous results on discrimination during non-equilibrium polymerization. Aside from Genthon et al.,[21] which we have discussed in detail, prior work has focused on the chemical work done and/or the entropy production when creating a single polymer; these models describe a single branched pathway within our framework. For example, Sartori and Pigolotti[15] showed that an excess chemical work per monomer is required to continuously grow an infinite-length product with a non-equilibrium distribution of single-monomer copy errors. As we have shown elsewhere,[25] this apparent minimal work instead contributes to a kinetic barrier when the separation of products from templates is explicitly considered, with the minimal work then determined by the properties of the monomer and product ensembles. In either case, these lower bounds are the minimal (reversible) work to change the state of the system, and they do not imply a need for separate pathways with distinct free-energy changes. By contrast, the constraints on $\Delta\tilde{G}$ that we have derived are requirements on the diversity of assembly pathways in a reaction network to maintain a steady-state ensemble with certain properties—a steady state that cannot even be described in a formalism that focuses on a single growth event.

We also consider how our results fit within the thermodynamic and kinetic discrimination paradigms in previous work.[39] In a catalytic system, all discrimination is kinetic, even in the stationary state, since catalysts do not shift equilibria. Our claim that catalytic specificity is not limited by thermodynamics is simply that differences in barrier heights (δ in Sartori and Pigolotti[39]) can be arbitrarily large in principle. When δ is large, high discrimination is possible with low-entropy production.

Although we consider a broad class of networks, we have made a number of assumptions in our analysis. Firstly, we have assumed a symmetric model in which all products are equivalent. In a more general setting, reaction pathways, $\delta\tilde{G}_U^{Z_i}$ and $\delta\tilde{G}_L^{Z_i}$ would be product specific, and it would be easier to create distributions that favor certain stable products and harder in other cases (although given that cells need functional, rather than stable, products, it is unclear whether this asymmetry would be beneficial). Nonetheless, differences in $\delta\tilde{G}$ would still bound accuracy, and the limiting accuracy would still be obtained when products are in pseudo-equilibrium at either their maximum ($e^{-\delta\tilde{G}_U^{Z_i}}$) or minimum ($e^{-\delta\tilde{G}_L^{Z_i}}$) concentrations. This reasoning applies to ensembles with products of different lengths or folding free energies, non-product-symmetric network topologies, and even ensembles with prematurely released products.

We have also assumed that the networks are linear. The most likely violation of linearity is that sequestration of templates and catalysts influences their concentration rather than those concentrations being held constant by chemostatting. This non-linearity would make solving for steady-state concentrations of products much harder, but it has a surprisingly small effect on our conclusions.[40] Firstly, the free energy of a formation pathway S remains well defined and is unaffected by sequestration of catalysts by intermediates. Secondly, were we to construct a model with fixed total concentration of each catalyst j, d_j^T, we would find a steady state with a reduced concentration of free catalysts $d_j^* \leq d_j^T$. However, from the structure of CRNs, that solution would necessarily be identical to one in which all catalysts

were chemostatted at their respective free concentrations, d_j^*. As a consequence, any pathway free-energy-dependent constraint on product abundances that holds in all chemostatted systems also applies for fixed total catalyst concentrations. For catalytic templating networks, $\Delta\tilde{G}$ sets the same bounds on accuracy, and these bounds are reached in pseudo-equilibrium.

METHODS

Details regarding the methods are included in the results section, and further details of derivations can be found in the supplemental information.

RESOURCE AVAILABILITY

Lead contact

Requests for further information and resources should be directed to and will be fulfilled by the lead contact, Thomas E. Ouldridge (t.ouldridge@imperial.ac.uk).

Materials availability

This study did not generate new materials.

Data and code availability

Code supporting the findings of this study is openly available at https://zenodo.org/records/15554491.

ACKNOWLEDGMENTS

We thank Pieter Rein ten Wolde for his comments on the manuscript. This work is part of a project that has received funding from the European Research Council (ERC) under the European Union's Horizon 2020 research and innovation program (grant agreement no. 851910). T.E.O. was supported by a The Royal Society is the funder Research Fellowship (grant no. UF150067) and Fellowship Renewal (grant no. URF\R\211020).

AUTHOR CONTRIBUTIONS

Conceptualization, B.Q., J.M.P., and T.E.O.; methodology, B.Q. and T.E.O.; investigation, B.Q.; writing – original draft, B.Q.; writing – review & editing, J.M.P. and T.E.O.; funding acquisition, T.E.O.; supervision, J.M.P. and T.E.O.

DECLARATION OF INTERESTS

The authors declare no competing interests.

SUPPLEMENTAL INFORMATION

Supplemental information can be found online at https://doi.org/10.1016/j.newton.2025.100302.

Received: June 11, 2025
Revised: August 20, 2025
Accepted: October 23, 2025
Published: November 14, 2025

REFERENCES

1. Crick, F. (1970). Central dogma of molecular biology. Nature *227*, 561–563.
2. Ouldridge, T.E., and Rein Ten Wolde, P. (2017). Fundamental costs in the production and destruction of persistent polymer copies. Phys. Rev. Lett. *118*, 158103.
3. Watson, J.D., Gann, A., Baker, T.A., Levine, M., Bell, S.P., Losick, R., and Harrison, S.C. (2013). Molecular Biology of the Gene (Cold Spring Harbour Laboratory Press).
4. Hopfield, J.J. (1974). Kinetic proofreading: a new mechanism for reducing errors in biosynthetic processes requiring high specificity. Proc. Natl. Acad. Sci. USA *71*, 4135–4139.
5. Ninio, J. (1975). Kinetic amplification of enzyme discrimination. Biochimie *57*, 587–595.
6. Sahoo, M., Arsha, N., Baral, P.R., and Klumpp, S. (2021). Accuracy and speed of elongation in a minimal model of DNA replication. Phys. Rev. E *104*, 034417.
7. Song, Y., and Hyeon, C. (2020). Thermodynamic cost, speed, fluctuations, and error reduction of biological copy machines. J. Phys. Chem. Lett. *11*, 3136–3143.
8. Li, Q.-S., Zheng, P.-D., Shu, Y.-G., Ou-Yang, Z.-C., and Li, M. (2019). Template-specific fidelity of dna replication with high-order neighbor effects: A first-passage approach. Phys. Rev. E *100*, 012131.
9. Wong, F., Amir, A., and Gunawardena, J. (2018). Energy-speed-accuracy relation in complex networks for biological discrimination. Phys. Rev. E *98*, 012420.
10. Bennett, C.H. (1979). Dissipation-error tradeoff in proofreading. Biosystems *11*, 85–91.
11. Banerjee, K., Kolomeisky, A.B., and Igoshin, O.A. (2017). Elucidating interplay of speed and accuracy in biological error correction. Proc. Natl. Acad. Sci. USA *114*, 5183–5188.
12. Chiuchiù, D., Tu, Y., and Pigolotti, S. (2019). Error-speed correlations in biopolymer synthesis. Phys. Rev. Lett. *123*, 038101.
13. Gaspard, P., and Andrieux, D. (2014). Kinetics and thermodynamics of first-order markov chain copolymerization. J. Chem. Phys. *141*, 044908.
14. Poulton, J.M., Ten Wolde, P.R., and Ouldridge, T.E. (2019). Nonequilibrium correlations in minimal dynamical models of polymer copying. Proc. Natl. Acad. Sci. USA *116*, 1946–1951.
15. Sartori, P., and Pigolotti, S. (2015). Thermodynamics of error correction. Phys. Rev. X *5*, 041039.
16. Qureshi, B., Juritz, J., Poulton, J.M., Beersing-Vasquez, A., and Ouldridge, T.E. (2023). A universal method for analyzing copolymer growth. J. Chem. Phys. *158*, 104906.
17. Gaspard, P. (2020). Template-directed growth of copolymers. Chaos *30*, 043114.
18. Juritz, J., Poulton, J.M., and Ouldridge, T.E. (2022). Minimal mechanism for cyclic templating of length-controlled copolymers under isothermal conditions. J. Chem. Phys. *156*, 074103.
19. Guntoro, J.E.B., Qureshi, B.J., and Ouldridge, T.E. (2025). The interplay of heterogeneity and product detachment in templated polymer copying. J. Chem. Phys. *162*, 055103.
20. Bennett, C.H. (1982). The thermodynamics of computation—a review. Int. J. Theor. Phys. *21*, 905–940.
21. Genthon, A., Modes, C.D., Jülicher, F., and Grill, S.W. (2025). Non equilibrium transitions in a polymer replication ensemble. Phys. Rev. Lett. *134*, 068402.
22. Arunachalam, E., and Lin, M.M. (2025). Information Gain Limit of Biomolecular Computation. Phys. Rev. Lett. *134*, 148401.
23. Floyd, C., Dinner, A.R., Murugan, A., and Vaikuntanathan, S. (2025). Limits on the computational expressivity of non-equilibrium biophysical processes. Nat. Commun. *16*, 7184.
24. Seifert, U. (2012). Stochastic thermodynamics, fluctuation theorems and molecular machines. Rep. Prog. Phys. *75*, 126001.
25. Poulton, J.M., and Ouldridge, T.E. (2021). Edge-effects dominate copying thermodynamics for finite-length molecular oligomers. New J. Phys. *23*, 063061.

CellPress
OPEN ACCESS

26. Ouldridge, T.E. (2018). The importance of thermodynamics for molecular systems, and the importance of molecular systems for thermodynamics. Nat. Comput. *17*, 3–29.
27. Polettini, M., and Esposito, M. (2014). Irreversible thermodynamics of open chemical networks. i. emergent cycles and broken conservation laws. J. Chem. Phys. *141*, 024117.
28. Rao, R., and Esposito, M. (2016). Nonequilibrium thermodynamics of chemical reaction networks: Wisdom from stochastic thermodynamics. Phys. Rev. X *6*, 041064.
29. Anderson, D.F., Craciun, G., and Kurtz, T.G. (2010). Product-form stationary distributions for deficiency zero chemical reaction networks. Bull. Math. Biol. *72*, 1947–1970.
30. Maes, C., and Netočnỳ, K. (2013). Heat bounds and the blowtorch theorem. Ann. Henri Poincaré *14*, 1193–1202.
31. Sáez, M., Feliu, E., and Wiuf, C. (2019). Linear elimination in chemical reaction networks. In Recent Advances in Differential Equations and Applications, J.L. García Guirao, J.A. Murillo Hernández, and F. Periago Esparza, eds. (Springer International Publishing), pp. 177–193.
32. Nam, K.-M., Martinez-Corral, R., and Gunawardena, J. (2022). The linear framework: using graph theory to reveal the algebra and thermodynamics of biomolecular systems. Interface Focus *12*, 20220013.
33. Çetiner, U., and Gunawardena, J. (2022). Reformulating nonequilibrium steady states and generalized hopfield discrimination. Phys. Rev. E *106*, 064128.
34. Shannon, C.E. (1948). A mathematical theory of communication. Bell Syst. Tech. J. *27*, 379–423.
35. Cabello-Garcia, J., Mukherjee, R., Bae, W., Stan, G.-B.V., and Ouldridge, T.E. (2025). Information propagation through enzyme-free catalytic templating of DNA dimerization with weak product inhibition. Nat. Chem. *17*, 1179–1187.
36. Stock, A.M., Robinson, V.L., and Goudreau, P.N. (2000). Two-component signal transduction. Annu. Rev. Biochem. *69*, 183–215.
37. Mullor Ruiz, I. (2021). Development of a Framework for Designing Nucleic Acid-Based, Out-Of-Equilibrium Catalytic Reaction Networks. PhD Thesis Imperial College London London, UK.
38. Lankinen, A., Mullor Ruiz, I., and Ouldridge, T.E. (2020). Implementing Non-Equilibrium Networks with Active Circuits of Duplex Catalysts. In 26th International Conference on DNA Computing and Molecular Programming (DNA 26), C. Geary and M.J. Patitz, eds. (Schloss Dagstuhl – Leibniz-Zentrum für Informatik), pp. 7:1–7:25.
39. Sartori, P., and Pigolotti, S. (2013). Kinetic versus energetic discrimination in biological copying. Phys. Rev. Lett. *110*, 188101.
40. Liang, S., De Los Rios, P., and Busiello, D.M. (2024). Thermodynamic bounds on symmetry breaking in linear and catalytic biochemical systems. Phys. Rev. Lett. *132*, 228402.

NEWTON, Volume *2*

Supplemental information

Thermodynamic limits in far-from-equilibrium molecular templating networks

Benjamin Qureshi, Jenny M. Poulton, and Thomas E. Ouldridge

Supplemental Figures

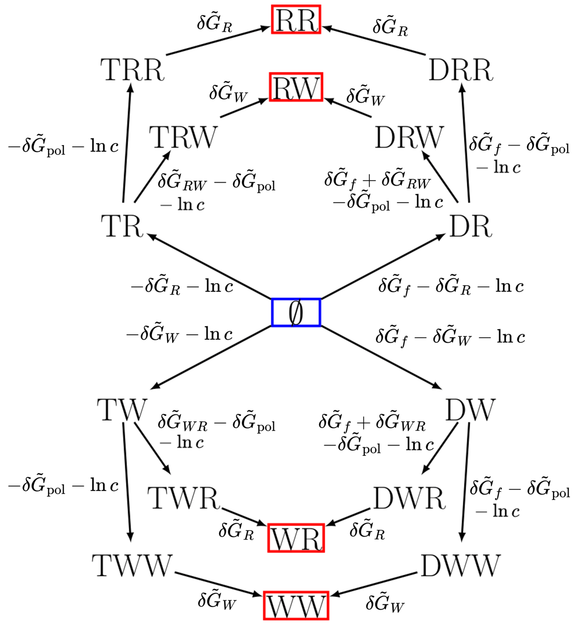

Figure S1: **Linearised CRN for a system in which dimers are grown/destroyed via a template "T" and by a destructive template "D".** The system starts in the null state (blue) and a right or wrong ("R" and "W") monomer attach to either the template "T" or destructive enzyme "D". A second monomer can attach and polymerize with the first, yielding to a dimer that then detaches, giving the four red output states. Each arrow represents a reversible reaction, with the free-energy change in direction of the arrow indicated. For brevity, $\delta\tilde{G}_{RW} = -\delta\tilde{G}_{WR} = \delta\tilde{G}_R - \delta\tilde{G}_W$. To reach each red product node, there are four self-avoiding walks from the blue null state.

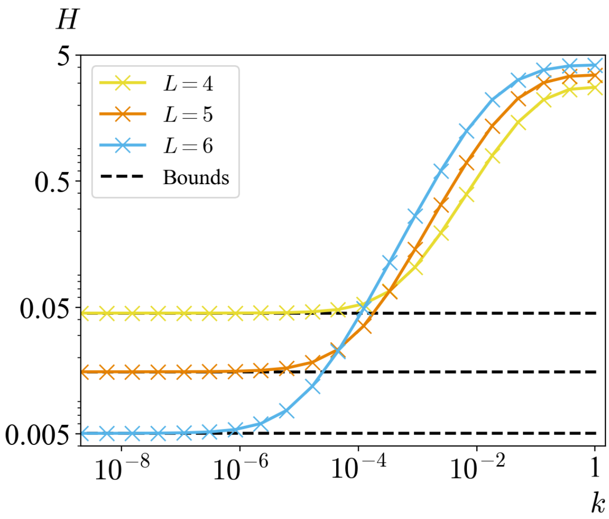

Figure S2: **The entropy bound may be saturated by model systems in the limit that some reaction rates are much smaller than others.** We plot the entropy, H, of the product distribution as a function of the slow reaction rates, k, for different template lengths, L, for the simple production and destruction model introduced in Note S5. The data is obtained for a fixed $\delta\tilde{G}_f = 2$, $\delta\tilde{G}_{\mathrm{pol}} = 0$, $\delta\tilde{G}_R = 2$, $\delta\tilde{G}_W = -2$ and $c = 1$. Note that longer templates reach a lower entropy bound for a fixed fuel turnover per unit length, $\delta\tilde{G}_f$.

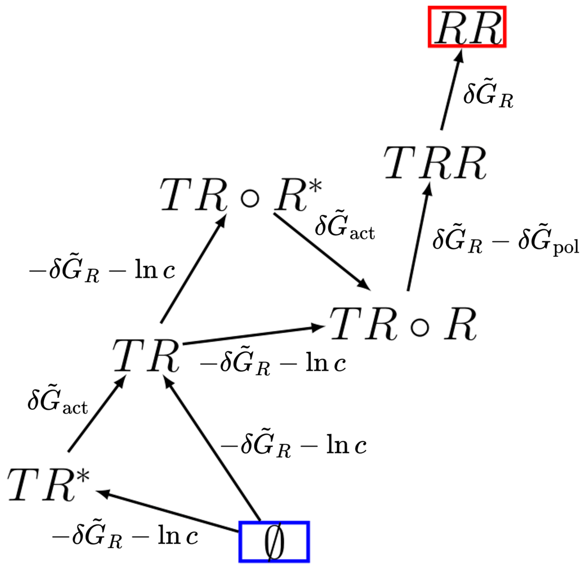

Figure S3: **A templating model incorporating kinetic proofreading.** We modify the template-based reactions of the model in Note S5. We show here the template-based reactions leading directly to the dimer RR, which should replace the equivalent template-based reactions in S1. As before, arrows represent reversible reactions with free-energy change in the direction of the arrow indicated. On the template, from state TR, either a non-activated (R^*) or activated (R) monomer may bind to the template. If that monomer has bound, but has not yet been polymerised into the growing polymer, it is represented by $TR \circ R^*$ or $TR \circ R$. When bound to the template, non-activated monomers may be activated, as shown by the transitions in which R^* is converted to R. When there is an activated monomer at the end of the growing polymer ($TR \circ R$), that monomer may be polymerised into the growing polymer to reach a polymerised state (TRR). After a full length polymer has grown on the template, it may detach to a product (RR for a dimer template).

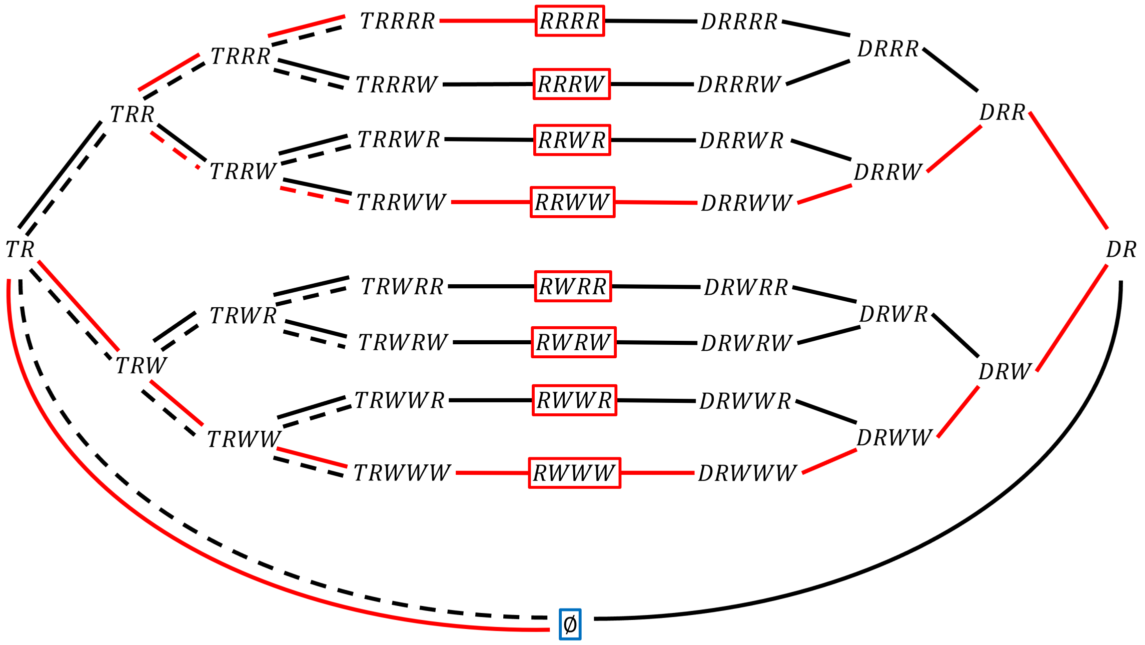

(a)

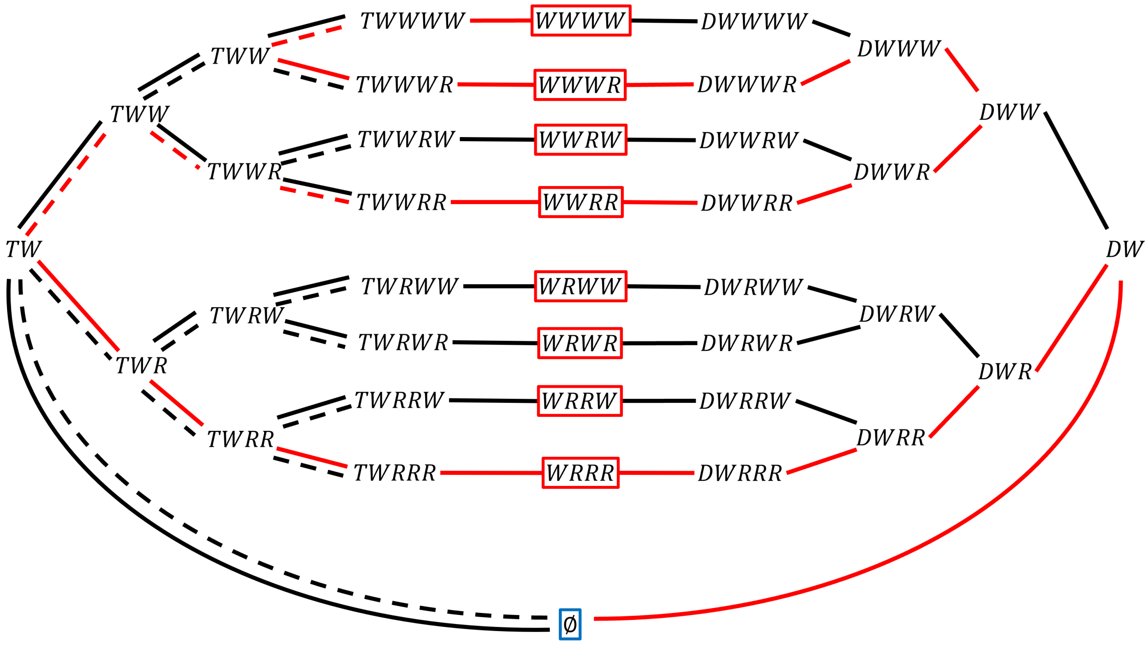

(b)

Figure S4: **Pathways with most positive and most negative free energy changes that we have identified in a model with kinetic proofreading.** (a) The pathway to $RRRR$ with free energy change $\delta\tilde{G}^*_U$ given by eq. S23 is shown in red. (b) The pathway to $WWWW$ with free energy change $\delta\tilde{G}^*_L$ given by eq. S24 is shown in red. For each of these diagrams, only the relevant half the reaction network is shown for simplicity. In these networks, there are two pathways between different template-bound states such as TRR and TRRR; one proceeding via R^* and one bia directly binding to R (figure S3). We represent these pathways via the solid and dashed lines, respectively.

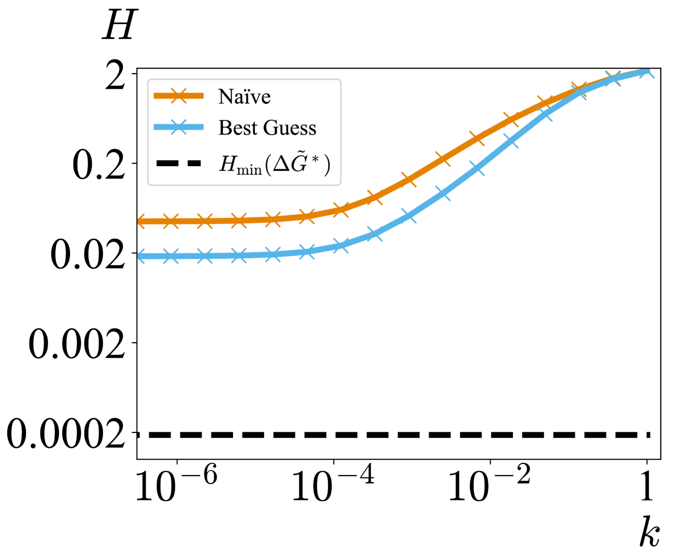

Figure S5: **Minimising product ensemble entropy in a model with kinetic proofreading.** Entropy H for two attempts to optimise the entropy, plotted alongside the lower bound $H_{\min}(\Delta\tilde{G}^*)$ for a system with $\Delta\tilde{G} = \Delta\tilde{G}^*$ implied by eqs. S23 and S24, as a function of the parameter k that sets the overall scale of slow reactions relative to fast ones. In the "naïve" approach, rates favour pathways to the correct product on the template and the incorrect ones on the destroyer. The "best guess" favours the snaking pathways described in text. The data is obtained for $\delta\tilde{G}_f = 1$, $\delta\tilde{G}_{\rm act} = 1$, $\delta\tilde{G}_{\rm pol} = 0$, $\delta\tilde{G}_R = 2$, $\delta\tilde{G}_W = -2$ and $c = 1$.

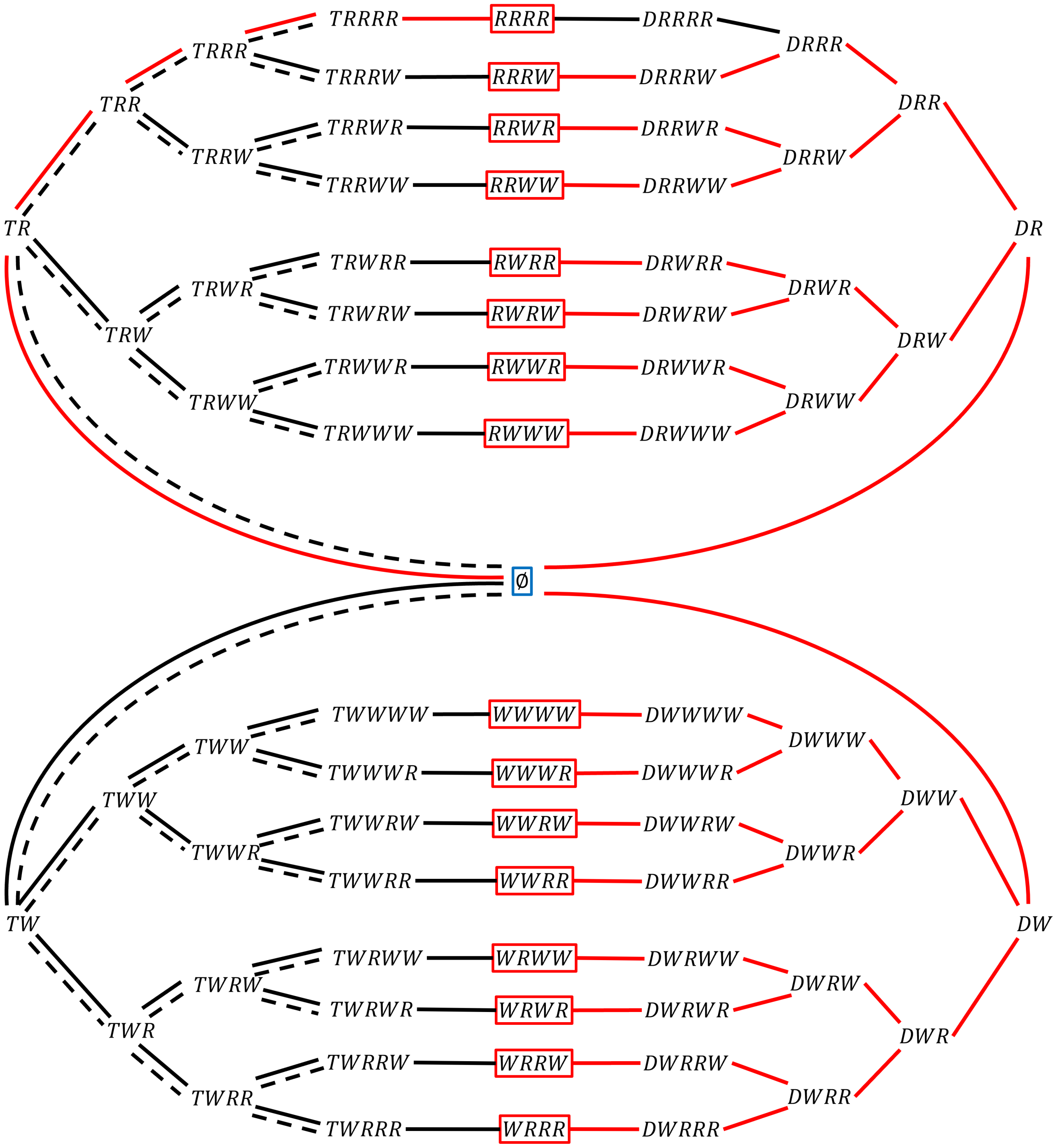

Figure S6: **Fast pathways in a naïve attempt to minimize the entropy of the product distribution for a model with kinetic proofreading.** Fast reactions with rate ~ 1 are shown in red. In these networks, there are two pathways between different template-bound states such as TRR and TRRR; one proceeding via R^* and one bia directly binding to R (figure S3). We represent these pathways via the solid and dashed lines, respectively.

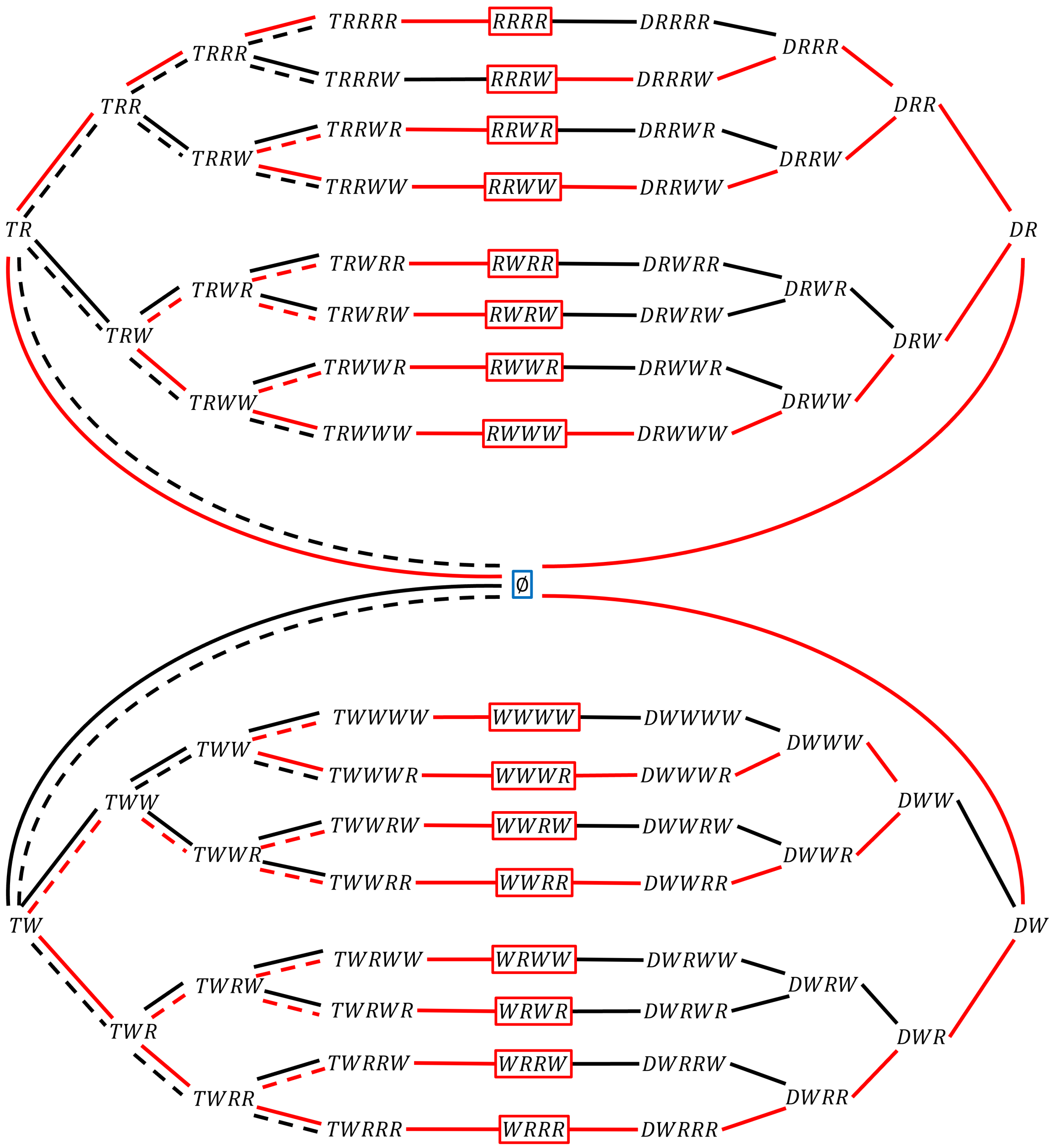

Figure S7: **Fast pathways in our best guess attempt to minimize the entropy of the product ensemble for a model with kinetic proofreading.** Fast reactions with rate ~ 1 are shown in red. In these networks, there are two pathways between different template-bound states such as TRR and TRRR; one proceeding via R^* and one bia directly binding to R (figure S3). We represent these pathways via the solid and dashed lines, respectively.

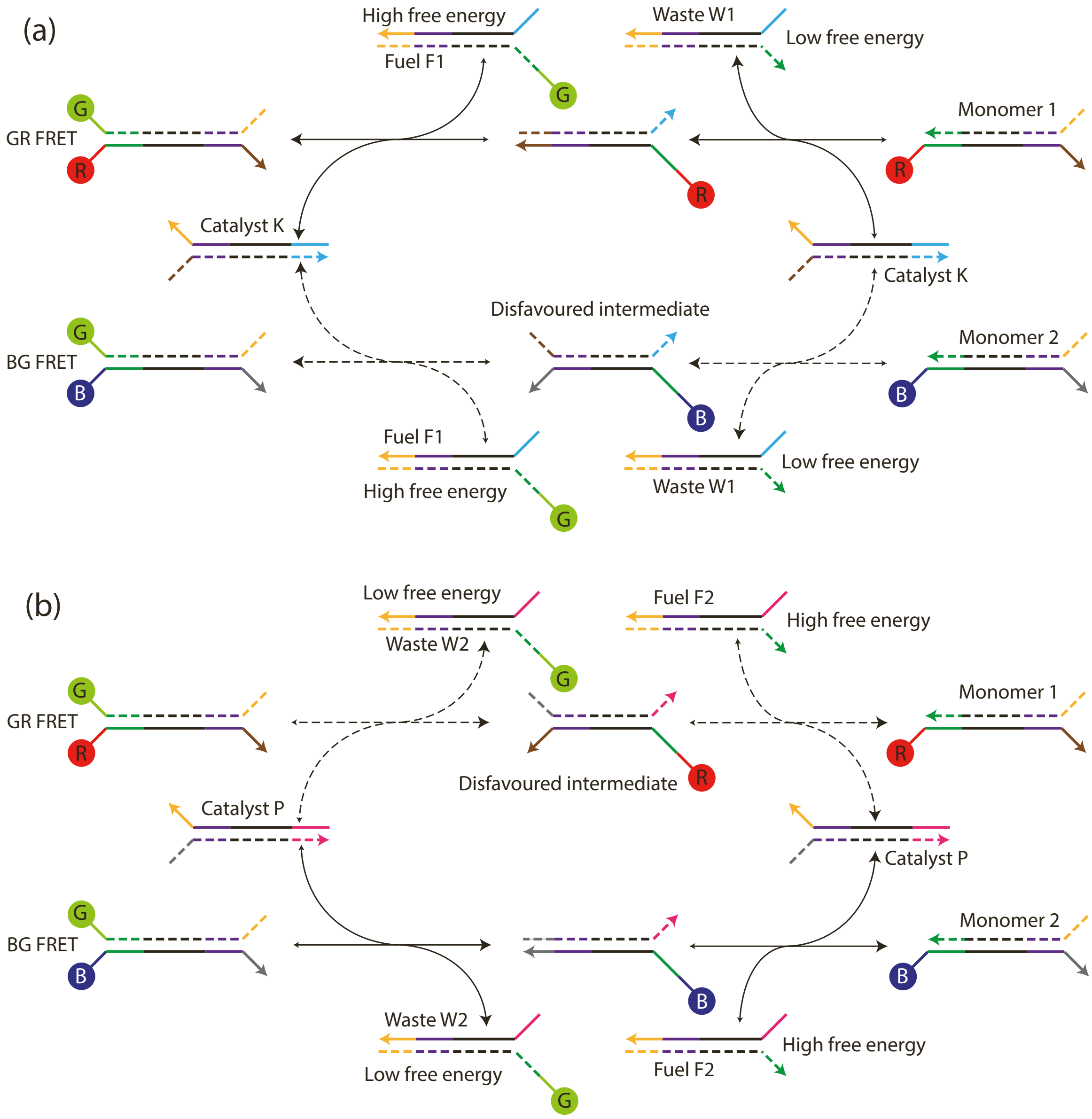

Figure S8: **A DNA strand displacement network, based on the ACDC design of Ref.[S1,S2], that is capable of demonstrating high specificity in pseudo-equilibrium.** Two monomer species (Monomer 1 and Monomer 2) can be activated and deactivated by fuel-coupled, catalytically controlled pathways. Activated species have colocalised fluorophores that can be identified by FRET signals. Lines represents DNA strands, and single-coloured sections of strands are domains of bases that act as a coherent unit, with solid sections of one colour being complementary to dashed sections of the same colour. Fluorophores are additionally labelled with R/B/G for clarity. Arrowheads on the DNA strands indicate $5' \to 3'$ directionality. Each reaction occurs via 4-way strand exchange[S1,S2] initiated by the binding of available and complementary toeholds (the shorter domains, of length ~ 5 bases). The size of the black reaction arrows indicate whether a reaction is driven forwards or backwards by the free energy difference between the fuel and waste. (a) Activation of Monomer 1 and Monomer 2 by a DNA duplex catalyst K coupled to the consumption of fuel F_1 and the production of waste W_1. The reaction is driven to the left (activation is thermodynamically favoured) by an excess of F_1 over W_1 and/or mismatches in the initial F_1 duplex that are eliminated in W_1[S1,S2]. Activation of Monomer 2 along this pathway has the same free-energy change as Monomer 1, but is kinetically very slow because the intermediate for Monomer 2 has a mismatched toehold domain. (b) A second pathway by which monomers can be interconverted between activated states, coupled to the catalyst P fuel F_2 and waste W_2. Unlike the reaction pathway in (a), the pathway in (b) is driven to the right and the catalyst P preferentially couples to Monomer 2 due to its toehold sequence.

Supplemental Notes

Note S1 Definition of topological equivalence

Let $\mathcal{G}$ be the vertex-labelled graph describing the linear CRN containing a vertex corresponding to the null complex $\emptyset$ and a set of vertices corresponding to products, ignoring the edge-weights. Then for any pair of products, X and Y, consider a relabelling of the nodes such that the nodes for X and Y swap labels, $\emptyset$'s label is not changed, but other node labels may be permuted. Suppose there exists such a relabelling, transforming the graph to a new vertex-labelled graph $\mathcal{G}'$ such that $\mathcal{G}'$ is isomorphic as a labelled graph to $\mathcal{G}$. Then the set of SAWs from $\emptyset$ to X will be topologically equivalent to those SAWs from $\emptyset$ to Y. Further, a given SAW from $\emptyset$ to X will be topologically equivalent to a specific SAW from $\emptyset$ to Y. If these equivalent SAWs also incur the same free-energy change, then the model falls into the framework of models we discuss in the paper.

Note S2 Proof of boundedness of steady-state concentrations

Note S2.A The steady state concentration written as sum over spanning trees

We first give a useful result for the concentration of a species in terms of the graph of the linear CRN. We note that a similar result can be seen in *e.g.*[S3]. However, we prove this result here with particular reference to the effect of a null species.

Consider a linear connected CRN under mass action kinetics. Let X_i be the chemical species with concentration c_i. Assume the CRN contains some reactions of the form $\emptyset \rightleftharpoons X$. Without loss of generality, we assume that there only exists at most one reaction of the form $X_i \to X_j$ (for the cases where there are multiple such reactions, replace their reaction rate with the sum over all reaction rates of reactions of that form). We may cast the steady state equation for the vector of steady state concentrations of the chemical species, $\mathbf{c}$, as the linear equation:

$$A\mathbf{c} + \mathbf{b} = \mathbf{0}, \tag{S1}$$

where $\mathbf{b}$ is a vector such that entry b_i is the rate constant of the reaction $\emptyset \to X_i$, and A is a matrix with off diagonal entries A_{ij} equal to the rate constant of the reaction $X_j \to X_i$ and diagonal elements A_{ii} equal to minus the sum of reaction rates of reactions $X_i \to X_j$ over all chemical species X_j $j \neq i$ as well as $X_i \to \emptyset$. Note that the sum of column i of matrix A is equal to minus the rate constant of reaction $X_i \to \emptyset$. Thus, we may create the new matrix:

$$K = \left(\begin{array}{c|c} A & \mathbf{b} \\ \hline \mathbf{d}^T & -\sum\limits_i b_i \end{array}\right), \tag{S2}$$

where $\mathbf{d}$ is a vector such that entry d_i is equal to the rate constant of reaction $X_i \to \emptyset$. The columns of the matrix, K, now sum to zero and can be recognised as the Laplacian matrix of a certain graph. Represent K as a graph with nodes corresponding to chemical species X_i and an additional node corresponding to $\emptyset$, and edges e corresponding to reactions between species with weights equal to their rate constant $k(e)$. Then, by the matrix tree theorem[?] , the determinant (up to a sign) of the sub matrix formed by deleting row and column i from matrix K is given by the sum over the set of spanning trees rooted at node i, $\mathcal{T}(X_i)$,

$$\det(K/i) = \sum_{T \in \mathcal{T}(X_i)} \prod_{e \in T} k(e), \tag{S3}$$

where (K/i) represents matrix K with row and column i deleted. In particular,

$$\det(A) = \sum_{T \in \mathcal{T}(\emptyset)} \prod_{e \in T} k(e). \tag{S4}$$

Thus, by Cramers rule[S4], and the sign of the determinant under swapping of rows,

$$c_X = \frac{\sum\limits_{T \in \mathcal{T}(X)} \prod\limits_{e \in T} k(e)}{\sum\limits_{T \in \mathcal{T}(\emptyset)} \prod\limits_{e \in T} k(e)}, \tag{S5}$$

gives the solution to eq. S1.

Note S2.B Bounding steady-state concentrations

We note that similar proofs exist in the literature[S3,S5–S8], but we have included the proof here for completeness and to match our specific conventions. Further, in the cases considered in this paper, the fact that all products are connected to the null state means that our result bounds the absolute, rather than a relative, concentrations.

Consider a linear connected CRN with chemical species X_i and some reactions of the form $\emptyset \rightleftharpoons X_i$. The concentration of species X_i may be written as in eq S5. The numerator and denominator in this fraction are sums over spanning trees rooted at a given node. A sum over spanning trees rooted at node X may be factored into a sum over self avoiding walks (SAWs) from some arbitrary other node to node X. Concretely, letting Y be the other, arbitrary node, and $S(Y \to X)$ be the set of SAWs from Y to X,

$$\sum_{T \in \mathcal{T}(X)} \prod_{e \in T} k(e) = \sum_{S \in S(Y \to X)} A(S) \prod_{e \in S} k(e), \tag{S6}$$

where $A(S)$ is a factor that, crucially, is the same for the equivalent (reversed) SAW in $S(X \to Y)$, in which all edges are reversed compared to $S(Y \to X)$. That is to say, if we now wish to find the sum over spanning trees rooted at Y, we may choose X as the arbitrary other state and find:

$$\sum_{T \in \mathcal{T}(Y)} \prod_{e \in T} k(e) = \sum_{S \in S(Y \to X)} A(S) \prod_{e \in S} k(\bar{e}), \tag{S7}$$

where $\bar{e}$ is the reverse of edge e. For the linear CRNs, utilising

$$\delta\tilde{G}_S = -\ln \left(\prod_{e \in S} \frac{k(e)}{k(\bar{e})} \right), \tag{S8}$$

which follows from applying local detailed balance to each step of the SAW[S9], we may write:

$$\begin{aligned} c_X &= \frac{\sum\limits_{S \in S(\emptyset \to X)} A(S) \prod\limits_{e \in S} k(e)}{\sum\limits_{S \in S(\emptyset \to X)} A(S) \prod\limits_{e \in S} k(\bar{e})} \\ &= \frac{\sum\limits_{S \in S(\emptyset \to X)} A(S) \left[\prod\limits_{e \in S} k(\bar{e}) \right] e^{-\delta\tilde{G}_S}}{\sum\limits_{S \in S(\emptyset \to X)} A(S) \prod\limits_{e \in S} k(\bar{e})}. \end{aligned} \tag{S9}$$

Hence,

$$c_X \in \left[e^{-\max\limits_{S \in S(\emptyset \to X)} (\delta G_S)}, e^{-\min\limits_{S \in S(\emptyset \to X)} (-\delta G_S)} \right], \tag{S10}$$

as required.

Note S3 Proof of the boundedness of steady-state distribution entropy

We have a set of M concentrations $\{c_1, \dots c_M\}$. Denote the total concentration $c_T = \sum\limits_{i=1}^{M} c_i$. Suppose that each concentration is bounded by the same upper and lower bounds, $c_i \in [c_L, c_U]$. We now propose that the distribution of concentrations that minimises the Shannon entropy, $H([c_i])$, is that with $m_{\min}$ of the species at concentration c_U and $M - m_{\min}$ at concentration c_L, where $m_{\min}$ is either

$$\left\lceil \frac{\frac{c_L}{c_U}\left[-\ln\left(\frac{c_L}{c_U}\right) - \left(1 - \frac{c_L}{c_U}\right)\right]}{\left(1 - \frac{c_L}{c_U}\right)^2} M \right\rceil \text{ or}$$

$$\left\lceil \frac{\frac{c_L}{c_U}\left[-\ln\left(\frac{c_L}{c_U}\right) - \left(1 - \frac{c_L}{c_U}\right)\right]}{\left(1 - \frac{c_L}{c_U}\right)^2} M \right\rceil - 1. \tag{S11}$$

To prove this claim, let us calculate the derivative of $H = H([p_i])$ with respect to a concentration c_α, holding all other concentrations fixed and remembering that c_T is linear in c_α,

$$\frac{\partial H}{\partial c_\alpha} = \frac{1}{c_T} \left(-\ln\left(\frac{c_\alpha}{c_T}\right) - H \right). \tag{S12}$$

This H has a local maximum or minimum at $-\ln\left(\frac{c_\alpha}{c_T}\right) = H$. To proceed, we require the second derivative,

$$\frac{\partial^2 H}{\partial c_\alpha^2} = -\frac{1}{c_T^2}\left(-\ln\left(\frac{c_\alpha}{c_T}\right) - H\right) - \frac{1}{c_T}\frac{\partial H}{\partial c_\alpha} - \frac{1}{c_T}\frac{1}{c_\alpha}\left(1 - \frac{c_\alpha}{c_T}\right). \tag{S13}$$

Evaluating the second derivative at $-\ln\left(\frac{c_\alpha}{c_T}\right) = H$, the first two terms are zero and the third is necessarily negative for non-zero c_T. Thus

$$\left.\frac{\partial^2 H}{\partial c_\alpha^2}\right|_{-\ln\left(\frac{c_\alpha}{c_T}\right)=H} < 0. \tag{S14}$$

And so, for any distribution, we can decrease H by increasing the concentrations of any species i for which $-\ln\left(\frac{c_i}{c_T}\right) < H$ and decreasing the concentrations for any species whose concentration has $-\ln\left(\frac{c_i}{c_T}\right) > H$. For species for which $-\ln\left(\frac{c_i}{c_T}\right) = H$, changing the concentration in either direction will decrease H. Consequently, minimising the entropy of the distribution necessarily requires all species to be at one bound or the other: we need m species at concentration c_U and $M - m$ at concentration c_L. Hence, we have transformed the problem into a one dimensional one of minimising H as a function of m. We can write this entropy as

$$H(m) = -\frac{(M-m)\frac{c_L}{c_U}\ln\left(\frac{c_L}{c_U}\right)}{(M-m)\frac{c_L}{c_U} + m} + \ln\left((M-m)\frac{c_L}{c_U} + m\right). \tag{S15}$$

Taking the derivative with respect to m and setting it to zero tells us that

$$m_{\min} = \frac{\frac{c_L}{c_U}\left[-\ln\left(\frac{c_L}{c_U}\right) - \left(1 - \frac{c_L}{c_U}\right)\right]}{\left(1 - \frac{c_L}{c_U}\right)^2} M \tag{S16}$$

gives a turning point for $H(m)$, which is clearly a minimum since $H(0) = H(M) = \ln M$ is maximal entropy. Since $H(m)$ has only a single turning point as a function of m, the integer that minimises $H(m)$ will be either the floor or ceiling of the above expression, and $H_{\min}$ is given by eq. 4 of the main text and eq. S11 (remembering that $c_L/c_U = \exp(-\Delta\tilde{G})$).

A good approximation to the optimal value of m is given by

$$m'_{\min} = \max\left(\frac{e^{-\Delta\tilde{G}}\left[\Delta\tilde{G} - \left(1 - e^{-\Delta\tilde{G}}\right)\right]}{\left(1 - e^{-\Delta\tilde{G}}\right)^2} M, 1\right), \tag{S17}$$

since for $m_{\min} > 1$, the difference between using the integer value of $m_{\min}$ (eq. S11) and the continuous value $m'_{\min}$ (eq. S17) is small. In practice, using eq. S17 produces a very slightly looser bound on $H[p_i]$. Further, using eq. S17, for $m_{\min} > 1$, we may simplify:

$$H_{\min} = \ln M - \Delta\tilde{G}\left(1 + \frac{e^{-\Delta\tilde{G}}}{1 - e^{-\Delta\tilde{G}}}\right) + \ln\left(\frac{\Delta\tilde{G}}{1 + e^{-\Delta\tilde{G}}}\right) + 1. \tag{S18}$$

Note S4 Minimizing entropy maximizes channel capacity

We can consider the deterministic CRNs in this paper to be information channels. Let us assume the underlying chemistry, which determines the rate constants appearing in the un-linearized model, and the concentration of monomers, are fixed. The effective rates of the linearized network would then vary with the concentrations of catalysts only. As a specific (simple) example of how the entropy bound defines the channel capacity, let us assume that all variability is due to M sequence-specific template catalysts, one for each product, and that of these templates exactly one is present at any given time at a fixed concentration.

We can define the input to the information channel as the template that is present at high concentration; the output would then be a product sampled from the steady state product distribution for that input state. We can calculate the mutual information between the template input and output products. If the templates are symmetric, acting equivalently relative to their ideal sequence, then each output distribution would merely be a permutation of the set of product probabilities.

A system of this kind would define a symmetric channel. The channel capacity of such a symmetric channel[S10] is given by $C = \ln M - H([p_i])$ in our notation, where M is the number of products/sequence-specific templates, and $H([p_i])$ the Shannon entropy of the output distribution for any single template. This entropy will be the same for any input state in our symmetrized description. Hence, minimizing the entropy maximizes the channel capacity.

Note S5 Example chemical reaction network that can saturate the bounds on accuracy

Although calculations of the bounds on accuracy is often straightforward, evaluation of the actual performance of a system realisation can be more challenging. For the examples in Note S5 and Note S6, steady state distributions are found by numerical solution of the underlying ordinary differential equations (ODEs). The CRNs are linear, connected and contain only one stoichiometric compatibility class. Hence, there exists a single positive steady state to the ODEs induced by mass action kinetics[S11]. Initially, all concentrations are set to zero, and the ODEs are simulated for a large time until no change to the distribution is observed. For the results in Note S6, to speed up simulation, we make use a of a result from[S12] whereby we may coarse grain some sets of reactions without changing the steady state.

Refs[S12,S13] studied a system in which a polymer is grown on a template. Monomers of either the "right" or the "wrong" type are added one at a time to a polymer in contact with a template, while the product polymer continually unbinds from the template from behind its leading edge. We now extend the system to include final dissociation from the template for complete polymers, and also a "destructive template". This destructive template participates in identical reactions to the template, except that polymerisation is driven backwards due to consumption of fuel molecules, meaning products tend to be destroyed rather than grown.

A full CRN for this model is shown below; we illustrate the linearised network (assuming monomers and catalysts are coupled to chemostats) for the case of dimerisation in figure S1.

$$
\begin{array}{rcl}
T + X_1 & \xrightleftharpoons[k^{T}_{X_1} e^{-\delta\tilde{G}_{X_1}}]{k^{T}_{X_1}} & TX_1 \\
TX_1\cdots X_{n-1} + X_n & \xrightleftharpoons[k^{T}_{X_1\cdots X_n} e^{-\delta\tilde{G}_{X_n} - \delta\tilde{G}_{\mathrm{pol}}}]{k^{T}_{X_1\cdots X_n} e^{-\delta\tilde{G}_{X_{n-1}}}} & TX_1\cdots X_n \\
TX_1\cdots X_L & \xrightleftharpoons[k^{T,u}_{X_1\cdots X_L}]{k^{T,u}_{X_1\cdots X_L} e^{-\delta\tilde{G}_{X_L}}} & T + X_1\cdots X_L \\
D + X_1 & \xrightleftharpoons[k^{D}_{X_1} e^{\delta\tilde{G}_f - \delta\tilde{G}_{X_1}}]{k^{D}_{X_1}} & DX_1 \\
DX_1\cdots X_{n-1} + X_n & \xrightleftharpoons[k^{D}_{X_1\cdots X_n} e^{\delta\tilde{G}_f - \delta\tilde{G}_{X_n} - \delta\tilde{G}_{\mathrm{pol}}}]{k^{D}_{X_1\cdots X_n} e^{-\delta\tilde{G}_{X_{n-1}}}} & DX_1\cdots X_n \\
DX_1\cdots X_L & \xrightleftharpoons[k^{D,u}_{X_1\cdots X_L}]{k^{D,u}_{X_1\cdots X_L} e^{-\delta\tilde{G}_{X_L}}} & D + X_1\cdots X_L,
\end{array}
\tag{S19}
$$

for $n \leq L$, $X_i \in \{R, W\}$. Dynamics is assumed to follow mass action kinetics, with rate constants given above and below the harpoons. T represents the template, D the destructive catalyst and R, W the "right" and "wrong" monomers. The products are $X_1\cdots X_L$, representing the polymers of length L. Here, there are $M = 2^L$ different products. $TX_1\cdots X_n$ and $DX_1\cdots X_n$ represent partial polymers, $X_1\cdots X_n$, bound to the template or destructive catalyst. Monomer X binds to the template or destructive catalyst with standard free-energy $-\delta\tilde{G}_X$ and the standard free-energy of polymerisation is $-\delta\tilde{G}_{\mathrm{pol}}$ in the absence of fuel. The destructive catalyst has an additional free energy $\delta\tilde{G}_f$ per length driving the disassembly of polymers.

The thermodynamics of the model are characterised by the standard polymerisation free energy of the monomers ($-\delta\tilde{G}_{\mathrm{pol}}$); the standard free-energy change of binding to the template for right and wrong monomers ($-\delta\tilde{G}_R$ and $-\delta\tilde{G}_W$), and the free energy of fuel turnover $-\delta\tilde{G}_f$. We assume that both right and wrong monomers are held at concentration c.

For the model depicted in figure S1, we can calculate the free-energy change for different paths to each product state. For example, the paths from the null state to RR are:

$$
\begin{array}{llllll}
1) & \emptyset & \to TR & \to TRR & \to RR, & \\
2) & \emptyset & \to TR & \to TRW & \to RW & \to DRW \\
 & & & \to DR & \to DRR & \to RR, \\
3) & \emptyset & \to DR & \to DRW & \to RW & \to TRW \\
 & & & \to TR & \to TRR & \to RR, \\
4) & \emptyset & \to DR & \to DRR & \to RR. &
\end{array}
\tag{S20}
$$

These incur free-energy changes:

$$\begin{aligned}
1)&\quad -(\delta\tilde{G}_{\mathsf{pol}} \quad +2\ln c),\\
2)&\quad -(\delta\tilde{G}_{\mathsf{pol}} \quad +2\ln c),\\
3)&\quad -(\delta\tilde{G}_{\mathsf{pol}} \quad +2\ln c)+\delta\tilde{G}_f,\\
4)&\quad -(\delta\tilde{G}_{\mathsf{pol}} \quad +2\ln c)+2\delta\tilde{G}_f.
\end{aligned} \tag{S21}$$

Each pathway contains the terms $-(\delta\tilde{G}_{\mathsf{pol}}+2\ln c)$, corresponding to the standard free-energy change of product formation without any fuel turnover. Since our bounds only rely on the differences in free energies between pathways, we may drop these contributions, and the fuel free energy $\delta\tilde{G}_f$ alone determines the bounds. For polymers of length L, the equivalent standard free energy of product formation is $(L-1)\delta\tilde{G}_{\mathsf{pol}}+L\ln c$, and it too may be dropped for consideration of the bounds.

Our bounds are achieved when rates of the path with the most negative free-energy change are maximised for the desired product(s), and rates of the path with the most positive free-energy change are maximised for all other products. For the $L=2$ case, for example, we could therefore maximise the rate of path 1 for RR, and the equivalents of path 4 for other products.

In fact, for this system, the entropy bound is formally achievable for arbitrary L. We can split the edges of the graph into two sets; one in which the rates are ~ 1, and one in which the rates are $\sim k$. In figure S2, we show $H[p_i]$ as $k\to 0$ for a particular choice of these sets, in which all the reactions leading directly to the fully correct sequence being created/destroyed on the template are set to be fast (not proportional to k), as are all the reactions leading directly to the creation/destruction of other sequences on the destroyer. Explicitly, set the rates of $\emptyset\to TR$, $TR\to TRR$, ..., $TR^{L-1}\to TR^L$, $TR^L\to R^L$ equal to 1 (here, R^L corresponds to L copies of R). We also set all the rates of $\emptyset\to DX_1$, $DX_1\to DX_1X_2$, ..., $DX_1...X_{L-1}\to DX1...X_L$, $DX_1...X_L\to X_1..X_L$, where $X_i=R$ or W but excluding $X_1\cdots X_L$ all being R, equal to 1. Conversely, we set the rates of $\emptyset\to DR$, $DR\to DRR$, ..., $DR^{L-1}\to DR^L$, $DR^L\to R^L$ and $\emptyset\to TX_1$, $TX_1\to TX_1X_2$, ..., $TX_1...X_{L-1}\to TX_1...X_L$, $TX_1...X_L\to X_1..X_L$, where $X_i=R$ or W but excluding $X_1\cdots X_L$ all being R, equal to $k_I=k$. The reverse reactions of those listed above have a rate determined by the free-energy change of reaction. For $k\to 0$, this set of reaction rates saturates the bound. The system saturates the entropy bound $H_{\min}$ as $k\to 0$. Note that for the value of $\delta\tilde{G}_f$ used, the minimal entropy and maximal specificity distributions are the same.

There are many possible ways to choose sets of edges that can saturate the bound in the limit $k\to 0$. Here, we have chosen a set of rates that specifically highlights a full pathway to each product for illustrative purposes. One might also wish to choose a minimum set of reactions to have rate constant k while still saturating the bound in the limit $k\to 0$. For example, letting the slow reactions be $DR^L\to R^L$, where R^L means L copies of R, and $TX_1...X_L\to X_1..X_L$, excluding $X_1\cdots X_L$ all being R, will still saturate the bound in the limit $k\to 0$. Further, we note that it is possible to saturate the bound with a non-specific destructive catalyst, where the reaction rates are independent of the polymer sequence. In the examples we have identified, such a network requires at least three rate scales $\sim 1,k,k^2$.

We stress that although the bound is formally attainable in this system, doing so relies on the ability to manipulate rate constants arbitrarily, subject to thermodynamic constraints. In a more realistic model of a templating system, constraints on relative rates may also be relevant; these constraints may stop the system reaching the bounds on accuracy or product entropy.

Note S6 Example chemical reaction network with kinetic proofreading that cannot saturate the bounds on accuracy

To illustrate the application of the bound to a more complex network, and to demonstrate the possibility of non-trivial pathways defining the bound, we consider an extension to the previous model, wherein the template also performs kinetic proofreading. First suggested by Hopfield[S14] and Ninio[S15] and widely studied[S16,S17], kinetic proofreading is a mechanism by which a system can increase the specificity of a process by expending extra free energy through fuel consuming cycles. These cycles give an extra opportunity to reject the “wrong” monomers due to their shorter binding lifetime.

The full chemical reaction network for a proofreading template of arbitrary length L is:

$$
\begin{array}{rcl}
T + X_1^* & \underset{k^{T,I}_{X_1} e^{-\delta\tilde{G}_{X_1}}}{\overset{k^{T,I}_{X_1}}{\rightleftharpoons}} & TX_1^* \\
TX_1^* & \underset{k^{T,\text{act}}_{X_1}}{\overset{k^{T,\text{act}}_{X_1} e^{\delta\tilde{G}_{\text{act}}}}{\rightleftharpoons}} & TX_1 \\
T + X_1 & \underset{k^{T}_{X_1} e^{-\delta\tilde{G}_{X_1}}}{\overset{k^{T}_{X_1}}{\rightleftharpoons}} & TX_1 \\
TX_1 \cdots X_{n-1} + X_n^* & \underset{k^{T,I}_{X_1 \cdots X_n} e^{-\delta\tilde{G}_{X_n}}}{\overset{k^{T,I}_{X_1 \cdots X_n}}{\rightleftharpoons}} & TX_1 \cdots X_{n-1} \circ X_n^* \\
TX_1 \cdots X_{n-1} \circ X_n^* & \underset{k^{T,\text{act}}_{X_1 \cdots X_n}}{\overset{k^{T,\text{act}}_{X_1 \cdots X_n} e^{\delta\tilde{G}_{\text{act}}}}{\rightleftharpoons}} & TX_1 \cdots X_{n-1} \circ X_n \\
TX_1 \cdots X_{n-1} + X_n & \underset{k^{T}_{X_1 \cdots X_n} e^{-\delta\tilde{G}_{X_n}}}{\overset{k^{T}_{X_1 \cdots X_n}}{\rightleftharpoons}} & TX_1 \cdots X_{n-1} \circ X_n \\
TX_1 \cdots X_{n-1} \circ X_n & \underset{k^{T,\text{pol}}_{X_1 \cdots X_n} e^{-\delta\tilde{G}_{\text{pol}}}}{\overset{k^{T,\text{pol}}_{X_1 \cdots X_n} e^{-\delta\tilde{G}_{X_{n-1}}}}{\rightleftharpoons}} & TX_1 \cdots X_n \\
TX_1 \cdots X_L & \underset{k^{T,u}_{X_1 \cdots X_L}}{\overset{k^{T,u}_{X_1 \cdots X_L} e^{-\delta\tilde{G}_{X_L}}}{\rightleftharpoons}} & T + X_1 \cdots X_L \\
D + X_1 & \underset{k^{D}_{X_1} e^{\delta\tilde{G}_f - \delta\tilde{G}_{X_1}}}{\overset{k^{D}_{X_1}}{\rightleftharpoons}} & DX_1 \\
DX_1 \cdots X_{n-1} + X_n & \underset{k^{D}_{X_1 \cdots X_n} e^{\delta\tilde{G}_f - \delta\tilde{G}_{X_n} - \delta\tilde{G}_{\text{pol}}}}{\overset{k^{D}_{X_1 \cdots X_n} e^{-\delta\tilde{G}_{X_{n-1}}}}{\rightleftharpoons}} & DX_1 \cdots X_n \\
DX_1 \cdots X_L & \underset{k^{D,u}_{X_1 \cdots X_L}}{\overset{k^{D,u}_{X_1 \cdots X_L} e^{-\delta\tilde{G}_{X_L}}}{\rightleftharpoons}} & D + X_1 \cdots X_L,
\end{array}
\tag{S22}
$$

where $n \leq L,\, X_i \in \{R, W\},\; X_i^* \in \{R^*, W^*\}$. As in the previous CRN, T represents the template, D the destructive catalyst, R the "right" monomers and W the "wrong" monomers. R^* and W^* are non-activated monomers. Dynamics is assumed to follow mass action kinetics, with rate constants given above and below the harpoons. The species, $TX_1 \cdots X_{n-1} \circ X_n$ ($TX_1 \cdots X_{n-1} \circ X_n^*$) represent a complex of polymer of length $n-1$ bound to the template as well as a (non-)activated monomer X_n (X_n^*) bound, but not yet polymerised into a single polymer of length n. $X_1 \cdots X_L$ are the products. Both non-activated (X^*) and activated monomers (X) bind to the template or destructive catalyst with standard free energy $-\delta\tilde{G}_X$. If a non-activated monomer is bound to the template, it may be activated, with a free-energy change $\delta\tilde{G}_{\text{act}}$. If an activated monomer is bound to the template, it may be polymerised into the growing copolymer; the standard free-energy change of polymerisation is $-\delta\tilde{G}_{\text{pol}}$. The destructive catalyst has an additional free energy $\delta\tilde{G}_f$ per length driving the disassembly of polymers.

Once again, we linearise the system by assuming that monomers and catalysts are coupled to chemostats. In figure S3, we show part of the linearised CRN graph; this fragment should be inserted into figure S1 in place of the pathway ($\emptyset \to TR \to TRR \to RR$), with similar modifications to all other template-based pathways to RW, WR, and WW. Proofreading adds complexity to the graph in the form of additional loops, and we now explicitly consider a polymerization step independently from the binding to the template.

Monomers are now present in inactive (starred) and active forms, with $-\delta\tilde{G}_{\text{act}}$, representing the free-energy change of activation. We assume that each non-activated monomer $R^*,\ W^*$ is chemostatted at the same concentration as each activated monomer $R,\ W,\ c$. Dropping this assumption would only cause a shift to $\delta\tilde{G}_{\text{act}}$. As a result, $\delta\tilde{G}_f$ and $\delta\tilde{G}_{\text{act}}$ control the concentration bounds. We assume $\delta\tilde{G}_f,\ \delta\tilde{G}_{\text{act}} \geq 0$.

The most extreme paths we have been able to identify for this system have free-energy changes

$$
\delta\tilde{G}_U^* = \left\{ \begin{matrix} \frac{L^2}{4} + \frac{L}{2} \\ \frac{(L+1)^2}{4} \end{matrix} \right\} \delta\tilde{G}_{\text{act}},
\tag{S23}
$$

and

$$\delta\tilde{G}_L^* = -\left\{ \begin{array}{c} \frac{L^2}{4} \\ \frac{L^2-1}{4} \end{array} \right\} \delta\tilde{G}_{\text{act}} - L\delta\tilde{G}_f, \tag{S24}$$

where the top value in each brace is for even L and the bottom value for odd L. Unlike the simple system in Note S5, the SAWs that exhibit $\delta\tilde{G}_U^*$ and $\delta\tilde{G}_L^*$ are not the intuitively simple pathways that go via the template and the destroyer, respectively. Instead, the pathways correspond to snaking through the CRN, alternately using both the template and destructive catalyst to first create a sequence, and then convert it into another sequence. We show these pathways for $L = 4$ in figure S4.

Since we have not formally proved that $\delta\tilde{G}_L^* = \delta\tilde{G}_L$ and $\delta\tilde{G}_U^* = \delta\tilde{G}_U$, $\Delta G \geq \Delta G^* = \delta\tilde{G}_L^* - \delta\tilde{G}_U^*$ and the "bounds" implied by $\Delta\tilde{G}^*$ are not strict bounds on system performance. Rather, they are bounds on the bounds; if we could identify $\Delta G > \Delta G^*$, then our theory would allow for even better information propagation. Nonetheless, we have not identified any sets of parameters that allow even the level of accuracy implied by ΔG^* to be achieved. The SAWs yielding $\delta\tilde{G}_U^*$ and $\delta\tilde{G}_L^*$ would not only be absurd as the dominant pathways in a real system, they actually cannot dominate production and degradation, even in principle. Since the pathways exhibiting $\delta\tilde{G}_U^*$ and $\delta\tilde{G}_L^*$ pass through other products as intermediates, scaling these pathways to be fast would necessarily result in sub-pathways to other products being fast.

Despite the above impossibility, the existence of these snaking pathways can provide some advantage, at least in principle. In figure S5, we show two attempts to find parameters that minimize entropy for a system with $L = 4$. In the first "naïve" scheme, plotted in blue, reactions contributing to assembly of $RRRR$ via the template or assembly of any other sequence via the destructive catalyst are assigned rates of 1 and all other rates are taken as $\sim k$. We show this scheme in figure S6. In the second "best guess" scheme, plotted in red, we make use of these snaking pathways. The reactions contributing to assembly of $RRRR$ via the template are still assigned rates of 1. However, for all other products, the longest snaking pathway which does not intersect with the pathway leading to $RRRR$ has rates assigned 1. We show this scheme in figure S7.

As $k \to 0$, the snaking pathways outperform the non-snaking pathways significantly, resulting in approximately half the entropy of the product distribution (figure S5). Notably, however, neither the best guess nor the naïve system converges on the minimal entropy of a system with a free-energy difference of $\Delta\tilde{G}^*$ between pathways as $k \to 0$; the best guess approaches $H = 1.8 \times 10^{-2}$ nats as compared to the bound at $H_{\min} = 1.9 \times 10^{-4}$ nats. Although the best guess system can outperform the naïve system in principle, when absolute rates can be chosen freely, rates may in practice be mechanistically constrained. As a result, this outperformance may not be achievable in a specific model. Indeed, at moderate k, the entropies of the naive system and the best guess converge.

Note S7 Example DNA strand displacement system that could maximise specificity in pseudo-equilibrium

Consider a molecular system in which two monomer species M_1 and M_2 can be catalytically activated into M_1^* and M_2^*. Such a setup could describe two enzymes that are activated by selective kinase-based phosphorylation, and deactivated by phosphatases. The system exhibits a maximally specific product ensemble in pseudo-equilibrium, analogous to the result obtained for catalytic assembly of polymers.

To see this behaviour, assume K and P catalyse activation with free-energy changes $\delta\tilde{G}_K$ and $\delta\tilde{G}_P$ along the respective pathways for both Monomer 1 and Monomer 2. Without loss of generality, assume $-\delta\tilde{G}_K > -\delta\tilde{G}_P$; informally, K tends to activate while P tends to deactivate. Then the steady state ratio of activated to deactivated Monomer 1 is given by

$$\frac{[M_1^*]}{[M_1]} = \frac{k_1^K + k_1^P}{k_{-1}^K + k_{-1}^P} = \exp(-\delta\tilde{G}_p) \frac{\frac{k_1^K}{k_1^P} + 1}{\frac{k_1^K}{k_1^P} \exp(\delta\tilde{G}_K - \delta\tilde{G}_p) + 1}, \tag{S25}$$

where k_1^K (k_{-1}^K) is the rate (absorbing the catalyst concentration) at which catalyst K activates (deactivates), and Monomer 1, k_1^P (k_{-1}^P) is the rate at which catalyst P activates (deactivates) Monomer 1, and we have used the detailed balance constraints $\frac{k_1^K}{k_{-1}^K} = \exp(-\delta\tilde{G}_K)$ and $\frac{k_1^P}{k_{-1}^P} = \exp(-\delta\tilde{G}_P)$. Equivalently,

$$\frac{[M_2^*]}{[M_2]} = \frac{k_2^K + k_2^P}{k_{-2}^K + k_{-2}^P} = \exp(-\delta\tilde{G}_p) \frac{\frac{k_2^K}{k_2^P} + 1}{\frac{k_2^K}{k_2^P} \exp(\delta\tilde{G}_K - \delta\tilde{G}_p) + 1}. \tag{S26}$$

Here, subscript "2" indicates that the rate is for Monomer 2's interconversion, and we have applied the same thermodynamic constraints as for Monomer 1.

Assume (without loss of generality) that we wish to maximise $[M_1^*]/[M_2^*]$ – the presence of the catalysts K and P is intended to activate M_1 and not M_2. Since $\exp(\delta\tilde{G}_K - \delta\tilde{G}_p) < 1$, we can immediately see that eq. S25 is maximised by allowing $k_1^K/k_1^P \to \infty$, in which case $\frac{[M_1^*]}{[M_1]} \to \exp(-\delta\tilde{G}_K)$. Similarly, eq. S26 is minimised by allowing $k_2^K/k_2^P \to 0$, in which case $\frac{[M_2^*]}{[M_2]} \to \exp(-\delta\tilde{G}_P)$. This optimal specificity for activation of Monomer 1 corresponds to Monomer 1 in a pseudo-equilibrium determined by the path coupled to K, and Monomer 2 in a pseudo-equilibrium determined by the path coupled to P. If we further assume, as in the main text, that inactive monomer concentrations are chemostatted at the same value, we obtain an optimal $\frac{[M_1^*]}{[M_2^*]} = \exp(-(\delta\tilde{G}_K - \tilde{G}_P)) = \exp(\Delta\tilde{G})$ as before.

A system directly analogous to the one described above can be designed using existing motifs from DNA nanotechnology[S1,S2], allowing pseudo-equilibrium systems to be studied in a concrete experimental setting. The mechanism, illustrated in figure S8, is based on 4-way strand exchange and allows fine tuning of the thermodynamic drive on each pathway in a way that is hard to achieve with, for example, ATP turnover.

Supplemental References

1. Mullor Ruiz, I. Development of a framework for designing nucleic acid-based, out-of-equilibrium catalytic reaction networks. PhD Thesis Imperial College London London, UK (2021).

2. Lankinen, A., Mullor Ruiz, I., and Ouldridge, T.E. (2020). Implementing Non-Equilibrium Networks with Active Circuits of Duplex Catalysts. In C. Geary, and M.J. Patitz, eds. 26th International Conference on DNA Computing and Molecular Programming (DNA 26). Schloss Dagstuhl – Leibniz-Zentrum für Informatik pp. 7:1–7:25.

3. Nam, K.M., Martinez-Corral, R., and Gunawardena, J. (2022). The linear framework: using graph theory to reveal the algebra and thermodynamics of biomolecular systems. Interface Focus *12*, 20220013.

4. Robinson, S.M. (1970). A short proof of Cramer's rule. Mathematics Magazine *43*, 94–95.

5. Maes, C., and Netočný, K. (2013). Heat bounds and the blowtorch theorem. Ann. Henri Poincaré *14*, 1193–1202.

6. Sáez, M., Feliu, E., and Wiuf, C. (2019). Linear elimination in chemical reaction networks. In J.L. García Guirao, J.A. Murillo Hernández, and F. Periago Esparza, eds. Recent Advances in Differential Equations and Applications pp. 177–193.. Springer International Publishing pp. 177–193.

7. Çetiner, U., and Gunawardena, J. (2022). Reformulating nonequilibrium steady states and generalized hopfield discrimination. Phys. Rev. E *106*, 064128.

8. Arunachalam, E., and Lin, M.M. (2025). Information Gain Limit of Biomolecular Computation. Phys. Rev. Lett. *134*, 148401.

9. Ouldridge, T.E. (2018). The importance of thermodynamics for molecular systems, and the importance of molecular systems for thermodynamics. Nat. Comput. *17*, 3–29.

10. Cover, T.M., and Thomas, J.A. (2006). Elements of information theory. Wiley-Interscience.

11. Polettini, M., and Esposito, M. (2014). Irreversible thermodynamics of open chemical networks. i. emergent cycles and broken conservation laws. J. Chem. Phys. *141*, 024117.

12. Qureshi, B., Juritz, J., Poulton, J.M., Beersing-Vasquez, A., and Ouldridge, T.E. (2023). A universal method for analyzing copolymer growth. J. Chem. Phys. *158*, 104906.

13. Poulton, J.M., Ten Wolde, P.R., and Ouldridge, T.E. (2019). Nonequilibrium correlations in minimal dynamical models of polymer copying. Proc. Natl. Acad. Sci. U.S.A. *116*, 1946–1951.

14. Hopfield, J.J. (1974). Kinetic proofreading: a new mechanism for reducing errors in biosynthetic processes requiring high specificity. Proc. Natl. Acad. Sci. U.S.A. *71*, 4135–4139.

15. Ninio, J. (1975). Kinetic amplification of enzyme discrimination. Biochimie *57*, 587–595.

16. Bennett, C.H. (1979). Dissipation-error tradeoff in proofreading. BioSystems *11*, 85–91.

17. Mallory, J.D., Igoshin, O.A., and Kolomeisky, A.B. (2020). Do we understand the mechanisms used by biological systems to correct their errors? J. Phys. Chem. B *124*, 9289–9296.
